# Partisan conflict over content moderation is more than disagreement about facts

**DOI:** 10.1126/sciadv.adg6799

**Published:** 2023-11-03

**Authors:** Ruth E. Appel, Jennifer Pan, Margaret E. Roberts

**Affiliations:** ^1^ Department of Communication, Stanford University, Stanford, CA 94305, USA.; ^2^ Department of Political Science and Halıcıoğlu Data Science Institute, University of California, San Diego, CA 92093, USA.

## Abstract

Social media companies have come under increasing pressure to remove misinformation from their platforms, but partisan disagreements over what should be removed have stymied efforts to deal with misinformation in the United States. Current explanations for these disagreements center on the “fact gap”—differences in perceptions about what is misinformation. We argue that partisan differences could also be due to “party promotion”—a desire to leave misinformation online that promotes one’s own party—or a “preference gap”—differences in internalized preferences about whether misinformation should be removed. Through an experiment where respondents are shown false headlines aligned with their own or the opposing party, we find some evidence of party promotion among Democrats and strong evidence of a preference gap between Democrats and Republicans. Even when Republicans agree that content is false, they are half as likely as Democrats to say that the content should be removed and more than twice as likely to consider removal as censorship.

## Teaser

Partisan conflict over content moderation is not only due to disagreement about facts, but also to divergence in preferences.

## INTRODUCTION

Misinformation is seen as a major global threat by political and economic leaders around the world (*1*) as well as by the general public (*2*, *3*). Rising public awareness of online misinformation has coincided with growing public debates about what social media companies should remove from their platforms. These debates have laid bare deep partisan divisions over the removal of online content in the United States. Both Republicans and Democrats have called for the repeal of Section 230 of the Communications Decency Act, which protects social media companies from liability for content on their platforms. But the two sides of the aisle have very different views about how the act should be reformed (*4*). This divide has led to partisan gridlock over policies to combat misinformation. For example, the Biden administration’s creation of the Disinformation Governance Board under the Department of Homeland Security was paused by Republican objections over its mission just 3 weeks after its announcement (*5*). Partisan consensus over content moderation would empower social media companies to more effectively regulate what content should be permitted online. In contrast, conflict over content moderation puts both social media companies and regulators in a bind, as any decision is unpopular. Given that many large global social media platforms—such as Facebook, YouTube, WhatsApp, and Instagram—are based in the United States, U.S. content moderation policies may also influence content removal and moderation in other countries, some of which face their own partisan divisions (*6*). Similar to how EU regulation has a global impact in policy areas such as privacy (*7*), U.S. content moderation policies may also have global implications.

There is a large literature on content moderation (*8*, *9*), which has documented partisan differences in support for content removal (*10*, *11*). The most prominent explanation for this partisan disagreement over content moderation is what we call the “fact gap”—the idea that partisanship influences what individuals believe to be true (*12*, *13*) and hinders their ability to identify content aligned with their political views and ideology as misinformation (*14*–*21*). This fact gap could be driven by psychological mechanisms, including mechanisms for preserving one’s identity (*22*, *23*) or complementary beliefs (*24*–*26*), such as motivated reasoning, prior attitude effect, and confirmation bias, as well as more general cognitive mechanisms like inattention (*27*–*29*).

Here, we theorize and test for the existence of two additional potential sources of partisan disagreement over what content social media companies should remove from the internet beyond the fact gap: “party promotion” and a “preference gap.” Although the fact gap is important and consequential, it may not fully explain the sizeable partisan gaps that have been identified. Existing research suggests that analyzing factors beyond disagreement about facts may be important to understanding partisan disagreement over content moderation. For example, Kozyreva *et al*. (*10*) find a 30% partisan difference in preferences to remove content that denies the Holocaust and note that accuracy perceptions alone cannot explain such partisan differences.

We define party promotion as the desire to leave misinformation online when it benefits one’s own party or denigrates the other party, and to remove misinformation that denigrates one’s own party or promotes the other party, regardless of belief in the accuracy of the information. Partisanship might lead to such behavior due to the importance of the symbolic social standing of one’s own party (in-group) relative to the other party (out-group) (*30*). In the United States, party promotion may be a plausible potential explanation for conflict over what content should be moderated given that affective polarization—the gap in affect toward the partisan in-group and the partisan out-group—has increased (*31*, *32*). Studies have identified phenomena similar to party promotion in the United States in related settings. For example, partisan alignment affects the demand for biased news (*33*) and predicts misinformation sharing (*9*). Content flagging has also been shown to be used strategically at times to promote one’s own political aims rather than due to genuine belief (*34*).

Even in the absence of a fact gap or party promotion, partisans may disagree about whether content should be removed because of differences in internalized preferences. This preference gap implies that there might be partisan differences in overall preferences for content removal on the internet, regardless of which party the specific content advantages and even if partisans agree that specific content is misinformation. This gap in preferences could stem from (a) differences in internal factors like identity or core values, which are deeply rooted and difficult to change, or (b) internalization of elite cues and signals, which may be more changeable. In terms of (a), people differ in identity, values, personality, cognitive processes, motives, and emotions (*22*, *23*, *35*–*41*). If people select into political parties based on these internal factors, then these divergent underlying factors could account for a partisan preference gap.

The preference gap could also result from (b), people internalizing cues and signals from elites in the party they identify with as their own preferences (*42*–*44*). Democratic and Republican elites have emphasized free speech as a core value in different periods of American history and on different issues (*11*, *45*, *46*) [see also section S2.3 for frequency of congressional speeches containing censorship-related keywords by party from the 46th (1879) to 116th U.S. Congress (2021)]. For example, Lynn Woolsey, a Democratic House member at the time, commented that increasing the concentration of media ownership could result in censorship in a 2003 speech (*47*). In early 2023, Republican House member Nicholas Langworthy expressed concern that Big Tech companies were censoring conservative voices (*48*). In recent years, Republican elites have framed online content removal as a free speech and censorship issue (*49*, *50*), while Democratic elites have generally been supportive of the need for content moderation. Note, however, that during this same time period, Democrats have expressed concern about censorship in other areas such as textbook bans [see speech by Democratic House member Jeremy Raskin in March 2023 (*51*)]. Such elite signaling may result in a preference gap because Republicans, knowing that party elites are opposed to content removal on the internet, may base their preferences on elite signals and prefer that content remains online, while Democrats, knowing that party elites support content moderation, may prefer removal of misinformation. Recent surveys show that Republicans place higher importance on free speech rights on the internet than Democrats, while Democrats place higher importance on preventing the spread of false information online than Republicans (*46*). Note that while this behavior appears similar to promoting one’s own party, it reflects an internalized overall preference toward content moderation regardless of the partisan slant of the information. Thus, it differs from our concept of party promotion, which is strategic behavior that treats content online differently depending on its partisan slant.

### Design and data

To test whether partisan conflict over content moderation may arise from the preference gap and party promotion, we embedded an experiment in a national survey of U.S. respondents. We attempt to neutralize the fact gap by presenting participants with misinformation headlines and explicitly telling respondents they are false. We then disaggregate the effects of the preference gap and party promotion by varying the partisan alignment of the headline.

Our survey of U.S. adults was commissioned by the Knight Foundation and fielded by Ipsos in the summer of 2021. The survey was implemented on the Ipsos KnowledgePanel, which is described by Ipsos as a representative random sample (for descriptive statistics comparing the sample to the U.S. population, see section S1.7). For our analysis, we focus on English-speaking respondents who identified as Democrat or Republican, resulting in 1120 respondents, with a mean age of 53.29 (SD = 16.53) and 56.3% female (see section S1.7 for detailed descriptive statistics). The experiment and analyses were preregistered (see data accessibility statement in the Acknowledgments for details; see deviations and clarifications from the pre-analysis plan in section S1.2 and throughout the supplementary materials text where they pertain). This research was approved by the Institutional Review Boards at our respective universities.

The survey experiment relied on simple randomization at the participant and at the headline level. Each participant was shown two different false news headlines sequentially (for a flow diagram of the experiment, see section S1.1). Respondents were told that “Someone has shared the following headline on a social media site. (This headline has been established as **false** by third-party fact checkers.).” One of the headlines aligned with the respondent’s partisanship, while the other headline was not aligned with the respondent’s partisanship. For example, one pro-Republican headline (aligned for Republicans, misaligned for Democrats) reads: “Hours after signing an executive order on Jan. 20, 2021, U.S. President Joe Biden violated his own mask mandate.” Whether the respondent saw the aligned or misaligned headline first was randomized. Headlines were selected from a bank of 18 news headlines (9 aligned for Democrats, 9 aligned for Republicans) that contained false claims. We provide more information on headline selection in Materials and Methods.

We measured three main outcomes: (i) *Intent to remove headline (removal)*: Whether or not the participant states that the headline should be removed by the social media company; (ii) *Perception of headline removal as censorship (censorship)*: Whether the participant considers the removal of the headline censorship; (iii) *Intent to report headline as harmful (harm)*: Whether the participant would report the headline as harmful content on a social media platform. We also measure a range of covariates, including perceived accuracy. To measure perceptions of accuracy, we ask respondents for their perceived accuracy of the false news headlines on a four-point scale. All measures, including control variables and indices, are described in detail in section S1.6.

We analyze results using OLS regression, interacting partisanship of participants and political alignment of the headlines:

$$Y_{ia}=\beta_{D}D_{i}\cdot {Hd}_{a}+\beta_{R}R_{i}\cdot {Hr}_{a}+\gamma_{D}D_{i}+\gamma_{R}R_{i}+\varepsilon_{ia}$$ (1)

where *Y_ia_* is the binary outcome measure for individual *i* and headline *a*. *D_i_* indicates that respondent *i* is a Democrat and *R_i_* indicates that respondent *i* is a Republican. The difference in coefficients on *D_i_* and *R_i_* reflects the preference gap or the amount overall that Democrats and Republicans disagree about whether false content should be removed controlling for alignment. *Hd_a_* is an indicator of whether headline *a* is aligned for Democrats and *Hr_a_* is an indicator of whether headline *a* is aligned for Republicans. The coefficients on *D_i_ · Hd_a_* and *R_i_ · Hr_a_* reflect party promotion or the amount that the outcome depends on the alignment between the partisan nature of the content and the respondent for Democrats and Republicans, respectively (see section S1.3 for additional details on our analyses).

## RESULTS

We find a large and statistically significant difference between the content moderation preferences of Republicans and Democrats. Overall, the probability that Democrats say a false headline should be removed is 0.69, while the probability that Republicans say a false headline should be removed is 0.34. The probability that Democrats would report a false headline as harmful is 0.49, while for Republicans, it is 0.27. The probability that Democrats perceive the removal of false headlines as censorship is 0.29, while for Republicans, it is 0.65 (see tables S12 to S14 for regressions that calculate these probabilities).

**Fig. 1.**
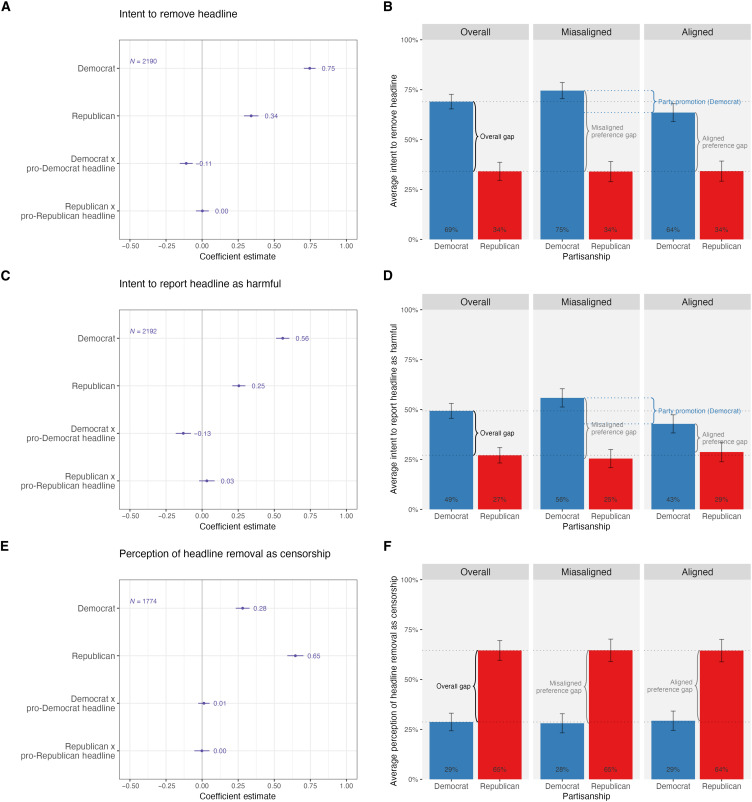
Partisanship and preferences for content moderation for all respondents. No control variables; 95% confidence intervals are shown. The left panels show the coefficient estimates and confidence intervals from Eq. 1 for all respondents and the removal (**A**), harm (**C**), and censorship (**E**) outcome. The right panels show the same estimates for all respondents along with the overall gap between partisans for the removal (**B**), harm (**D**), and censorship (**F**) outcome (see section S1.3.1 for details).

The left panels of Fig. 1 plot the coefficient estimates and confidence intervals from Eq. 1 for all respondents and each of the three outcomes. The right panels of Fig. 1 present the same estimates along with the overall gap between partisans to illustrate the relative sizes of party promotion and the preference gap (see section S1.3.1 for details). The preference gap for the removal outcome is the difference between Democrats’ and Republicans’ support for removal, controlling for alignment. Party promotion for the removal outcome is the difference between Democrats’ and Republicans’ support for the removal of aligned versus misaligned headlines.

Looking at the intent to remove headline outcome (Fig. 1, A and B), we can see that for misaligned headlines, the probability of intent to remove is 0.75 for Democrats, while it is 0.34 for Republicans, resulting in a misaligned preference gap of 0.41. For Republicans, there is no difference in their intent to remove misaligned and aligned headlines (no party promotion), but there is party promotion for Democrats (intent to remove aligned headlines declines by 0.11 to 0.64). For the intent to report headline as harmful outcome (Fig. 1, C and D), we also see a sizable preference gap between Democrats and Republicans (0.30 preference gap for misaligned headlines), some party promotion among Democrats who are less likely (−0.13) to report aligned headlines as harmful than misaligned headlines, and no party promotion among Republicans who are equally willing to report aligned and misaligned headlines as harmful. For the perception of headline removal as a censorship outcome (Fig. 1, E and F), there is no evidence of party promotion—no difference between misaligned versus aligned headlines among Democrats or Republicans—but a large preference gap between Democrats and Republicans (−0.37 for misaligned headlines).

### Persistence of the fact gap

**Fig. 2.**
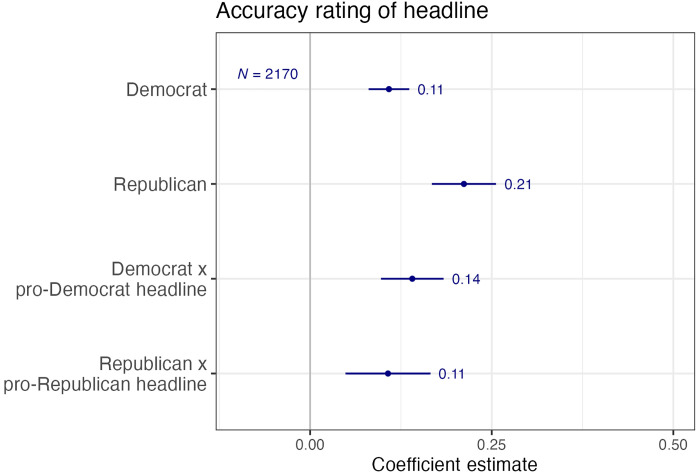
Respondents’ assessment of headline accuracy. No control variables.

While we inform respondents that the headlines have been rated as false by third-party fact-checkers, respondents rated 20.32% of headlines as either “very accurate” or “somewhat accurate.” Moreover, consistent with previous literature, the interaction terms in Fig. 2 show that evaluations of the accuracy of the headline are partisan—both Democrats and Republicans are more likely to think that headlines that align with their own position are true, reflecting the persistence of the fact gap despite explicit information we provided that the headlines are false. From Fig. 2, we see that Democrats rate 11% of pro-Republican and 25% (11% + 14%) of pro-Democrat headlines as accurate. Similarly, Republicans rate 21% of pro-Democrat headlines and 32% (21% + 11%) of pro-Republican headlines as accurate.

If some respondents still believed that the headlines were true, despite being told they were false, this poses a problem of identification. It means that we cannot fully isolate the effects of party promotion and the preference gap from the effect of the fact gap. To address this, we conducted three additional analyses, two of which were preregistered. If the results of all three tests are consistent, then it would give us further confidence that we can measure the contribution of party promotion and the preference gap to partisan disagreement over content moderation, controlling for the fact gap. For more information on each method, see Materials and Methods.

### Inaccurate subgroup analysis

**Fig. 3.**
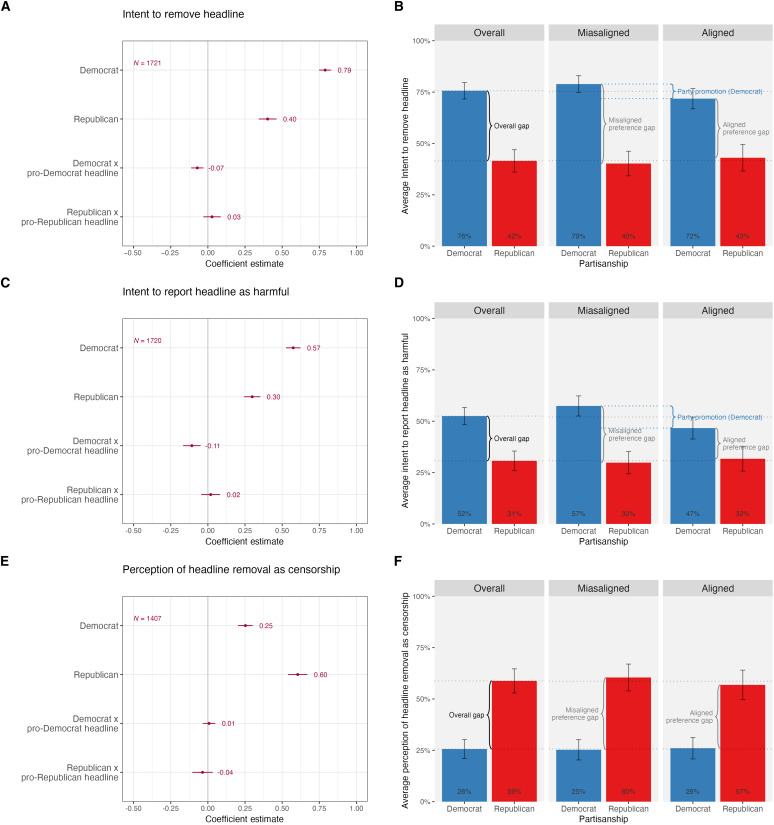
Partisanship and preferences for content moderation for respondents who agree headlines are inaccurate. No control variables; 95% confidence intervals are shown. The left panels show the coefficient estimates and confidence intervals from Eq. 1 for respondents who agree headlines are inaccurate and the removal (**A**), harm (**C**), and censorship (**E**) outcome. The right panels show the same estimates for respondents who agree headlines are inaccurate along with the overall gap between partisans for the removal (**B**), harm (**D**), and censorship (**F**) outcome (see section S1.3.1 for details).

In the first analysis to address this persistent fact gap, we subset to respondents who rated the headlines as inaccurate, including respondents who assessed headlines as “not very accurate” or “not at all accurate.” Figure 3 shows that when we subset to respondents who agree the false headlines are inaccurate, the preference gap results stay the same. Democrats are still nearly twice as likely as Republicans to want to remove the headline and report the headline as harmful, and half as likely to perceive removal as censorship. While Republican respondents still exhibit the same preferences on all three outcomes regardless of whether the false headline is aligned or misaligned with their political views, party promotion among Democrats is slightly smaller among the inaccurate subgroup. This suggests that some of the party promotion in the main results may have been a result of the fact gap. However, there is still a significant effect of party promotion among Democrats, suggesting that factual beliefs do not completely explain away this effect.

A shortcoming of this approach, which means we cannot fully account for the fact gap, is that Democrats and Republicans who believe the headline to be inaccurate are potentially differentially selected. We find that on observables such as gender, age, income, and education, Democrats and Republicans who assess headlines as inaccurate are not significantly different from Democrats and Republicans in the overall sample (see tables S3 and S4). However, there could still be differences in unobservables between those who assess headlines to be inaccurate and the broader respondent pool.

### Consensus headlines analysis

In the second analysis, we conducted the main analyses for headlines that, on average, both Republicans and Democrats think are inaccurate and where there is little difference in accuracy perception between Democrats and Republicans (see section S2.1.5). We identify these “consensus headlines” by limiting the mean absolute difference between the average Democrat and Republican accuracy rating for headlines that both Democrats and Republicans on average rate as inaccurate to 0.5 on the four-point accuracy scale. This results in eight headlines. When we decrease this threshold, reducing the number of headlines, the substantive results remain unchanged; see section S2.1.5. We add this analysis to attempt to address the concern that the gap in support for removal observed among Democrats and Republicans in the previous analyses is driven by headlines with a large gap in perceived accuracy between Democrats and Republicans (see also results disaggregated by headlines in section S2.1.6).

While a limitation of this robustness check is that it selects only very few headlines and therefore may not generalize, at least for this set of headlines, we continue to observe evidence for the preference gap when we hone in on headlines Democrats and Republicans agree are inaccurate. Democrats remain nearly twice as likely as Republicans to want to remove content and to report content as harmful, while Republicans are nearly twice as likely as Democrats to consider removal censorship. We also continue to see evidence of party promotion among Democrats.

### Accuracy as mediator

In the third analysis, we examine the extent to which party promotion among Democrats and the preference gap are mediated by belief in the accuracy of the content. For the mediation effect for party promotion, we conducted a mediation analysis of the effect of alignment between respondent and headline partisanship on the outcomes for Democratic respondents. The mediation analysis was preregistered for party promotion. We also conducted a mediation analysis for the preference gap, which was not preregistered. For the mediation effect of the preference gap, we conducted a mediation analysis of the effect of Democrat partisanship on the outcomes for all respondents. For more details on the mediation analyses, see sections S2.2.1 and S2.2.2.

Table 1 shows the results of the analysis for party promotion. The estimand in this analysis is the average causal mediation effect (ACME) (*52*). ACME is the total effect that alignment has on the outcome variable of interest minus the average direct effect (ADE), which is the effect of alignment on the outcome without taking the indirect path through accuracy into account.

**Table 1.** Effect of alignment mediated by accuracy for Democrats. Note: Mediation models were run with standard errors clustered on participants, without weighting observations and without control variables using a dataset in which missing values were addressed using listwise deletion. ACME, average causal mediation effect; ADE, average direct effect.

Measure	Estimate	*P* Value
**Intent to remove headline**		
ACME	−0.065	<0.001
ADE	−0.039	0.034
Total effect	−0.103	<0.001
Proportion mediated	0.624	<0.001
*N* Observations	1302	
*N* Simulations	1000	
**Intent to report headline as harmful**		
ACME	−0.035	<0.001
ADE	−0.074	<0.001
Total effect	−0.109	<0.001
Proportion mediated	0.321	<0.001
*N* Observations	1301	
*N* Simulations	1000	

In the main analysis (Fig. 1), we saw that Democrats were less likely to intend to remove a headline or report a headline as harmful for headlines aligned with their partisanship. In Table 1, we see that this effect may, in part, be mediated by accuracy. ACME and ADE are negative and significant for both intent to remove the headline and intent to report the headline as harmful (for alternative specifications, see tables S25 and S26). This suggests that, while the party promotion effect for Democrats is reduced when accounting for perceptions of accuracy, the fact gap may not completely explain away party promotion among Democrats on these outcomes.

Note that ACME is only identified under a sequential ignorability assumption that (i) given the observed pretreatment confounders, the treatment assignment is statistically independent of potential outcomes and potential mediators and (ii) the mediator is ignorable given observed treatment and pretreatment confounders. While treatment is randomly assigned, the second assumption may not hold because the mediator, perception of accuracy, is not. In other words, there may be characteristics of respondents that affect both whether they think a headline is accurate and whether they think a headline should be removed. To probe the robustness of the findings, we conducted a sensitivity analysis (*53*). The sensitivity analysis indicates that our conclusion, that accuracy mediates party promotion but cannot completely explain away party promotion for Democrats, is plausible given even fairly large departures from the ignorability of the mediator due to pretreatment confounders (see section S2.2 for additional details).

As described in section S2.2.2, we also conduct a mediation analysis for the preference gap. We find that the preference gap is largely a direct effect of partisanship on content moderation preferences, only mediated by accuracy by a small amount. We also conducted a sensitivity analysis and find that this direct effect is robust to very large departures from the ignorability of the mediator due to pretreatment confounders.

We acknowledge that partisan disagreement on headline accuracy poses a problem of identification. However, the results of the three additional analyses that we conducted to address the fact gap are consistent and suggest that the preference gap and, to a smaller extent, party promotion explain a portion of partisan differences in content moderation preferences.

## DISCUSSION

In line with existing research on content moderation, we find strong partisan differences in content moderation preferences. However, the results of this experiment highlight a need to consider factors beyond the fact gap to understand these partisan differences. While prior research has established the importance of the fact gap in explaining content moderation preferences, our experiment shows that the preference gap likely also affects attitudes toward the removal of misinformation online. In the United States today, Democrats prefer to remove misinformation, while Republicans prefer to avoid the removal of misinformation and perceive such removal as censorship, even when they agree that the content is inaccurate. In addition, this study provides a previously unexplored perspective on debates related to content moderation because although the term “censorship” is used in major political debates in the United States, most studies related to censorship perceptions were conducted before the social media era [for examples and exceptions, see (*22*, *45*, *54*, *55*)].

There are limitations to this study that highlight the need for future research to examine the causes of the preference gap, to study this dynamic at other points in time and in other political contexts, to measure how these findings generalize from a survey experiment to social media platforms, and to look at content beyond the political misinformation examined here. The preference gap could arise from deeply rooted internal factors such as moral values or from internalization of elite cues. Differentiating between these factors has important policy implications because internal factors like moral values are difficult to move, while elite cues may be more likely to change over time. Fifteen years ago, Lindner and Nosek (*11*) found that Democrats had stronger preferences for protecting free speech than Republicans, perhaps suggesting that more changeable factors may be at work, but additional empirical assessments are needed. This underscores the need to study these dynamics at different points in time and in other political contexts. For example, future research could pursue a similar experiment in contexts where those on the right are more supportive of content moderation and those on the left oppose it (*56*).

In our experiment, we balanced the partisanship of headlines and kept other headline characteristics and their context relatively comparable. On social media platforms, however, Republican-aligned misinformation is more common (*57*). It could be that this difference in the prevalence of misinformation drives differences in the content moderation preferences of Democrats and Republicans. For example, Republicans might have developed lower baseline moderation preferences because they think that the content moderation system disproportionately targets them. Alternatively, if Republicans’ threshold for unfollowing users mimics their high threshold for removing content, then our findings could also explain why conservatives are exposed to more misinformation in general (*58*–*62*). Future research could explore this in more detail.

Experimenter demand effects, social desirability bias, and the higher cost and benefit of taking action in a real-world setting could affect our results. While the action of flagging content on social media is similar to clicking on a button in a survey, users may experience such actions differently on social media knowing that their actions may have tangible, real-world consequences. In this study, we did not use incentivized responses to address these issues. This experiment was part of a larger collaborative survey, thus we did not have the opportunity to provide incentives. Furthermore, there is debate over how incentivized responses influence studies of partisan differences (*63*), with some finding reductions in partisan differences (*13*, *64*) and others arguing that such designs are not required to capture how people in the real world evaluate and make decisions (*16*, *65*). Future work could explore to which extent our results generalize beyond a survey context, for example, by embedding a similar experiment within a social media platform or adding treatment groups with incentivized responses such as a willingness-to-pay design.

Last, the content (e.g., political versus health misinformation) and context (e.g., motivation to seek out the truth, how rooted beliefs about a topic are in one’s identity) of misinformation headlines also matter (*10*, *22*, *23*, *46*, *66*). In this study, we focus on political headlines, and more specifically those denigrating out-party politicians instead of flattering in-party politicians. Future research should investigate further how other types of content—political misinformation flattering in-party politicians, nonpolitical misinformation, hate speech, voter suppression content—and different contexts influence the preference gap and party promotion (*67*). Another potentially interesting research question is to what extent individual-level drivers of content moderation are decisive at the level of content moderation systems with thousands of often professional content moderators, and which other factors might be at play in those systems.

In terms of the implications of these findings, it is encouraging that the effects of party promotion are dwarfed by the preference gap. In an environment with increasing partisan animosity, respondents—Republicans in particular—seemed to evaluate content removal outside of the lens of party promotion. Policymakers and social media platforms could consider different approaches to design policies with bipartisan support. First, thinking about content moderation as a system of procedures applied at scale, rather than decisions on individual pieces of content by individual moderators (*68*), might help by shifting the focus from specific content to be moderated to a system of procedures that needs to be agreed upon. For this system, the preference gap might be less pronounced than for specific content. Second, future research could explore whether there might be a partisan consensus on less extreme forms of content moderation, like flagging or down-weighting misinformation. Third, policymakers could attempt to use moral reframing, the practice of tailoring content to an individual’s moral values by framing a position an individual would usually oppose in a way that is consistent with their moral values (*69*), to bridge the preference gap to the extent that it is rooted in moral value differences.

Policymakers and social media platforms should understand that differences between Democrats and Republicans stem from more than just disagreement over what is true versus false and strategic partisan maneuvering. Instead, Americans seem to have diverging preferences about the concept of content removal and whether the protection of free speech necessitates or precludes the moderation of content.

## MATERIALS AND METHODS

### Sample

Our sample consisted of U.S. adults recruited by Ipsos, which is a market research firm based in France with worldwide operations. Ipsos is nonpartisan and we have no indication that U.S. respondents perceived it as having biases, ideological or otherwise. Following the exclusion criteria laid out in our pre-analysis plan, we only included participants who indicated that their preferred language was English and excluded participants who self-identified as independents because the alignment treatment would not work for independents. As noted in section S1.2, the pre-analysis plan did not specify how we would address unexpected missing values or participants from a different sample, thus we added clarifications for the following actions: We excluded participants who had missing values for partisanship or indicated values other than Democrat, Republican, or independent. Another 243 participants were part of a student sample that was different from the sample meant to be representative of the U.S. population, and we therefore excluded them from our analysis. The data were weighted with the weights provided by Ipsos for the models presented in the main text, but we also report unweighted results in section S2. See section S1.7 for detailed descriptive statistics.

### Headline selection

We identified headlines used in the experiment from the fact-checking website Snopes.com with the criteria that the headline included a clear “false” label (not partially or entirely true), political content, a clear partisan slant, and was recent. We selected headlines that were relatively balanced in terms of the intensity of the information conveyed (e.g., level of violence) and the topic. We then used a pretest to ensure that candidate headlines had a partisan alignment in the expected direction and to measure other headline characteristics including the perceived intensity (e.g., how worrying the headline is). Although these headlines may differ in other dimensions that we did not assess and may not be perfectly comparable, the final selection of headlines included pro-Democrat and pro-Republican headlines with the expected ideological slant that were relatively balanced in terms of perceived intensity and topic (see section S1.5 for additional details).

### Outcome measure details

All outcome measures are binary with the exception of the censorship measure, which was recoded as a binary measure by considering “Yes” as 1, “No” as 0, and “Do not know” as a missing value. We deviated from the pre-analysis plan in recoding “Do not know” as a missing value instead of 0 because recoding “Do not know” as 0 would have imposed a strong assumption that undecided participants actually did not think of headline removal as censorship. We provide results for the main models with the original coding as a robustness check in the Supplementary Materials (see section S2.1.2), and find that the main results remain the same.

### Accuracy measure

To measure perceptions of accuracy, we asked respondents for their perceived accuracy of the false news headlines on a four-point scale. We randomized whether participants first answered the question about accuracy or the outcome questions after the headline throughout the experiment (see fig. S1). This also allows us to ensure that our results were not driven by an “accuracy nudge” (*28*, *29*). Participants had a 50% chance of being asked the perceived accuracy question before any outcome variables were measured and a 50% chance of being asked the perceived accuracy question after the outcome variables of removal and censorship were measured (see section S1.4.2 for balance tables, section S2.1.3 for analyses with the first headline only, and tables S22 to S24 for the accuracy order analysis). The measure for harm was always asked last because we did not want to influence accuracy ratings by priming participants to think about harm.

### Covariates and indices

We measured a range of covariates such as news consumption and demographics as detailed in section S1.6. Control variables include age, gender, education, race, ethnicity, household income, political interest, whether social media was the most common news source, and whether a participant’s posts had ever been flagged or removed from social media. The order of response options in several questions on covariates, such as partisanship, was randomized. Some of the control variables that we include in our regressions are measured by multiple survey questions. For such questions, we used composite indices as detailed in section S1.6.

## Supplementary Materials

### This PDF file includes:

Supplementary Text Figs. S1 to S21 Tables S1 to S28 References
