# Supplementary material for: Partisan conflict over content moderation is more than disagreement about facts
Source: Sci Adv. 2023 Nov 3;9(44):eadg6799. doi: 10.1126/sciadv.adg6799 (PMC10624338; doi:10.1126/sciadv.adg6799)
Supplement: Supplementary file 1 — Supplementary Text Figs. S1 to S21 Tables S1 to S28 References [file sciadv.adg6799_sm.pdf]

Science Advances
AAAS

Supplementary Materials for

Partisan conflict over content moderation is more than disagreement about facts

Ruth E. Appel *et al.*

Corresponding author: Margaret E. Roberts, meroberts@ucsd.edu.

Sci. Adv. **9**, eadg6799 (2023)
DOI: 10.1126/sciadv.adg6799

This PDF file includes:

Supplementary Text
Figs. S1 to S21
Tables S1 to S28
References

Supplementary Text

S1 Extended Materials and Methods

S1.1 Experiment Flow Diagram

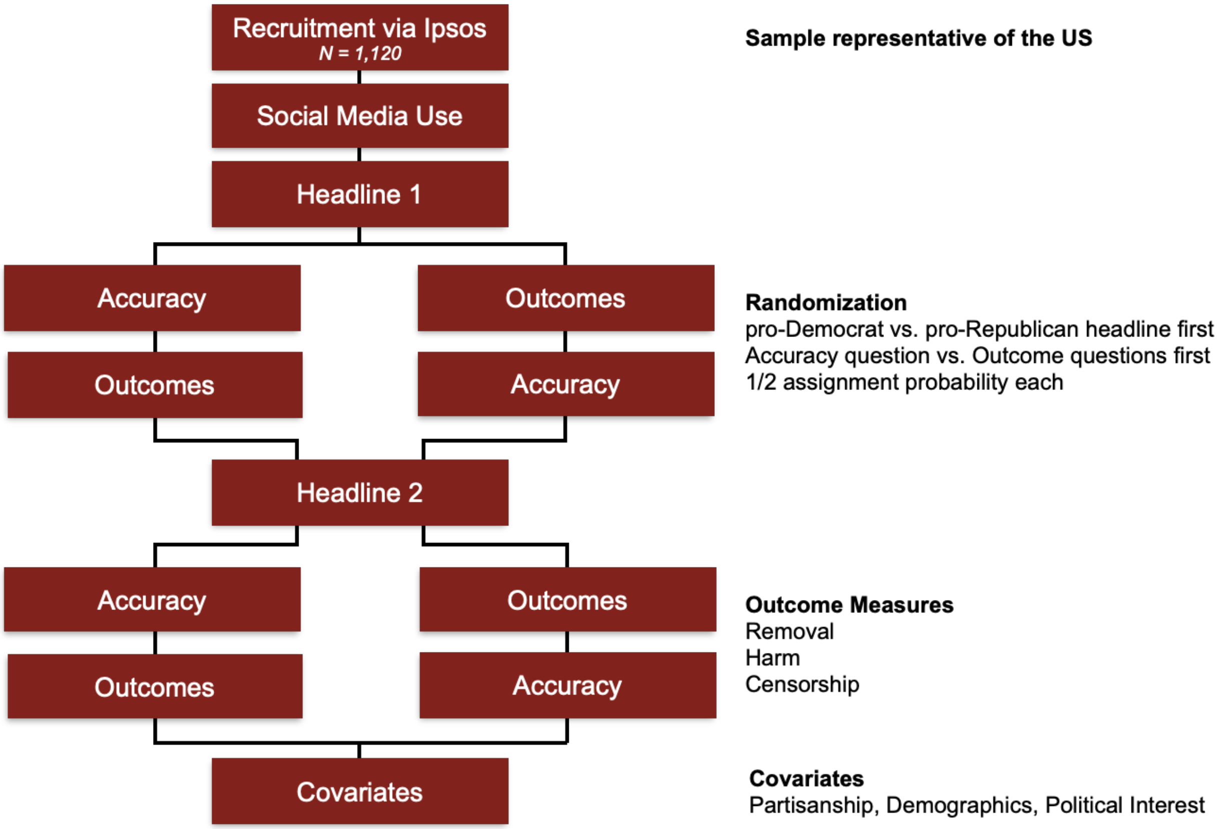

Fig. S1. Experiment design overview

S1.2 Deviations, Clarifications and Additional Analyses

Deviation #1: In the PAP, we wrote that all outcome measures are binary with the exception of the censorship measure, which was recoded as a binary measure by considering "Yes " as 1, and "No" and "Don't know" as 0. We code "Don't know" as a missing value instead of 0 because recoding "Don't know" as 0 would have imposed a strong assumption that undecided participants actually did not think of headline removal as censorship. We provide results for the main models with the original coding as a robustness check in Section S2.1.2, and find that the main results remain the same.

Deviation #2: In the PAP, we said we would estimate models for those who perceived the headline to be accurate, and separately for those who perceived the headline to be inaccurate. We deviated from this by presenting models for all participants vs. the subgroup of participants that perceived the headline as inaccurate. Since models with all participants contain both the accurate and the inaccurate subgroup and are therefore an average of the two subgroups, the difference between all observations and the inaccurate subgroup gives us insight into the accurate subgroup.

Deviation #3: For the main models including controls, we deviate from the PAP and did not constrain β_D and β_R to be the same. This is because different than in the case of (*14*), the effects for Democrats and Republicans were not similar and we did not want to risk masking true differences by analyzing a combined effect.

Deviation #4: The selection of control variables was constrained by the data that Ipsos collected. Some of the control variables we had asked for—political efficacy, affective polarization, voting behavior in 2020, political news consumption, whether a user ever used social media, whether a user had ever been banned from social media—were not implemented in the survey and hence do not appear as control variables in our analysis.

Deviation #5: One variable we had requested measuring how much participants think that political information from a range of different sources, including print media and social media, can be trusted, was not included as requested. The dataset did include a grid of trust questions that measured whether participants trusted social media companies, the news media, that the news media reported in an unbiased manner, and institutions like the government. However, those variables did not focus on political news, and were only assigned to half of respondents, perhaps because it was part of another experiment in the larger Ipsos survey. Because of the different nature of the question and the low number of responses, we could not include any control variable for trust in media.

Deviation #6: In the PAP, we said we would conduct a mediation analysis where the mediator of the effect of political alignment of the headline on censorship perception or removal is the perception of accuracy of the headline. We conduct a mediation analysis for the effect of political alignment on the outcomes intent to remove and intent to report as harmful, both of which showed significant party promotion effects, but not on perception of headline removal as censorship because there was no significant effect of party promotion for this outcome, and therefore no relationship to mediate.

Clarification #1: We remove participants for whom no survey language was indicated and participants that indicated proficiency in Spanish only.

Clarification #2: We excluded participants who had missing values for partisanship or indicated that they favored a party other than Democrats, Republicans, or Independents.

Clarification #3: We removed 243 participants who were part of a student sample that was different from the sample meant to be representative of the U.S. population.

Additional analyses: We ran additional analyses that were not pre-registered: regressions of the main outcomes considering only the first headline that participants rated, regressions without interaction effects, regressions with consensus headlines only, regressions disaggregated by headline, and regressions including a triple interaction between accuracy question order, participant partisanship, and headline alignment; a regression of perceived headline accuracy on partisanship and alignment; balance checks comparing respondents overall to the subset of respondents that agree a headline is inaccurate; mediation analyses for the effect of partisanship for all outcomes.

S1.3 Analysis

S1.3.1 Modeling

Main Models

Similar to the approach used by (*14*), we ran regression analyses with interaction terms for

partisanship of participants and political alignment of the headlines:

$$censorship_{ia} = \beta_D D_i \cdot Hd_a + \beta_R R_i \cdot Hr_a + \gamma_D \cdot D_i + \gamma_R R_i + \varepsilon_{ia} \quad \text{(S1)}$$

$$removal_{ia} = \beta_D D_i \cdot Hd_a + \beta_R R_i \cdot Hr_a + \gamma_D \cdot D_i + \gamma_R R_i + \varepsilon_{ia} \quad \text{(S2)}$$

$$harm_{ia} = \beta_D D_i \cdot Hd_a + \beta_R R_i \cdot Hr_a + \gamma_D \cdot D_i + \gamma_R R_i + \varepsilon_{ia} \quad \text{(S3)}$$

$censorship_{ia}$ is a binary measure of whether an individual i rated the removal of headline a as censorship.

$removal_{ia}$ is a binary measure of whether an individual i thinks the social media platform should remove the headline a from its platform.

$harm_{ia}$ is a binary measure of whether an individual i would report the content of the headline a as harmful to the social media platform.

D_i equals 1 when respondent i is a Democrat, and 0 otherwise.

R_i equals 1 when respondent i is a Republican, and 0 otherwise.

Hd_a equals 1 when headline a is aligned with Democratic views, and 0 otherwise.

Hr_a equals 1 when headline a is aligned with Republican views, and 0 otherwise.

β_D measures whether a Democrat is more likely to (1) perceive removal of pro-Democratic content as censorship (in equation S1), (2) think the social media platform should remove a pro-Democratic headline (in equation S2), or (3) report the content of a pro-Democratic headline as harmful to the social media platform (in equation S3).

β_R measures whether a Republican is more likely to (1) perceive removal of pro-Republican content as censorship (in equation S1), (2) think the social media platform should remove a pro-Republican headline (in equation S2), or (3) report the content of a pro-Republican headline as harmful to the social media platform (in equation S3).

Given that the headlines are balanced in terms of political alignment and randomly assigned to participants, the estimated β parameters measure the effect of political alignment.

In the pre-analysis plan, we said we would control for perceived accuracy by using a subgroup analysis to estimate the models just for those who perceived the headline to be accurate, and separately for those who perceived the headline to be inaccurate. We deviated in that we present models for all participants vs. the subgroup of participants that perceived the headline as inaccurate. This still allows us to evaluate whether the partisanship of the headline influences evaluations of whether it should be removed, whether it should be reported as harmful and whether removal would be considered censorship, among those who evaluated the accuracy of the headline in the same way because all observations contain both the accurate and the inaccurate subgroup, and are therefore an average of the two subgroups, so a difference between all observations and the inaccurate subgroup implies a difference between the two subgroups. Additionally, our main interest is in analyzing the views of the inaccurate subgroup, allowing us to evaluate how participants reacted to misinformation headlines they believed were false.

We first ran all specifications first without control variables. We also ran specifications with controls (see Section S1.6 for a list of control variables).

For the models including controls, deviating from the pre-analysis plan, we did not constrain β_D and β_R to be the same, because different than in the case of (*14*), the effects for Democrats and Republicans were not similar and we did not want to risk masking true differences by analyzing a combined effect.

The data were weighted with the weights provided by Ipsos for the models presented in the main text, but we also report unweighted results in Section S2.

We used standard errors clustered on participants for the main models, and show results with standard (i.e., non-clustered) standard errors in Section S2. We used the default clustering option in the `lm_robust` function in the `estimatr` R package to cluster standard errors on participants.

Additional Analyses

In the main text, we also present barplots showing estimates by partisanship. For these barplots, we ran slight, not pre-registered variants of the main models of the form:

$$Y_{ia} = \beta \cdot \text{partisanship}_i + \varepsilon_{ia} \tag{S4}$$

Y_{ia} is the binary outcome measure for individual i and headline a. For estimates for aligned and misaligned headlines, we subset to aligned and misaligned headlines, respectively. These models yield the same estimates as the main models for misaligned headlines, but provide estimates for aligned headlines rather than only interaction effect estimates. Models were weighted and used standard errors clustered on participants.

Also in addition to the pre-registered analyses, we ran regressions of our main outcomes considering only the first headline that participants rated, regressions without interaction effects, regressions with consensus headlines only, regressions disaggregated by headline, regressions with the subsets of participants who saw the accuracy question first or second, and regressions including a triple interaction between accuracy question order, participant partisanship, and headline alignment, and a regression of perceived headline accuracy on partisanship and alignment. We show all results that are not already shown in the main text in Section S2.

S1.4 Data

S1.4.1 Missing Data

For variables with missing data, we (1) imputed missing data using the `Amelia` package in R (*70*), and (2) used listwise deletion to remove observations with missing data. We show the results for both approaches in Section S2.

S1.4.2 Balance Checks

Here, we present balance tables of control variables across the different experiment arms (aligned vs. misaligned headlines, accuracy questions displayed before vs. after treatment).

Overall, the different experimental groups are relatively balanced. For partisan alignment (see Table S1), the Hispanic indicator has the highest Standardized Mean Difference (SMD), but the randomization seems to have been effective. For the accuracy question order (see Table S2), the control variables education, household income and whether social media is the most common news format have relatively high SMD, suggesting that it is worthwhile to include these control variables in some of our models.

We also included balance tables going beyond our pre-registration. To check if Democrats and Republicans were differentially selected based on their accuracy ratings, we provide two balance tables (one for Democrat respondents and one for Republican respondents) comparing respondents overall to the subset of respondents that agree a headline is inaccurate. We do not find evidence for

differential selection because the subsets are balanced on their observed characteristics. Note that each participant rated the accuracy of two headlines, therefore each participant accounts for up to two observations in the data.

Table S1. Balance Table for Partisan Alignment of Headline, First Headline

	Aligned			
**Variable**	**Yes**	**No**	**p-value**	**SMD**
Number of Observations	558	562		
Age (mean (SD))	53.74 (16.40)	52.84 (16.67)	0.360	0.055
Gender = Female (N (%))	307 (55.0)	324 (57.7)	0.408	0.053
Education (N (%))			0.948	0.051
... No high school diploma or GED	24 (4.3)	25 (4.4)		
... High school graduate	148 (26.5)	143 (25.4)		
... Some college or Associate degree	168 (30.1)	179 (31.9)		
... Bachelor's degree	128 (22.9)	121 (21.5)		
... Master's degree or above	90 (16.1)	94 (16.7)		
Hispanic = Yes (N (%))	85 (15.2)	63 (11.2)	0.057	0.119
Race = Non-White (N (%))	235 (42.1)	256 (45.6)	0.272	0.069
Household Income (N (%))			0.769	0.109
... Under $10,000	16 (2.9)	12 (2.1)		
... $10,000 to $24,999	41 (7.3)	41 (7.3)		
... $25,000 to $49,999	99 (17.7)	90 (16.0)		
... $50,000 to $74,999	101 (18.1)	97 (17.3)		
... $75,000 to $99,999	83 (14.9)	83 (14.8)		
... $100,000 to $149,999	97 (17.4)	119 (21.2)		
... $150,000 or more	121 (21.7)	120 (21.4)		
Political Interest (mean (SD))	2.82 (0.68)	2.82 (0.67)	0.936	0.005
Social Media Most Common News Format = Yes (N (%))	81 (14.8)	95 (17.2)	0.309	0.066
Social Media Post Flagged = Yes (N (%))	63 (14.2)	69 (15.6)	0.617	0.040
Social Media Post Removed = Yes (N (%))	57 (12.6)	62 (13.9)	0.637	0.038

Note: p-values result from a joint F-test for continuous variables and from a Chi-squared test for categorical variables. Standardized mean difference (SMD) and p-values are exactly the same for the subset of data on the second headline, only the data in the Yes and No columns would be reversed, therefore we show only one table.

Table S2. Balance Table for Accuracy Question Order

Variable	Accuracy Question Order: First	Accuracy Question Order: Second	p-value	SMD
Number of Observations	581	539		
Age (mean (SD))	53.00 (16.28)	53.59 (16.81)	0.551	0.036
Gender = Female (N (%))	331 (57.0)	300 (55.7)	0.702	0.026
Education (N (%))			0.048	0.186
... No high school diploma or GED	23 (4.0)	26 (4.8)		
... High school graduate	133 (22.9)	158 (29.3)		
... Some college or Associate degree	200 (34.4)	147 (27.3)		
... Bachelor's degree	128 (22.0)	121 (22.4)		
... Master's degree or above	97 (16.7)	87 (16.1)		
Hispanic = Yes (N (%))	77 (13.3)	71 (13.2)	1.000	0.002
Race = Non-White (N (%))	255 (43.9)	236 (43.8)	1.000	0.002
Household Income (N (%))			0.089	0.199
... Under $10,000	14 (2.4)	14 (2.6)		
... $10,000 to $24,999	34 (5.9)	48 (8.9)		
... $25,000 to $49,999	100 (17.2)	89 (16.5)		
... $50,000 to $74,999	100 (17.2)	98 (18.2)		
... $75,000 to $99,999	75 (12.9)	91 (16.9)		
... $100,000 to $149,999	125 (21.5)	91 (16.9)		
... $150,000 or more	133 (22.9)	108 (20.0)		
Political Interest (mean (SD))	2.82 (0.69)	2.83 (0.66)	0.773	0.017
Social Media Most Common News Format = Yes (N (%))	77 (13.5)	99 (18.6)	0.024	0.141
Social Media Post Flagged = Yes (N (%))	62 (13.2)	70 (16.7)	0.182	0.096
Social Media Post Removed = Yes (N (%))	66 (14.1)	53 (12.4)	0.510	0.051

Note: p-values result from a joint F-test for continuous variables and from a Chi-squared test for categorical variables. Accuracy question order was randomized at the participant level, therefore balance checks were run on the short data frame with one headline observations per participant.

Table S3. Balance Table for Subset of Democrat Respondents

Variable	Subset of Democrat Respondents		p-value	SMD
	Inaccurate Subset	All Respondents		
Number of Observations	1084	1346		
Age (mean (SD))	51.98 (16.77)	51.88 (16.83)	0.880	0.006
Gender = Female (N (%))	652 (60.1)	804 (59.7)	0.868	0.008
Education (N (%))			0.097	0.115
... No high school diploma or GED	30 (2.8)	56 (4.2)		
... High school graduate	240 (22.1)	336 (25.0)		
... Some college or Associate degree	325 (30.0)	404 (30.0)		
... Bachelor's degree	257 (23.7)	290 (21.5)		
... Master's degree or above	232 (21.4)	260 (19.3)		
Hispanic = Yes (N (%))	156 (14.4)	186 (13.8)	0.730	0.016
Race = Non-White (N (%))	630 (58.1)	812 (60.3)	0.289	0.045
Household Income (N (%))			0.721	0.079
... Under $10,000	28 (2.6)	46 (3.4)		
... $10,000 to $24,999	76 (7.0)	112 (8.3)		
... $25,000 to $49,999	192 (17.7)	234 (17.4)		
... $50,000 to $74,999	206 (19.0)	252 (18.7)		
... $75,000 to $99,999	149 (13.7)	192 (14.3)		
... $100,000 to $149,999	207 (19.1)	250 (18.6)		
... $150,000 or more	226 (20.8)	260 (19.3)		
Political Interest (mean (SD))	2.87 (0.63)	2.87 (0.65)	0.998	<0.001
Social Media Most Common News Format = Yes (N (%))	185 (17.2)	222 (16.8)	0.816	0.012
Social Media Post Flagged = Yes (N (%))	100 (11.4)	120 (11.4)	1.000	0.001
Social Media Post Removed = Yes (N (%))	74 (8.4)	100 (9.4)	0.528	0.033

Note: p-values result from a joint F-test for continuous variables and from a Chi-squared test for categorical variables. Each participant rated the accuracy of two headlines, therefore each participant may account for up to two observations in the data if they rated the accuracy of two headlines.

Table S4. Balance Table for Subset of Republican Respondents

Variable	Subset of Republican Respondents: Inaccurate Subset	Subset of Republican Respondents: All Respondents	p-value	SMD
Number of Observations	645	894		
Age (mean (SD))	55.64 (15.77)	55.41 (15.85)	0.778	0.015
Gender = Female (N (%))	330 (51.2)	458 (51.2)	1.000	0.001
Education (N (%))			0.831	0.063
... No high school diploma or GED	26 (4.0)	42 (4.7)		
... High school graduate	166 (25.7)	246 (27.5)		
... Some college or Associate degree	209 (32.4)	290 (32.4)		
... Bachelor's degree	162 (25.1)	208 (23.3)		
... Master's degree or above	82 (12.7)	108 (12.1)		
Hispanic = Yes (N (%))	80 (12.4)	110 (12.3)	1.000	0.003
Race = Non-White (N (%))	116 (18.0)	170 (19.0)	0.655	0.027
Household Income (N (%))			0.969	0.060
... Under $10,000	6 (0.9)	10 (1.1)		
... $10,000 to $24,999	32 (5.0)	52 (5.8)		
... $25,000 to $49,999	99 (15.3)	144 (16.1)		
... $50,000 to $74,999	106 (16.4)	144 (16.1)		
... $75,000 to $99,999	97 (15.0)	140 (15.7)		
... $100,000 to $149,999	141 (21.9)	182 (20.4)		
... $150,000 or more	164 (25.4)	222 (24.8)		
Political Interest (mean (SD))	2.76 (0.70)	2.76 (0.70)	0.992	0.001
Social Media Most Common News Format = Yes (N (%))	85 (13.3)	130 (14.8)	0.468	0.042
Social Media Post Flagged = Yes (N (%))	94 (17.9)	144 (19.9)	0.396	0.053
Social Media Post Removed = Yes (N (%))	90 (17.2)	138 (19.1)	0.438	0.049

Note: p-values result from a joint F-test for continuous variables and from a Chi-squared test for categorical variables. Each participant rated the accuracy of two headlines, therefore each participant may account for up to two observations in the data if they rated the accuracy of two headlines.

S1.5 Headlines

Below is the bank of false news headlines. These false news headlines are based on headlines from Snopes.com, a major established fact checking site. On May 19, 2021, we went through the 50 most recent pages of the Snopes Politics Archive (https://www.snopes.com/fact-check/category/politics/) to retrieve recent fact-checked fake news headlines.

The fact checks on these pages were published between May 18, 2021, and December 22, 2020, in the Politics category. The recency of the headlines at the time of our study ensured that they were not outdated and came from the same political period. We only considered claims that were labeled as "false" (i.e., we excluded "mixed", "mostly false", "true", and any other category). Additionally, because there were far fewer fake news headlines that were aligned for Democrats, we looked beyond the initial dates and identified a false claim on Snopes from an earlier date that still seemed relevant and was studied in a recent conference paper (*71*). We then excluded headlines that did not have a clear partisan slant (e.g., headlines with less well-known political figures or headlines that required additional context to easily understand them), were miscategorized (e.g., related to the Business category on Snopes), or were outdated. Finally, among headlines aligned for either Democrats or Republicans, we assessed the headlines in terms of the intensity of the false information (e.g., headlines involving physical violence—murder, torture, mutilation—would have greater intensity than those that deal with incompetence) and the topic they cover (e.g., racism, protest), and selected those that were balanced in terms of intensity and topic.

To maximize ecological validity, we formatted headlines in ways similar to how headlines would appear on social media. We created a template headline in the Facebook format, and then took the claims from Snopes (or the original news headline, if the primary source was a news article, cited in Snopes) as the headline text. Some headlines were slightly modified from what is shown on Snopes or the original, e.g., removing punctuation at the end of a claim. We then appended an image, either from the related Snopes article, the primary source, or a search for images related to the headline text via Google. Headline and image sources were accessed in May 2021. The original and modified headline text as well as text and image sources for the final headlines are detailed in Tables S5 and S6.

We pretested the headlines for partisan alignment and excluded one headline that was perceived as neutral rather than aligned for either party, as well as another headline that was not perceived as strongly aligned in order to rebalance the number of headlines aligned for either party. For all other headlines in the headline bank, the pretest with a convenience sample of $N = 20$ showed partisan alignment in the expected direction. The headlines were also relatively balanced in terms of the extent to which they were aligned with each party and in terms of their perceived intensity (i.e., how worrying or exciting they seemed to participants).

Pro-Democrat ***Pro-Republican***

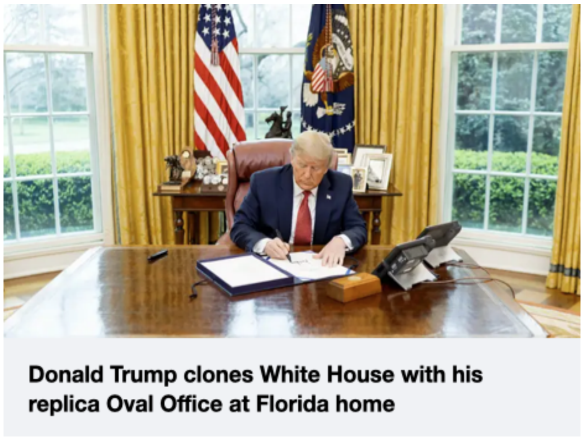

Pro-Democrat 1

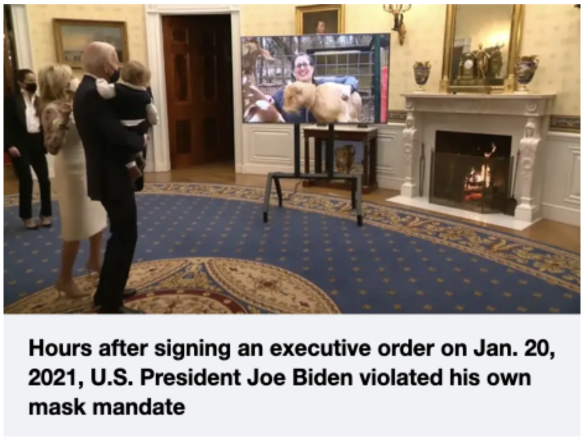

Pro-Republican 1

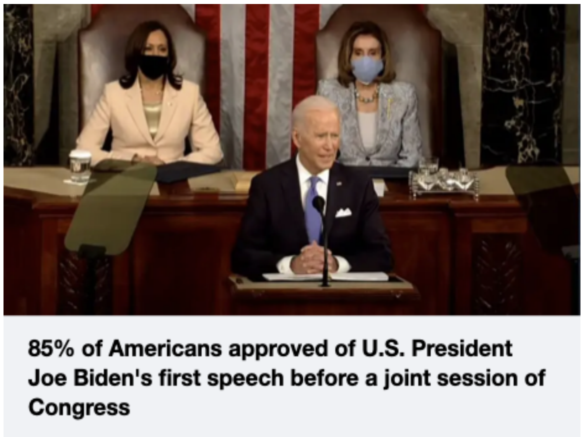

Pro-Democrat 2

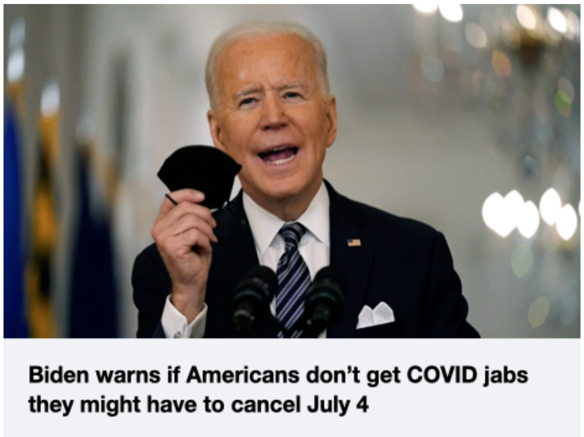

Pro-Republican 2

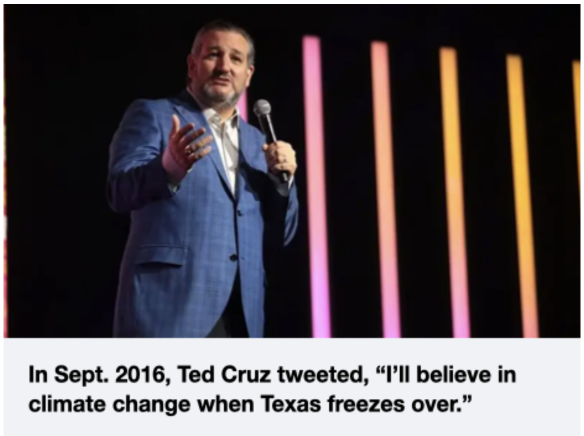

Pro-Democrat 3

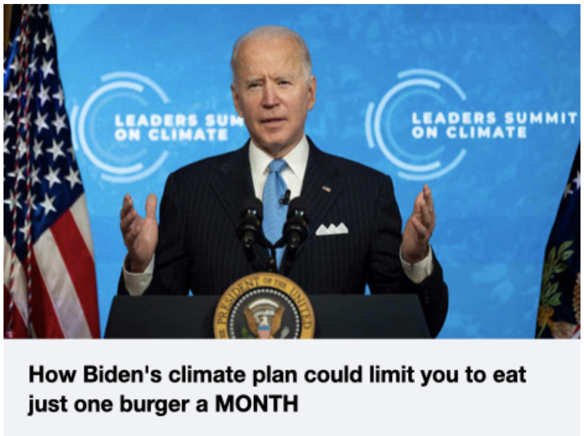

Pro-Republican 3

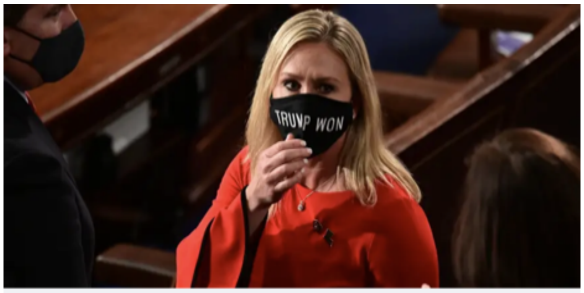

"If English was good enough for Jesus, it's good enough for us," stated Marjorie Taylor Greene, explaining why Spanish shouldn't be spoken in a Christian Nation like America

Pro-Democrat 4

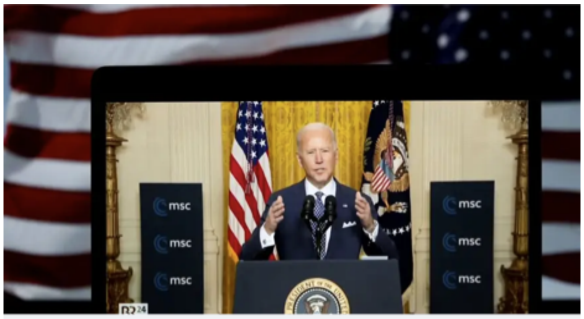

On Feb. 19, 2021, U.S. President Joe Biden uttered a racial slur referring to Black people in a recorded speech

Pro-Republican 4

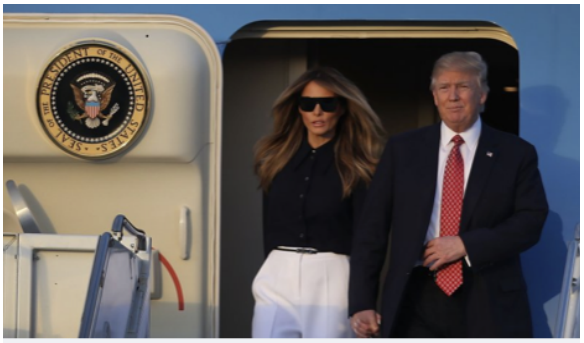

Former U.S. first lady Melania Trump had her own private bedroom on Air Force One

Pro-Democrat 5

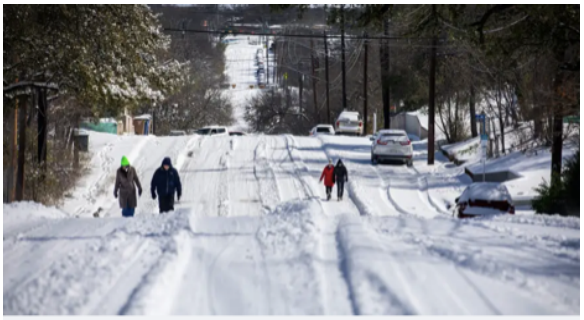

Smoking Gun! Joe Biden's Dept. of Energy Blocked Texas from Increasing Power Ahead of Killer Storm

Pro-Republican 5

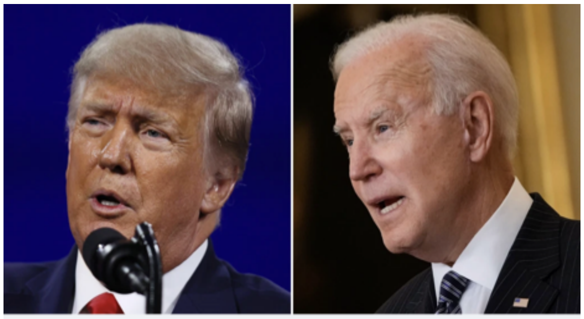

Former U.S. President Donald Trump left his successor, President Joe Biden, a letter that read, "Joe, you know I won."

Pro-Democrat 6

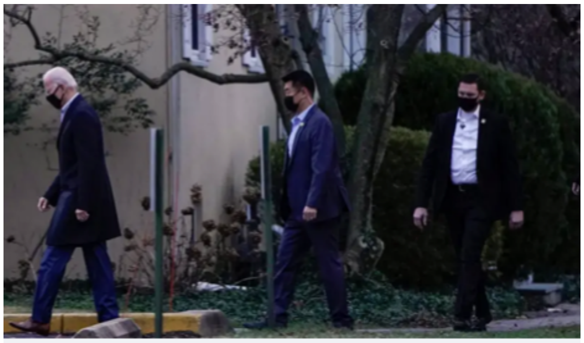

U.S. President Joe Biden's lead secret service agent is Chinese

Pro-Republican 6

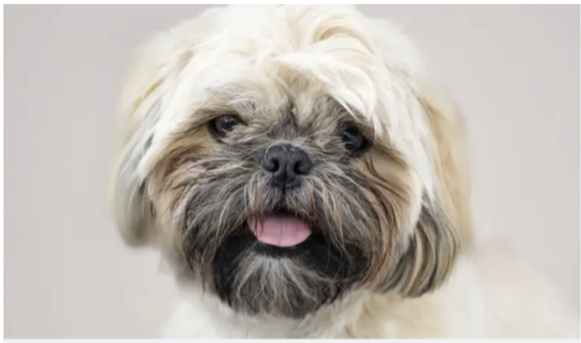

Donald Trump once evicted a disabled combat veteran for owning a small therapy dog

Pro-Democrat 7

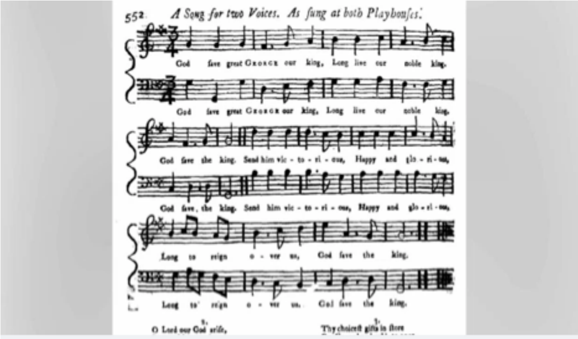

Oxford wants to scrap sheet music over complicity in 'white supremacy'

Pro-Republican 7

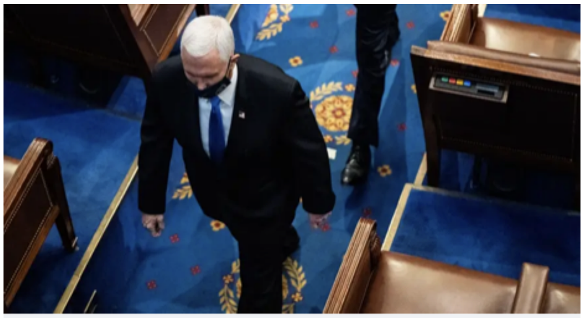

Former U.S. Vice President Mike Pence unfollowed President Donald Trump following Jan. 6, 2021, riots in Washington, D.C.

Pro-Democrat 8

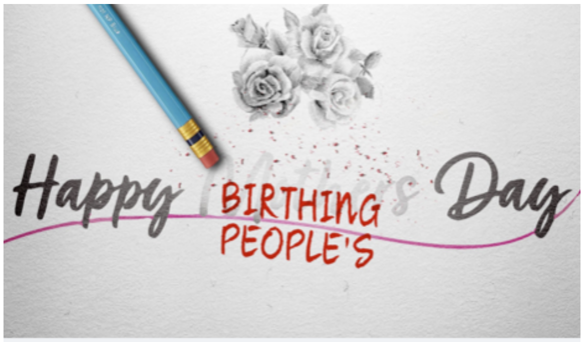

Democrats are trying to change Mother's Day to Birthing People's Day

Pro-Republican 8

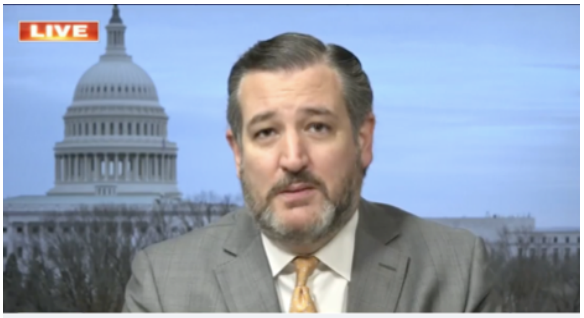

U.S. Sen. Ted Cruz messed up when he attributed the famous phrase "full of sound and fury / signifying nothing" to William Shakespeare

Pro-Democrat 9

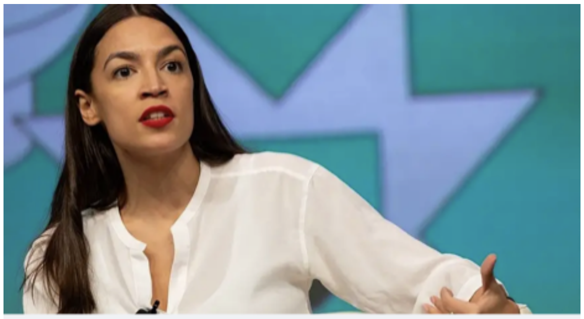

Rep. Alexandria Ocasio-Cortez said "I hope I never have twins because I can't afford to be pregnant for 18 months."

Pro-Republican 9

Table S5. Sources and Text for Pro-Democrat Headlines

Headline Source	Image Source	Original Headline	New Headline	Change
https://www.snopes.com/fact-check/replica-oval-office-mar-a-lago/	https://www.snopes.com/fact-check/replica-oval-office-mar-a-lago/	Donald Trump clones White House with his replica Oval Office at Florida home	Donald Trump clones White House with his replica Oval Office at Florida home	
https://www.snopes.com/fact-check/85-percent-americans-biden-speech/	https://www.snopes.com/fact-check/85-percent-americans-biden-speech/	85% of Americans approved of U.S. President Joe Biden's first speech before a joint session of Congress.	85% of Americans approved of U.S. President Joe Biden's first speech before a joint session of Congress	Removed .
https://www.snopes.com/fact-check/ted-cruz-texas-freezes-over/	https://www.snopes.com/fact-check/ted-cruz-fall-asleep-joe-biden/	In Sept. 2016, Ted Cruz tweeted, "I'll believe in climate change when Texas freezes over."	In Sept. 2016, Ted Cruz tweeted, "I'll believe in climate change when Texas freezes over."	
https://www.snopes.com/fact-check/marjorie-greene-jesus-english/	https://www.snopes.com/fact-check/marjorie-greene-jesus-english/	U.S. Rep. Marjorie Taylor Greene said "If English was good enough for Jesus, it's good enough for us."	"If English was good enough for Jesus, it's good enough for us," stated Marjorie Taylor Greene, explaining why Spanish shouldn't be spoken in a Christian Nation like America	Text taken from tweet instead of Snopes claim
https://www.snopes.com/fact-check/melania-trump-bedroom/	https://time.com/4668898/donald-trump-birmingham-visit-protests/	Former U.S. first lady Melania Trump had her own private bedroom on Air Force One.	Former U.S. first lady Melania Trump had her own private bedroom on Air Force One	Removed .
https://www.snopes.com/fact-check/joe-you-know-i-won-letter-trump/	https://nypost.com/2021/03/22/trump-says-he-wrote-biden-a-letter-despite-china-ties/	Former U.S. President Donald Trump left his successor, President Joe Biden, a letter that read, "Joe, you know I won."	Former U.S. President Donald Trump left his successor, President Joe Biden, a letter that read, "Joe, you know I won."	
https://www.snopes.com/fact-check/trump-veteran-service-dog/	https://www.snopes.com/fact-check/trump-veteran-service-dog/	Donald Trump once evicted a disabled combat veteran for owning a small therapy dog.	Donald Trump once evicted a disabled combat veteran for owning a small therapy dog.	Removed .
https://www.snopes.com/fact-check/pence-trump-twitter-jan-6-riots/	https://www.snopes.com/fact-check/pence-trump-twitter-jan-6-riots/	U.S. Vice President Mike Pence unfollowed President Donald Trump following Jan. 6, 2021, riots in Washington, D.C.	U.S. Vice President Mike Pence unfollowed President Donald Trump following Jan. 6, 2021, riots in Washington, D.C.	
https://www.snopes.com/fact-check/ted-cruz-sound-fury-quote/	https://video.foxnews.com/v/6230815030001#sp=show-clips (screen capture from video "Ted Cruz: Impeachment trial will 'not succeed,' Trump will be acquitted)	U.S. Sen. Ted Cruz messed up when he attributed the famous phrase "full of sound and fury / signifying nothing" to William Shakespeare.	U.S. Sen. Ted Cruz messed up when he attributed the famous phrase "full of sound and fury / signifying nothing" to William Shakespeare	Removed .

Table S6. Sources and Text for Pro-Republican Headlines

**Headline Source**	**Image Source**	**Original Headline**	**New Headline**	**Change**
https://www.snopes.com/fact-check/biden-violate-mask-mandate/	https://www.snopes.com/fact-check/biden-violate-mask-mandate/	Hours after signing an executive order on Jan. 20, 2021, U.S. President Joe Biden violated his own mask mandate.	Hours after signing an executive order on Jan. 20, 2021, U.S. President Joe Biden violated his own mask mandate	Removed .
https://www.snopes.com/fact-check/biden-cancel-fourth-of-july/	https://nypost.com/2021/04/21/biden-warns-if-americans-dont-get-covid-vaccine-they-might-have-to-cancel-july-4/	Biden warns if Americans don't get COVID jabs they might have to cancel July 4	Biden warns if Americans don't get COVID jabs they might have to cancel July 4	
https://www.snopes.com/fact-check/90-percent-red-meat/	https://www.cnbc.com/2021/05/04/biden-business-allies-help-white-house-woo-private-sector-in-climate-change-push.html	How Biden's climate plan could limit you to eat just one burger a MONTH, cost \$3.5K a year per person in taxes, force you to spend \$55K on an electric car and 'crush' American jobs	How Biden's climate plan could limit you to eat just one burger a MONTH	Removed last part
https://www.snopes.com/fact-check/joe-biden-n-word-recorded-speech/	https://www.snopes.com/fact-check/joe-biden-n-word-recorded-speech/	On Feb. 19, 2021, U.S. President Joe Biden uttered a racial slur referring to Black people in a recorded speech.	On Feb. 19, 2021, U.S. President Joe Biden uttered a racial slur referring to Black people in a recorded speech	Removed .
https://www.snopes.com/fact-check/biden-department-of-energy-block-texas-power/	https://www.snopes.com/fact-check/biden-department-of-energy-block-texas-power/	Smoking Gun! Joe Biden's Dept. of Energy Blocked Texas from Increasing Power Ahead of Killer Storm	Smoking Gun! Joe Biden's Dept. of Energy Blocked Texas from Increasing Power Ahead of Killer Storm	
https://www.snopes.com/fact-check/biden-chinese-bodyguard/	https://www.snopes.com/fact-check/biden-chinese-bodyguard/	U.S. President Joe Biden's lead secret service agent is Chinese.	U.S. President Joe Biden's lead secret service agent is Chinese	Removed .
https://www.snopes.com/fact-check/oxford-sheet-music/	https://nypost.com/2021/03/30/oxford-wants-to-ban-sheet-music-over-complicity-in-white-supremacy/	Oxford wants to scrap sheet music over complicity in 'white supremacy'	Oxford wants to scrap sheet music over complicity in 'white supremacy'	
https://www.snopes.com/fact-check/birthing-peoples-day/	https://twitter.com/tedcruz/status/1391362317246504961/photo/1	Democrats are trying to change Mother's Day to Birthing People's Day.	Democrats are trying to change Mother's Day to Birthing People's Day	Removed .
https://www.snopes.com/fact-check/aoc-twins-18-months/	https://www.snopes.com/fact-check/aoc-twins-18-months/	Rep. Alexandria Ocasio-Cortez said "I hope I never have twins because I can't afford to be pregnant for 18 months."	Rep. Alexandria Ocasio-Cortez said "I hope I never have twins because I can't afford to be pregnant for 18 months."	

S1.6 Questionnaire and Measures

Randomization and branching logic instructions provided to Ipsos are shown in italics.
CHOICE DISPLAY AND CONFIGURATION INSTRUCTIONS PROVIDED TO IPSOS ARE SHOWN IN SMALL CAPS.
[Numeric coding or transformation for variables as in the final dataset and related information about the inclusion of variables is shown surrounded by brackets.]
Variable names as they appear in the regression tables and plots are shown in bold font, but were not shown to participants.
Headers are displayed in bold and italics and are for orientation only and were not displayed to participants.

Question numbering was not displayed to participants.

Survey experiment measures are listed first and control variables second. We had asked Ipsos to measure the control first (i.e., pre-treatment). Additionally, for some questions, the exact question wording was not provided since they were part of the standard demographics or sample variables.

S1.6.1 Survey Experiment Measures

1. ***Headline display***
   We have a bank of 9 pro-Democrat (headline_pro_dem_[number].png) and 9 pro-Republican (headline_pro_rep_[number].png) headline images. Each respondent should be shown 1 pro-Democrat image and 1 pro-Republican image. Whether the respondent sees a pro-Democrat or pro-Republican image first should be randomized

2. ***Headline 1***

   1. Someone has shared the following headline on a social media site. (This headline has been established as **false** by third party fact checkers.)
      Randomly select 1 (pro_rep/pro_dem) image and insert image file here
      THE IMAGE SHOULD REMAIN VISIBLE FOR THE CENSORSHIP, ACCURACY, AND HARM QUESTIONS THAT FOLLOW

3. ***Measuring outcomes 1***
   Randomize question order of Censorship 1 and Accuracy 1: for half of respondents, the two "Censorship 1" questions should be shown first, for the other half, the "Accuracy 1" question should be shown first

4. ***Censorship 1***

   1. **Intent to Remove Headline:** How do you think the social media company should handle this headline?
      SINGLE ANSWER ALLOWED

1. Allow it to remain on the social media site [0]
2. Remove it from the social media site [1]

2. **Perception of Headline Removal as Censorship:** Imagine that the social media platform removed this headline. Would you use the word "censorship" to describe this action?
   SINGLE ANSWER ALLOWED

   1. Yes [1]
   2. No [0]
   3. Don't know [NA]

5. ***Accuracy 1***

   1. **Accuracy:** To the best of your knowledge, how accurate is the claim in the above headline?
      SINGLE ANSWER ALLOWED

      1. Not at all accurate [1]
      2. Not very accurate [2]
      3. Somewhat accurate [3]
      4. Very accurate [4]

6. ***Harm 1***

   1. **Intent to Report Headline as Harmful:** Some social media platforms allow users to report content as harmful. If you have the option of anonymously reporting this content as harmful, would you click the "report as harmful content" button for the above headline?
      SINGLE ANSWER ALLOWED

      1. Yes [1]
      2. No [0]

7. ***Headline 2***

   1. Someone has shared the following headline on a social media site. (This headline has been established as **false** by third party fact checkers.)
      Randomly select 1 (pro_rep/pro_dem) image and insert image file here
      THE IMAGE SHOULD REMAIN VISIBLE FOR THE CENSORSHIP, ACCURACY, AND HARM QUESTIONS THAT FOLLOW

8. ***Measuring outcomes 2***
   Display censorship and accuracy questions for headline 2 in the same order as those for headline 1: If participants saw "Censorship 1" before "Accuracy 1", they should see "Censorship 2" before "Accuracy 2"; if participants saw "Accuracy 1" before "Censorship 1", they should see "Accuracy 2" before "Censorship 2"

9. ***Censorship 2***

   1. **Intent to Remove Headline:** How do you think the social media company should handle this headline?
      SINGLE ANSWER ALLOWED
      1. Allow it to remain on the social media site [0]
      2. Remove it from the social media site [1]
   2. **Perception of Headline Removal as Censorship:** Imagine that the social media platform removed this headline. Would you use the word "censorship" to describe this action?
      SINGLE ANSWER ALLOWED
      1. Yes [1]
      2. No [0]
      3. Don't know [NA]

10. ***Accuracy 2***

    1. **Accuracy:** To the best of your knowledge, how accurate is the claim in the above headline?
       SINGLE ANSWER ALLOWED
       1. Not at all accurate [1]
       2. Not very accurate [2]
       3. Somewhat accurate [3]
       4. Very accurate [4]

11. ***Harm 2***

    1. **Intent to Report Headline as Harmful:** Some social media platforms allow users to report content as harmful. If you have the option of anonymously reporting this content as harmful, would you click the "report as harmful content" button for the above headline?
       SINGLE ANSWER ALLOWED
       1. Yes [1]
       2. No [0]

S1.6.2 Control Variables

The selection of control variables was constrained by the data that Ipsos collected. Some of the variables we had asked for—political efficacy, affective polarization, voting behavior in 2020, political news consumption, whether a user ever used social media, whether a user had ever been banned from social media—were not implemented in the survey and hence do not appear in the final survey data here. Other variables, like partisanship and political interest, were worded differently from those in our pre-analysis plan. Some variables that we had not originally requested,

such as whether a participant's social media posts had been flagged in the past or what their most common news source was, were used as proxies for variables that were not provided. As mentioned in our pre-analysis plan, we rely on the measures that Ipsos actually provided. The demographic variables were not listed explicitly in the pre-analysis plan because they are part of the general demographic information about a sample that Ipsos provides and we mentioned we assumed these will already be included. Here, we provide the final version of all variables that we use for analysis. One variable we had requested measuring how much participants think that political information from a range of different sources, including print media and social media, can be trusted, was not included as requested. The dataset did include a grid of trust questions that measured whether participants trusted social media companies, the news media, that the news media reported in an unbiased manner, and institutions like the government. However, those variables did not focus on political news, and the questions related to trust in media seemed to be part of another experiment in the larger Ipsos survey because only half of participants answered the question on trust in the news media, while the other half answered the question on the unbiasedness of news media reporting. Because of the different nature of the question and the low number of participants having responded to it, we did not include any control variable for trust in media.

13. ***Partisanship***

1. Generally speaking, do you think of yourself as...
   Select one answer only.
   SINGLE ANSWER ALLOWED

   1. **Republican:** Republican [Republican]
   2. **Democrat:** Democrat [Democrat]
   3. Independent [NA; excluded from anlysis]
   4. Something else [NA; excluded from analysis]

14. ***Social media use***

1. **Social Media Post Removed:** Have you ever experienced the following? - Had a social media post removed by the social media company.
   SINGLE ANSWER ALLOWED

   1. Yes, I have experienced or done this [1]
   2. No, I have not experienced or done this [0]
   3. Not applicable [NA]

2. **Social Media Post Flagged:** Have you ever experienced the following? - Had a social media post flagged, reported, or tagged with a warning label.
   SINGLE ANSWER ALLOWED [We had originally requested a variable asking whether a participant was ever banned from a social media platform, which was not included in the final survey. We therefore included this variable as a proxy for past experiences with social media content moderation.]

   1. Yes, I have experienced or done this [1]
   2. No, I have not experienced or done this [0]

3. Not applicable [NA]

3. **Social Media Most Common News Format:** In which format do you get most of your news?
SINGLE ANSWER ALLOWED
[We had originally requested a variable asking whether a participant ever uses social media. This variable was not included in the final survey. Instead, we included another measure of social media use that we had not originally requested, but was part of the data: whether social media was a participant's most common news source, which was derived recoding a variable asking respondents for the most common news source.]

   1. From a printed newspaper or magazine [0]
   2. From television [0]
   3. From radio [0]
   4. From social media [1]
   5. From friends and family [0]

15. ***Political interest***

1. **Political Interest:** How closely do you follow each of these different news topics?
GRID: NEWS ABOUT NATIONAL ISSUES AND POLITICS, NEWS ABOUT YOUR STATE GOVERNMENT, NEWS ABOUT ISSUES AFFECTING YOUR LOCAL COMMUNITY, INTERNATIONAL AFFAIRS
SINGLE ANSWER ALLOWED FOR EACH SOURCE IN THE GRID
[We had originally requested a single variable asking participants how often they pay attention to what is going on in government and politics. Since Ipsos provided a grid of related variables, we calculated an index using the average of these variables. We performed a factor analysis and found that all individual variables load onto the same factor.]

   1. Very closely [4]
   2. Somewhat closely [3]
   3. Not too closely [2]
   4. Not at all closely [1]

16. ***Demographics***

1. **Age:** Age
SINGLE ANSWER ALLOWED

2. **Gender:** Gender
SINGLE ANSWER ALLOWED

   1. Male [0]
   2. Female [1]

3. **Education:** Education
SINGLE ANSWER ALLOWED

    1. No high school diploma or GED [1]
    2. High school graduate (high school diploma or the equivalent GED) [2]
    3. Some college or Associate degree [3]
    4. Bachelor's degree [4]
    5. Master's degree or above [5]

4. **Household Income:** Household Income
SINGLE ANSWER ALLOWED

    1. Under $10,000 [1]
    2. $10,000 to $24,999 [2]
    3. $25,000 to $49,999 [3]
    4. $50,000 to $74,999 [4]
    5. $75,000 to $99,999 [5]
    6. $100,000 to $149,999 [6]
    7. $150,000 or more [7]

5. **Hispanic:** Hispanic Origin
SINGLE ANSWER ALLOWED
[Mexican/Mexican-American/Chicano; Puerto Rican; Cuban, Cuban-American; Other Spanish/Hispanic/Latino were recoded as Yes]

    1. Yes [1]
    2. No [0]

6. **Race:** Race
SINGLE ANSWER ALLOWED
[Black or African American, American Indian or Alaska Native, Asian, Native Hawaiian/Pacific Islander, 2+ races were recoded as Non-White]

    1. White [0]
    2. Non-White [1]

S1.6.3 Derived Variables and Variables Based on Stimuli

17. **Pro-Democrat Headline:** Indicates whether a headline is pro-Democrat, either by making Democrats look good or by making Republicans look bad. [1 if true, 0 otherwise]

18. **Pro-Republican Headline:** Indicates whether a headline is pro-Republican, either by making Republicans look good or by making Democrats look bad. [1 if true, 0 otherwise]

19. **Aligned:** Indicates whether a participant's partisanship and headline orientation are aligned (i.e., Democrat partisanship and pro-Democrat headline, or Republican partisanship and pro-Republican headline). [1 if true, 0 otherwise]

20. **Accuracy Binary:** Divides participants into two subgroups for each headline they see, one subgroup that considers the misinformation headline as accurate, one that considers the misinformation headline as inaccurate. [1 if the rating on Accuracy was "Somewhat accurate" or "Very accurate", 0 if rating on Accuracy was "Not at all accurate" or "Not very accurate"]

21. **Accuracy Order:** Order in which Accuracy question appeared. [1 if Accuracy questions came first (before Censorship outcome questions), 0 if Accuracy questions came second (after Censorship outcome questions)]

S1.7 Descriptive Statistics

Table S7. Descriptive Statistics

Variable	N	Mean	SD	Min	Q1	Median	Q3	Max
Age	1120	53.288	16.534	18	40	55	66	94
Gender	1120							
... Male	489	43.7%						
... Female	631	56.3%						
Education	1120							
... No high school diploma or GED	49	4.4%						
... High school graduate	291	26%						
... Some college or Associate degree	347	31%						
... Bachelor's degree	249	22.2%						
... Master's degree or above	184	16.4%						
Hispanic	1120							
... Yes	148	13.2%						
... No	972	86.8%						
Race	1120							
... White	629	56.2%						
... Non-White	491	43.8%						
Household Income	1120							
... Under $10,000	28	2.5%						
... $10,000 to $24,999	82	7.3%						
... $25,000 to $49,999	189	16.9%						
... $50,000 to $74,999	198	17.7%						
... $75,000 to $99,999	166	14.8%						
... $100,000 to $149,999	216	19.3%						
... $150,000 or more	241	21.5%						
Political Interest	1101	2.822	0.675	1	2.5	3	3.25	4
Social Media Most Common News Format	1102							
... Yes	176	16%						
... No	926	84%						
Social Media Post Flagged	888							
... Yes	132	14.9%						
... No	756	85.1%						
Social Media Post Removed	896							
... Yes	119	13.3%						
... No	777	86.7%						
Partisanship	1120							
... Democrat	673	60.1%						
... Republican	447	39.9%						

Table S8. Descriptive Statistics on Representativeness of Sample

Sample	Weighting	Median Age	Share Hispanic or Latino
US Population	unweighted	38.2	0.187
Ipsos full sample (including students)	unweighted	48.0	0.174
Ipsos full sample (including students)	weighted	40.5	0.166
Ipsos full sample (excluding students)	unweighted	54.0	0.171
Ipsos full sample (excluding students)	weighted	48.5	0.166
Ipsos sample for this study (excluding students)	unweighted	54.0	0.132
Ipsos sample for this study (excluding students)	weighted	49.5	0.126
Final sample	unweighted	55.0	0.132
Final sample	weighted	50.5	0.123

Sources: For 2020 age data: U.S. Census Bureau, 2016-2020 American Community Survey 5-Year Estimates, retrieved on September 7, 2022 from https://data.census.gov/cedsci/table?q=median%20age&g=0100000US&tid=ACSST5Y2020.S0101. For 2020 ethnicity data: U.S. Census Bureau, 2020 Census Redistricting Data (Public Law 94-171), retrieved on September 7, 2022 from https://data.census.gov/cedsci/table?q=hispanic&g=0100000US&tid=DECENNIALPL2020.P2.

S2 Additional Results

S2.1 Regression Tables and Plots

We show results in seven sections: The first section shows the main regression models, presented in one table for models shown in the main text and one figure showing various robustness checks for each outcome. The second section shows robustness checks for the main regression models where the censorship outcome is coded as pre-registered and not as mentioned in Section S1.2. The third section shows a robustness check for the main regression models including only the first headline that participants rated in the regressions. The fourth section shows regressions similar to the main regressions, but without interaction effects. The fifth section shows the main regression models when restricting the headlines to the consensus headlines only. The sixth section shows the regression results when disaggregating the models by headline. The seventh section shows models with the subsets of participants who saw the accuracy question first or second, and models with a triple interaction between accuracy question order, participant partisanship, and headline alignment. As mentioned in Section S1.2, the analyses shown in the third through the seventh section were not pre-registered.

S2.1.1 Main Models Considering All Headlines

Intent to Remove Headline

Table S9. Regression of Intent to Remove Headline on Partisanship and Alignment

	DV: Intent to Remove Headline			
	All		Inaccurate Subgroup	
	Baseline	Controls	Baseline	Controls
Democrat	0.75^{***}	0.79^{***}	0.79^{***}	0.98^{***}
	(0.02)	(0.12)	(0.02)	(0.13)
Republican	0.34^{***}	0.37^{**}	0.40^{***}	0.60^{***}
	(0.03)	(0.12)	(0.03)	(0.14)
Democrat x Pro-Democrat Headline	-0.11^{***}	-0.12^{***}	-0.07^{***}	-0.07^{**}
	(0.02)	(0.03)	(0.02)	(0.02)
Republican x Pro-Republican Headline	0.00	0.01	0.03	0.03
	(0.02)	(0.03)	(0.03)	(0.03)
Age		0.00		0.00
		(0.00)		(0.00)
Gender: Female		0.01		0.00
		(0.03)		(0.04)
Education		-0.01		$-0.04^{\cdot}$
		(0.02)		(0.02)
Hispanic		0.07		$0.09^{\cdot}$
		(0.05)		(0.05)
Race: Non-White		-0.01		0.03
		(0.04)		(0.04)
Household Income		-0.00		-0.01
		(0.01)		(0.01)
Political Interest		-0.02		-0.02
		(0.03)		(0.03)
Social Media Most Common News Format		0.03		0.01
		(0.05)		(0.06)
Social Media Post Flagged		0.01		0.03
		(0.07)		(0.09)
Social Media Post Removed		-0.15^{*}		-0.21^{*}
		(0.07)		(0.09)
R^2	0.58	0.59	0.65	0.67
Adj. R^2	0.58	0.59	0.65	0.67
Num. obs.	2190	1691	1721	1349
RMSE	0.46	0.46	0.45	0.44
N Clusters	1104	849	1003	783

$^{***}p < 0.001$; $^{**}p < 0.01$; $^{*}p < 0.05$; $^{\cdot}p < 0.1$

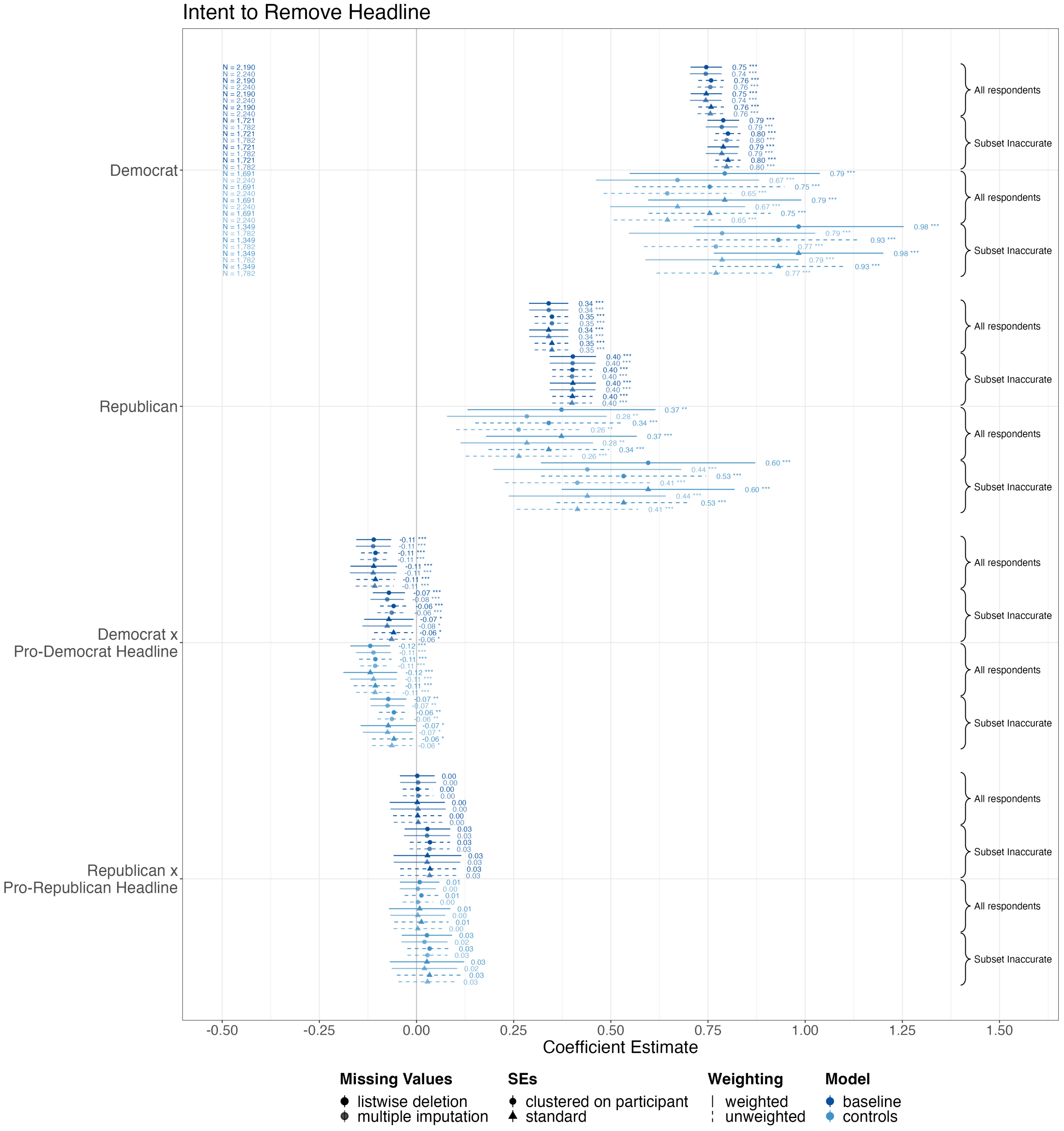

Fig. S3. Robustness checks for models with all headlines. $^{***}p < 0.001$; $^{**}p < 0.01$; $^{*}p < 0.05$.

Intent to Report Headline as Harmful

Table S10. Regression of Intent to Report Headline as Harmful on Partisanship and Alignment

	DV: Intent to Report Headline as Harmful			
	All		Inaccurate Subgroup	
	Baseline	Controls	Baseline	Controls
Democrat	0.56^{***}	0.55^{***}	0.57^{***}	0.58^{***}
	(0.02)	(0.10)	(0.02)	(0.12)
Republican	0.25^{***}	0.26^{**}	0.30^{***}	0.32^{**}
	(0.02)	(0.09)	(0.03)	(0.11)
Democrat x Pro-Democrat Headline	-0.13^{***}	-0.11^{***}	-0.11^{***}	-0.08^{*}
	(0.03)	(0.03)	(0.03)	(0.03)
Republican x Pro-Republican Headline	0.03	0.04	0.02	0.02
	(0.03)	(0.03)	(0.03)	(0.04)
Age		0.00^{**}		0.00^{**}
		(0.00)		(0.00)
Gender: Female		$-0.06^{\cdot}$		-0.05
		(0.03)		(0.04)
Education		-0.01		-0.02
		(0.02)		(0.02)
Hispanic		-0.01		0.04
		(0.05)		(0.05)
Race: Non-White		0.04		0.04
		(0.04)		(0.04)
Household Income		-0.00		-0.01
		(0.01)		(0.01)
Political Interest		-0.04		-0.02
		(0.02)		(0.03)
Social Media Most Common News Format		0.04		0.04
		(0.04)		(0.05)
Social Media Post Flagged		-0.03		-0.03
		(0.05)		(0.06)
Social Media Post Removed		$-0.10^{\cdot}$		$-0.12^{\cdot}$
		(0.05)		(0.07)
R^2	0.42	0.42	0.46	0.46
Adj. R^2	0.42	0.41	0.46	0.45
Num. obs.	2192	1692	1720	1347
RMSE	0.47	0.46	0.47	0.47
N Clusters	1105	851	1005	785

$^{***}p < 0.001$; $^{**}p < 0.01$; $^{*}p < 0.05$; $^{\cdot}p < 0.1$

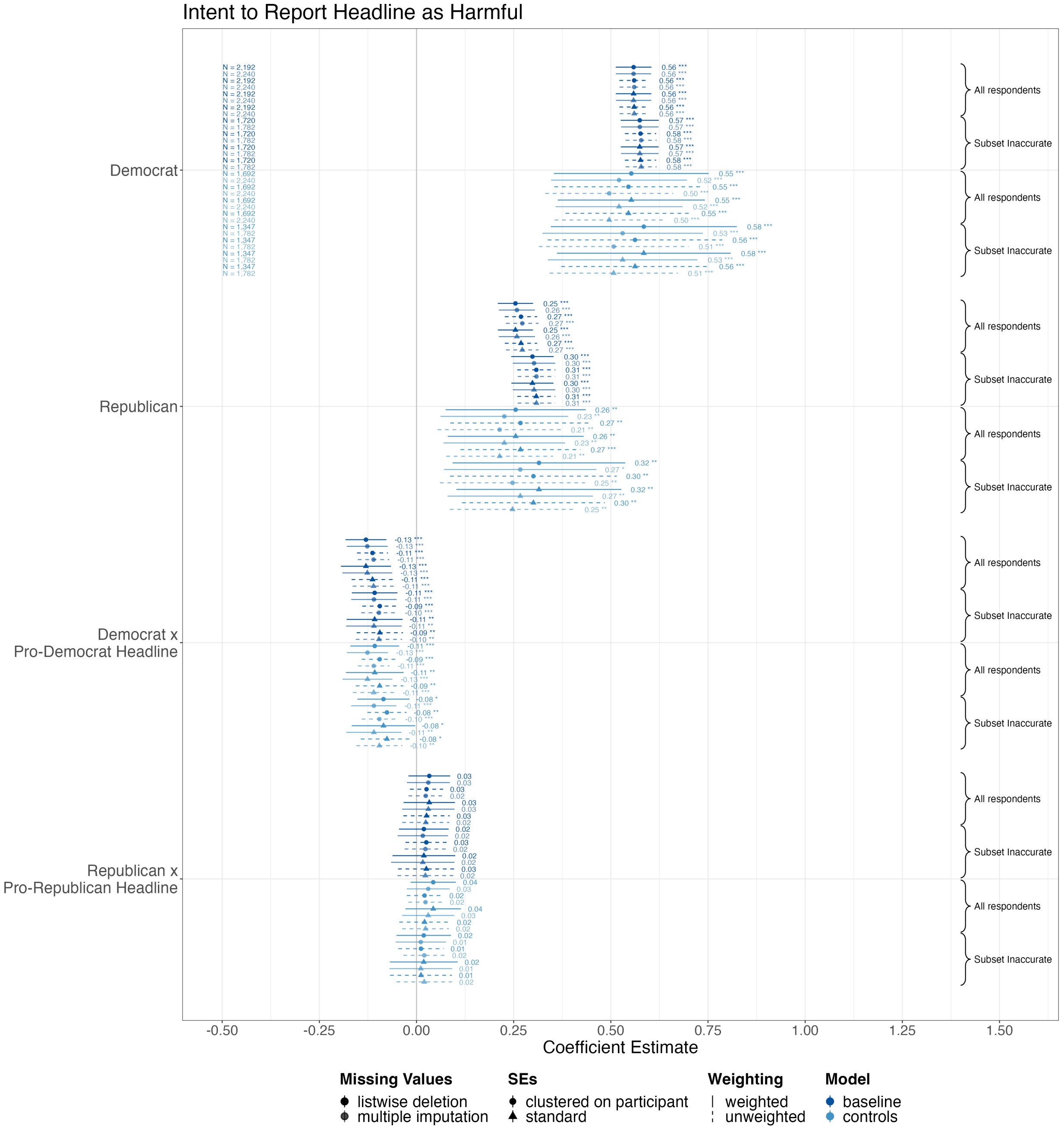

Fig. S4. Robustness checks for models with all headlines. $^{***}p < 0.001$; $^{**}p < 0.01$; $^{*}p < 0.05$.

Perception of Headline Removal as Censorship

Table S11. Regression of Perception of Headline Removal as Censorship on Partisanship and Alignment

	DV: Perception of Headline Removal as Censorship			
	All		Inaccurate Subgroup	
	Baseline	Controls	Baseline	Controls
Democrat	0.28***	0.12	0.25***	0.07
	(0.02)	(0.15)	(0.03)	(0.17)
Republican	0.65***	0.51**	0.60***	0.45**
	(0.03)	(0.14)	(0.03)	(0.16)
Democrat x Pro-Democrat Headline	0.01	0.03	0.01	0.03
	(0.02)	(0.02)	(0.02)	(0.02)
Republican x Pro-Republican Headline	−0.00	0.01	−0.04	−0.03
	(0.03)	(0.03)	(0.03)	(0.04)
Age		0.00		0.00
		(0.00)		(0.00)
Gender: Female		−0.01		0.02
		(0.04)		(0.04)
Education		−0.00		0.01
		(0.02)		(0.02)
Hispanic		−0.10·		−0.06
		(0.06)		(0.06)
Race: Non-White		0.04		0.04
		(0.04)		(0.05)
Household Income		0.00		−0.00
		(0.01)		(0.01)
Political Interest		0.03		0.03
		(0.03)		(0.04)
Social Media Most Common News Format		−0.01		−0.01
		(0.06)		(0.06)
Social Media Post Flagged		−0.08		−0.11
		(0.06)		(0.08)
Social Media Post Removed		0.19**		0.22*
		(0.06)		(0.08)
R^2	0.53	0.55	0.47	0.49
Adj. R^2	0.53	0.55	0.47	0.48
Num. obs.	1774	1406	1407	1125
RMSE	0.46	0.46	0.45	0.45
N Clusters	958	753	851	677

$^{***}p < 0.001$; $^{**}p < 0.01$; $^{*}p < 0.05$; $^{\cdot}p < 0.1$

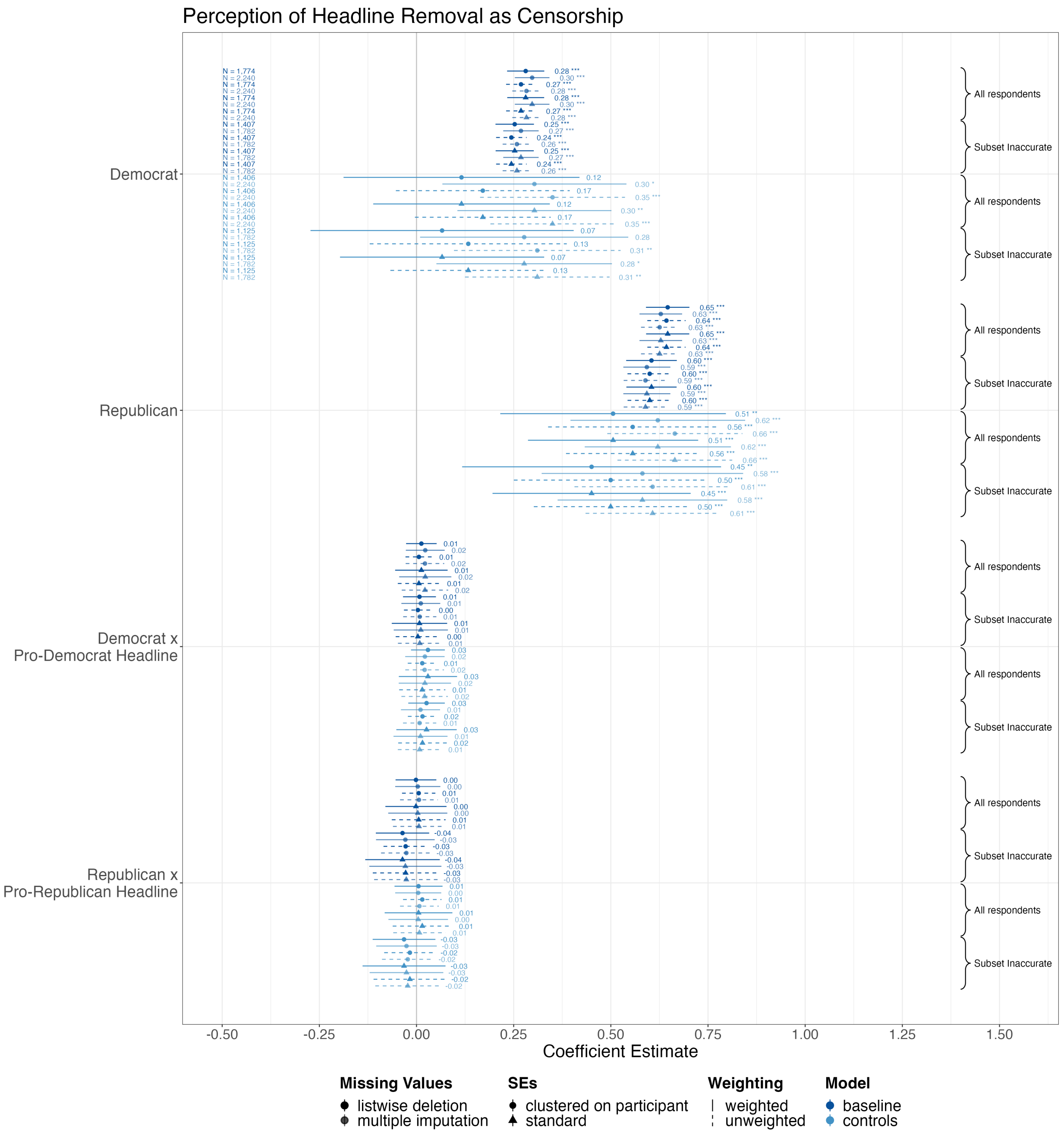

Fig. S5. Robustness checks for models with all headlines. $^{***}p < 0.001$; $^{**}p < 0.01$; $^{*}p < 0.05$.

S2.1.2 Main Models With All Headlines and Pre-Registered Censorship Coding

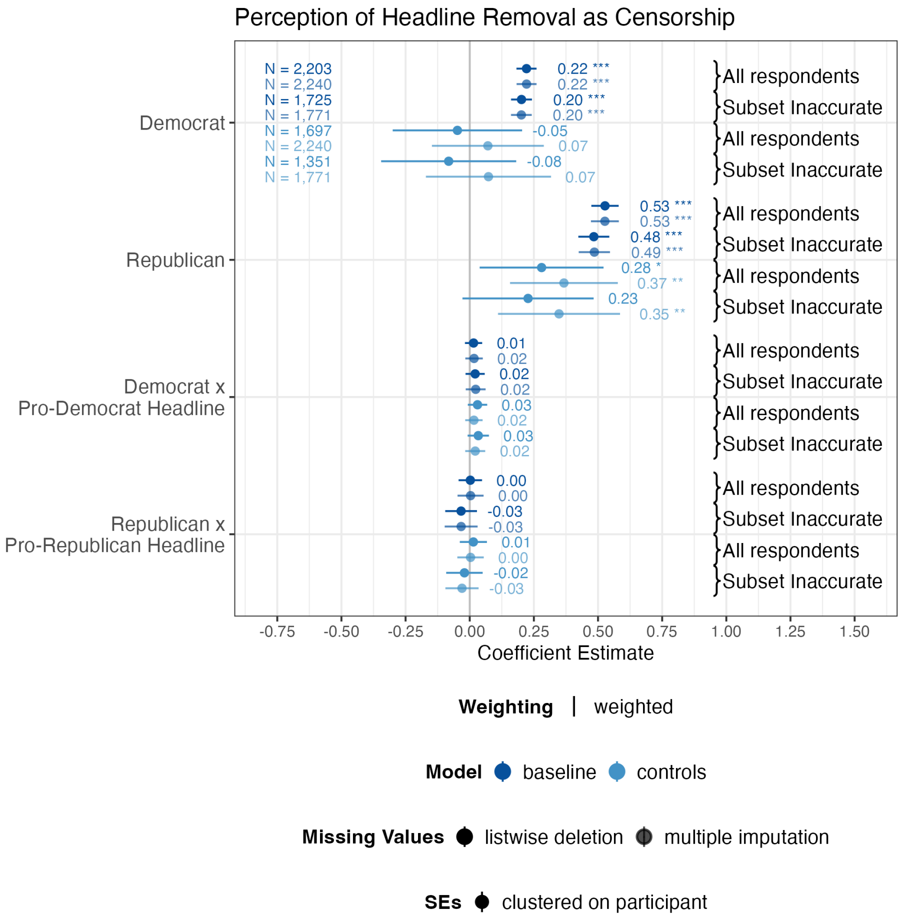

Fig. S6. Robustness checks for models with all headlines using the pre-registered censorship coding. $^{***}p < 0.001$; $^{**}p < 0.01$; $^{*}p < 0.05$.

S2.1.3 Main Models Considering Only the First Headline

Intent to Remove Headline

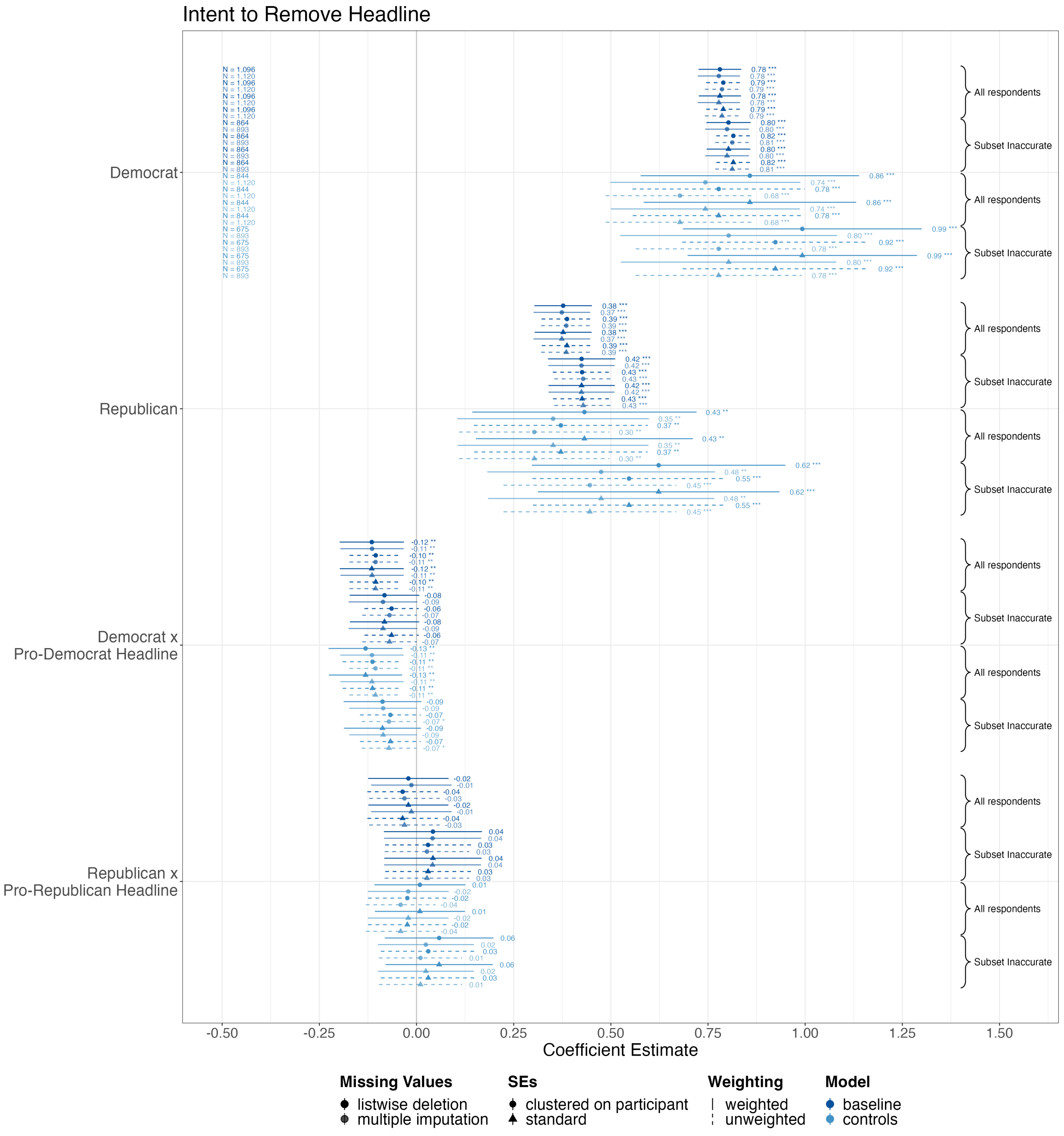

Fig. S7. Robustness checks for models with the first headline only. $^{***}p < 0.001$; $^{**}p < 0.01$; $^{*}p < 0.05$.

Intent to Report Headline as Harmful

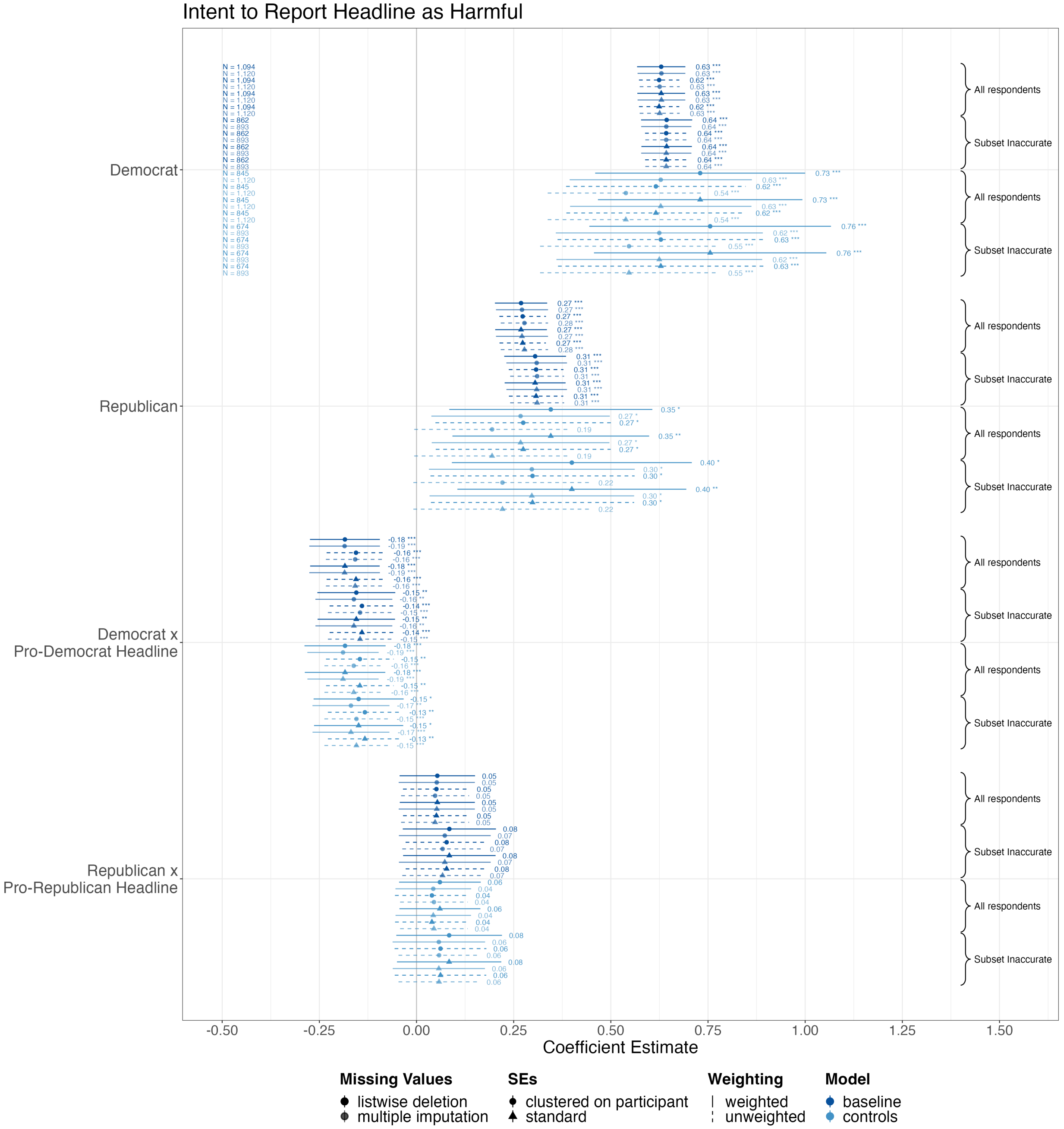

Fig. S8. Robustness checks for models with the first headline only. $^{***}p < 0.001$; $^{**}p < 0.01$; $^{*}p < 0.05$.

Perception of Headline Removal as Censorship

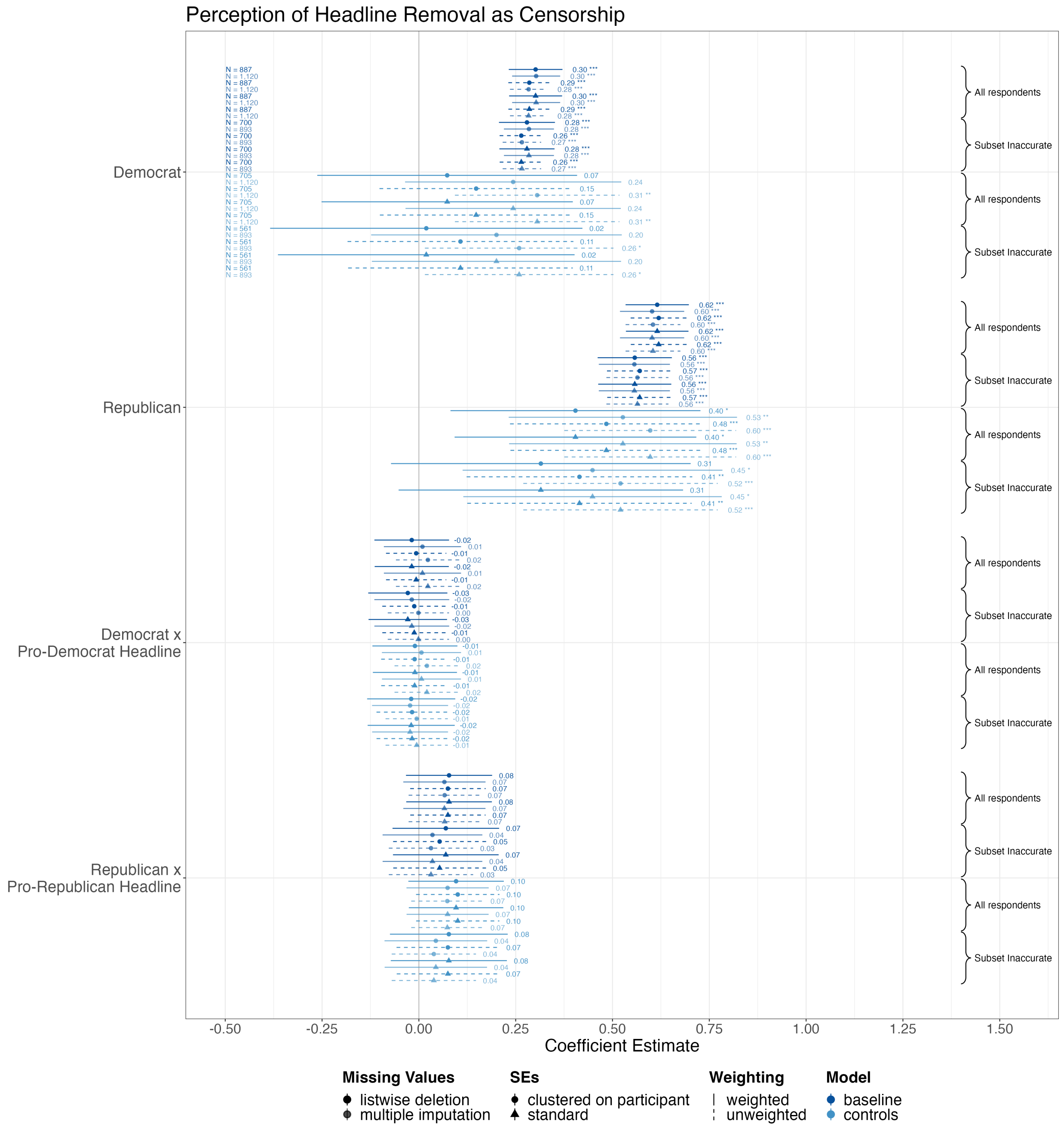

Fig. S9. Robustness checks for models with the first headline only. $^{***}p < 0.001$; $^{**}p < 0.01$; $^{*}p < 0.05$.

S2.1.4 Models Without Interaction

Intent to Remove Headline

Table S12. Regression of Intent to Remove Headline on Partisanship and Alignment

	DV: Intent to Remove Headline	
	All	Inaccurate Subgroup
Democrat	0.69***	0.76***
	(0.02)	(0.02)
Republican	0.34***	0.42***
	(0.02)	(0.03)
R^2	0.58	0.65
Adj. R^2	0.58	0.65
Num. obs.	2190	1721
RMSE	0.46	0.45
N Clusters	1104	1003

$^{***}p < 0.001$; $^{**}p < 0.01$; $^{*}p < 0.05$; $^{\cdot}p < 0.1$

Intent to Report Headline as Harmful

Table S13. Regression of Intent to Report Headline as Harmful on Partisanship and Alignment

	DV: Intent to Report Headline as Harmful	
	All	Inaccurate Subgroup
Democrat	0.49***	0.52***
	(0.02)	(0.02)
Republican	0.27***	0.31***
	(0.02)	(0.02)
R^2	0.42	0.45
Adj. R^2	0.42	0.45
Num. obs.	2192	1720
RMSE	0.47	0.47
N Clusters	1105	1005

$^{***}p < 0.001$; $^{**}p < 0.01$; $^{*}p < 0.05$; $^{\cdot}p < 0.1$

Perception of Headline Removal as Censorship

Table S14. Regression of Perception of Headline Removal as Censorship on Partisanship and Alignment

	DV: Perception of Headline Removal as Censorship	
	All	Inaccurate Subgroup
Democrat	0.29^{***}	0.26^{***}
	(0.02)	(0.02)
Republican	0.65^{***}	0.59^{***}
	(0.03)	(0.03)
R^2	0.53	0.47
Adj. R^2	0.53	0.47
Num. obs.	1774	1407
RMSE	0.46	0.45
N Clusters	958	851

$^{***}p < 0.001$; $^{**}p < 0.01$; $^{*}p < 0.05$; $^{\cdot}p < 0.1$

S2.1.5 Models With Consensus Headlines

The following models include only a subset of the 18 total headlines, that is, only headlines that both Republicans and Democrats rate as inaccurate on average. Specifically, 8 headlines have an average rating below 2 on a 4-point scale ranging from "Not at all accurate" to "Very accurate" for both Democrats and Republicans. We run models with between 2 and 8 of these "consensus headlines." The more headlines are included, the higher the maximum absolute difference between average accuracy ratings of Democrats and Republicans becomes (from about 0.025 for 2 headlines up to 0.5 for 8 headlines, see Table S15).

The following headlines are included in the different sets of consensus headlines in order of increasing maximum absolute difference in accuracy rating between Democrats and Republicans (see section S1.5 for all headlines):

- Set of 8 consensus headlines: Pro-Democrat 4, 1, 7, 5; Pro-Republican 7, 9, 5, 3.
- Set of 7 consensus headlines: Pro-Democrat 4, 1, 7, 5; Pro-Republican 7, 9, 5.
- Set of 6 consensus headlines: Pro-Democrat 4, 1, 7, 5; Pro-Republican 7, 9.
- Set of 5 consensus headlines: Pro-Democrat 4, 1, 7, 5; Pro-Republican 7.
- Set of 4 consensus headlines: Pro-Democrat 4, 1, 7; Pro-Republican 7.
- Set of 3 consensus headlines: Pro-Democrat 4, 1; Pro-Republican 7.
- Set of 2 consensus headlines: Pro-Democrat 4; Pro-Republican 7.

Table S15. Consensus Headline Overview

**Number of headlines**	**Number of pro-Democrat headlines**	**Number of pro-Republican headlines**	**Maximum absolute accuracy difference**
8	4	4	0.4977
7	4	3	0.4950
6	4	2	0.3425
5	4	1	0.2901
4	3	1	0.1567
3	2	1	0.1437
2	1	1	0.0248

Figures S10, S11 and S12 show the consensus headlines analysis for 2 to 8 headlines for the intent to remove headline, the intent to report headline as harmful, and the perception of headline removal as censorship outcome, respectively.

Intent to Remove Headline

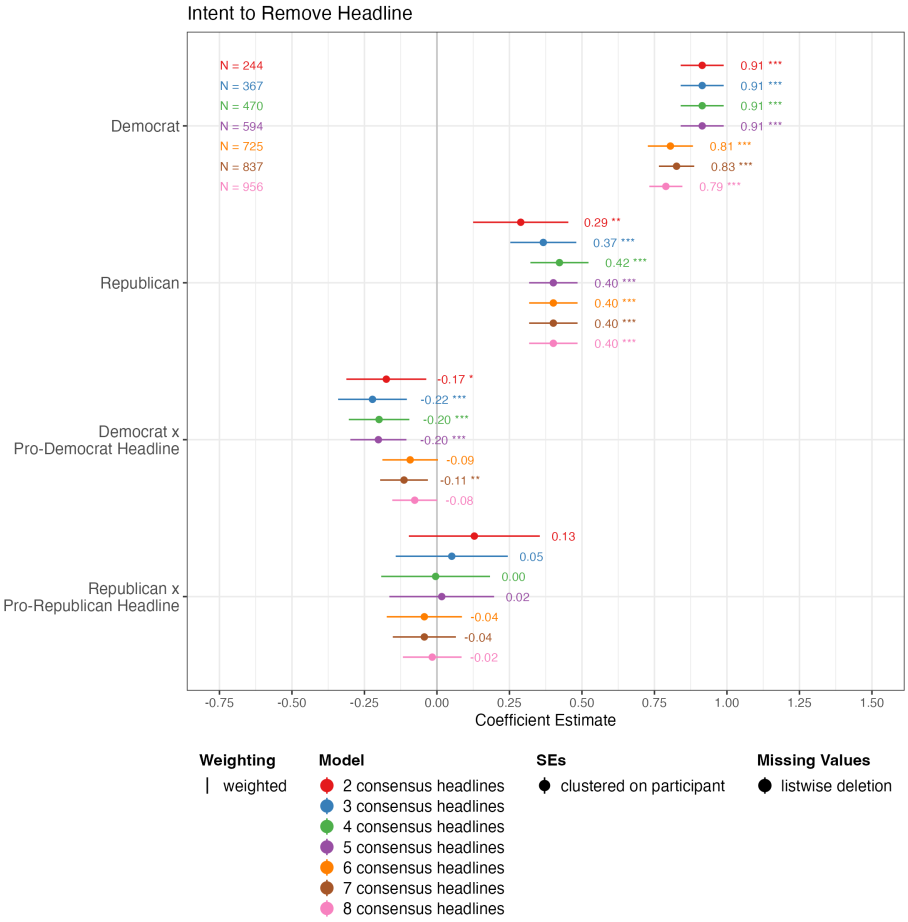

Fig. S10. Robustness checks for models with the consensus headlines. $^{***}p < 0.001$; $^{**}p < 0.01$; $^{*}p < 0.05$. Standard errors clustered on participant for all models; no control variables.

Intent to Report Headline as Harmful

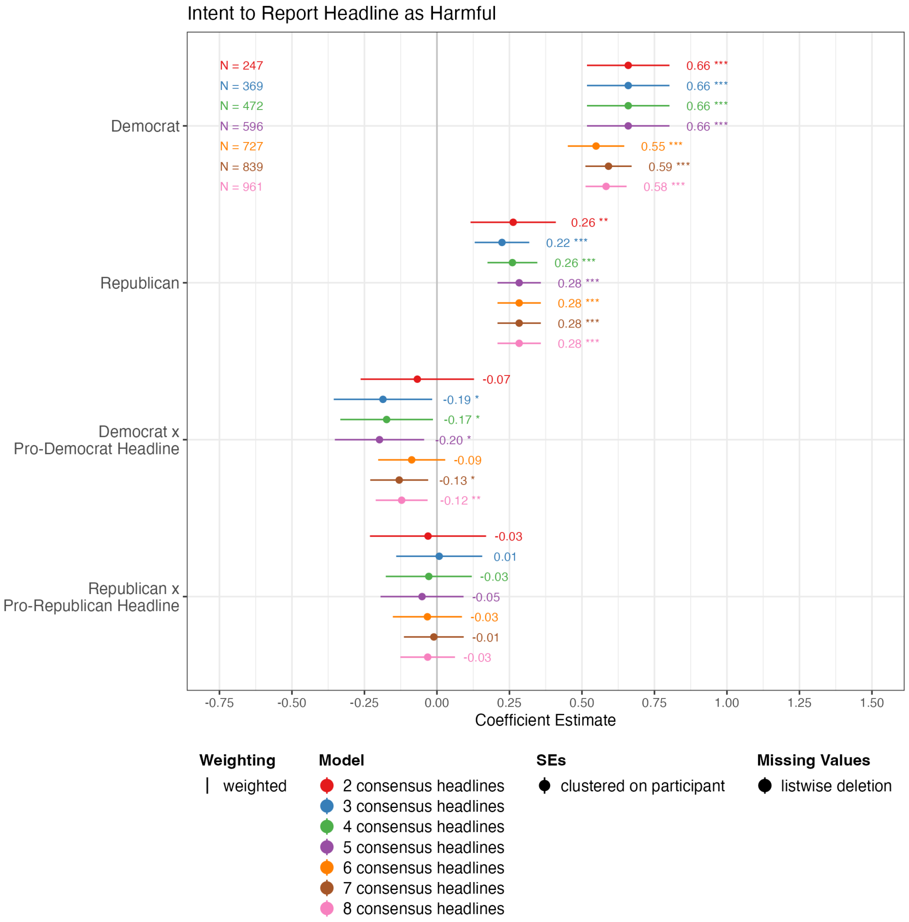

Fig. S11. Robustness checks for models with the consensus headlines. $^{***}p < 0.001$; $^{**}p < 0.01$; $^{*}p < 0.05$. Standard errors clustered on participant for all models; no control variables.

Perception of Headline Removal as Censorship

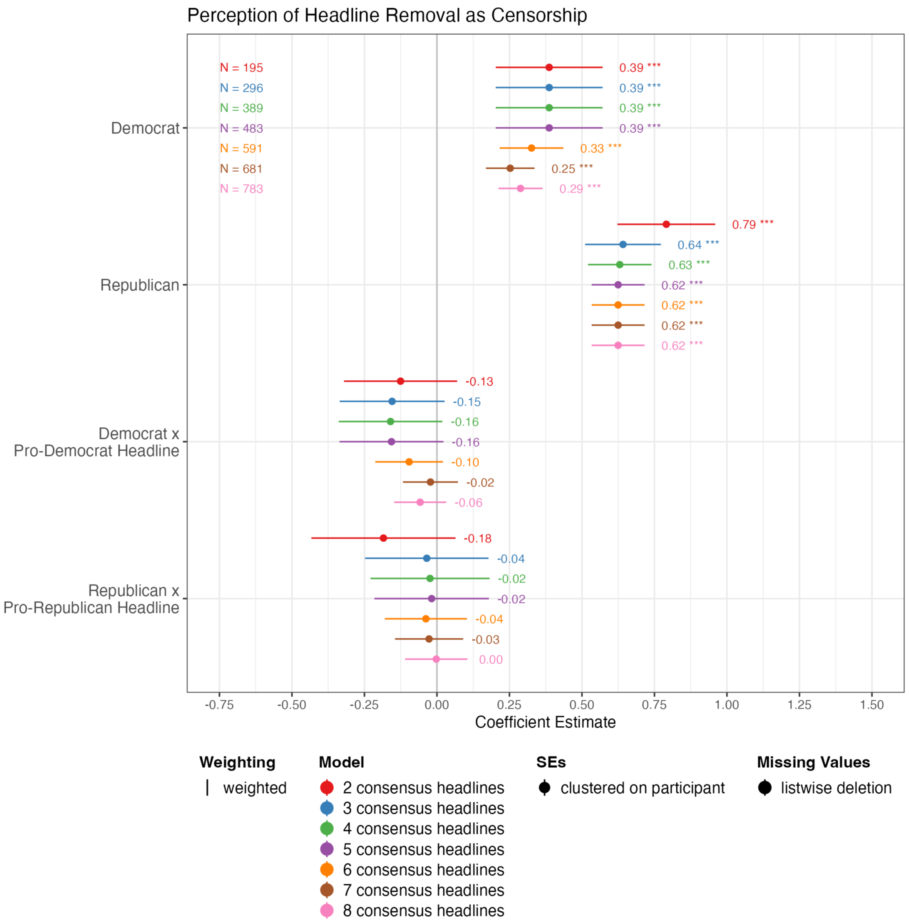

Fig. S12. Robustness checks for models with the consensus headlines. $^{***}p < 0.001$; $^{**}p < 0.01$; $^{*}p < 0.05$. Standard errors clustered on participant for all models; no control variables.

S2.1.6 Models Disaggregated by Headline

Intent to Remove Headline

Table S16. Regression of Intent to Remove Headline on Partisanship by Headline (Pro-Democrat Headlines)

	DV: Intent to Remove Headline								
**Headline**	1	2	3	4	5	6	7	8	9
Democrat Respondent	0.64^{***}	0.40^{***}	0.55^{***}	0.74^{***}	0.70^{***}	0.70^{***}	0.77^{***}	0.64^{***}	0.59^{***}
	(0.07)	(0.07)	(0.07)	(0.06)	(0.07)	(0.07)	(0.06)	(0.06)	(0.08)
Republican Respondent	0.42^{***}	0.49^{***}	0.14^{**}	0.29^{**}	0.34^{***}	0.20^{**}	0.58^{***}	0.51^{***}	0.19^{**}
	(0.08)	(0.08)	(0.05)	(0.08)	(0.08)	(0.06)	(0.10)	(0.08)	(0.05)
R^2	0.54	0.45	0.45	0.65	0.59	0.60	0.70	0.59	0.48
Adj. R^2	0.54	0.44	0.44	0.64	0.58	0.59	0.70	0.58	0.47
Num. obs.	123	128	127	129	124	120	103	122	119
RMSE	0.50	0.49	0.43	0.43	0.45	0.44	0.44	0.50	0.45
N Clusters	123	128	127	129	124	120	103	122	119

$^{***}p < 0.001$; $^{**}p < 0.01$; $^{*}p < 0.05$; $^{\cdot}p < 0.1$

Table S17. Regression of Intent to Remove Headline on Partisanship by Headline (Pro-Republican Headlines)

	DV: Intent to Remove Headline								
**Headline**	1	2	3	4	5	6	7	8	9
Democrat Respondent	0.67^{***}	0.72^{***}	0.68^{***}	0.81^{***}	0.87^{***}	0.74^{***}	0.91^{***}	0.61^{***}	0.71^{***}
	(0.06)	(0.07)	(0.07)	(0.05)	(0.05)	(0.07)	(0.04)	(0.07)	(0.06)
Republican Respondent	0.23^{**}	0.30^{***}	0.47^{***}	0.40^{***}	0.36^{***}	0.34^{***}	0.42^{***}	0.31^{***}	0.31^{***}
	(0.06)	(0.08)	(0.08)	(0.09)	(0.08)	(0.07)	(0.08)	(0.07)	(0.08)
R^2	0.56	0.63	0.60	0.71	0.72	0.61	0.77	0.50	0.58
Adj. R^2	0.55	0.62	0.60	0.70	0.71	0.60	0.76	0.49	0.57
Num. obs.	137	120	119	112	112	129	115	120	131
RMSE	0.45	0.46	0.47	0.44	0.41	0.47	0.39	0.47	0.46
N Clusters	137	120	119	112	112	129	115	120	131

$^{***}p < 0.001$; $^{**}p < 0.01$; $^{*}p < 0.05$; $^{\cdot}p < 0.1$

Intent to Report Headline as Harmful

Table S18. Regression of Intent to Report Headline as Harmful on Partisanship by Headline (Pro-Democrat Headlines)

	DV: Intent to Report Headline as Harmful								
**Headline**	1	2	3	4	5	6	7	8	9
Democrat Respondent	0.33***	0.29***	0.54***	0.59***	0.38***	0.48***	0.51***	0.36***	0.37***
	(0.07)	(0.06)	(0.07)	(0.07)	(0.07)	(0.07)	(0.07)	(0.07)	(0.07)
Republican Respondent	0.20**	0.35***	0.13*	0.26**	0.34***	0.25**	0.36**	0.31***	0.18**
	(0.06)	(0.08)	(0.05)	(0.07)	(0.08)	(0.07)	(0.10)	(0.08)	(0.06)
R^2	0.28	0.32	0.45	0.51	0.36	0.41	0.47	0.34	0.30
Adj. R^2	0.27	0.31	0.44	0.51	0.35	0.40	0.46	0.33	0.29
Num. obs.	122	128	126	132	124	119	103	120	121
RMSE	0.44	0.46	0.42	0.45	0.47	0.47	0.48	0.49	0.44
N Clusters	122	128	126	132	124	119	103	120	121

$^{***}p < 0.001$; $^{**}p < 0.01$; $^{*}p < 0.05$; $^{\cdot}p < 0.1$

Table S19. Regression of Intent to Report Headline as Harmful on Partisanship by Headline (Pro-Republican Headlines)

	DV: Intent to Report Headline as Harmful								
**Headline**	1	2	3	4	5	6	7	8	9
Democrat Respondent	0.44***	0.62***	0.56***	0.66***	0.69***	0.53***	0.66***	0.42***	0.45***
	(0.07)	(0.07)	(0.08)	(0.06)	(0.07)	(0.07)	(0.07)	(0.07)	(0.07)
Republican Respondent	0.28***	0.35***	0.19**	0.36**	0.32***	0.38***	0.23**	0.24**	0.27**
	(0.07)	(0.08)	(0.06)	(0.10)	(0.07)	(0.07)	(0.07)	(0.07)	(0.08)
R^2	0.38	0.55	0.47	0.59	0.56	0.47	0.55	0.35	0.38
Adj. R^2	0.37	0.54	0.46	0.58	0.56	0.46	0.54	0.34	0.37
Num. obs.	137	120	122	110	112	130	115	120	131
RMSE	0.47	0.49	0.44	0.48	0.46	0.50	0.43	0.46	0.47
N Clusters	137	120	122	110	112	130	115	120	131

$^{***}p < 0.001$; $^{**}p < 0.01$; $^{*}p < 0.05$; $^{\cdot}p < 0.1$

Perception of Headline Removal as Censorship

Table S20. Regression of Perception of Headline Removal as Censorship on Partisanship by Headline (Pro-Democrat Headlines)

	DV: Perception of Headline Removal as Censorship								
**Headline**	1	2	3	4	5	6	7	8	9
Democrat Respondent	0.20**	0.31***	0.43***	0.26**	0.24*	0.42***	0.22**	0.18**	0.39***
	(0.06)	(0.07)	(0.08)	(0.07)	(0.08)	(0.08)	(0.06)	(0.05)	(0.09)
Republican Respondent	0.53***	0.50***	0.77***	0.79***	0.61***	0.81***	0.60***	0.56***	0.66***
	(0.09)	(0.08)	(0.07)	(0.08)	(0.09)	(0.07)	(0.11)	(0.09)	(0.08)
R^2	0.45	0.43	0.65	0.62	0.51	0.67	0.46	0.46	0.57
Adj. R^2	0.44	0.42	0.65	0.61	0.50	0.67	0.45	0.45	0.56
Num. obs.	101	102	97	105	94	103	93	94	98
RMSE	0.47	0.48	0.47	0.42	0.46	0.45	0.43	0.45	0.49
N Clusters	101	102	97	105	94	103	93	94	98

$^{***}p < 0.001$; $^{**}p < 0.01$; $^{*}p < 0.05$; $^{\cdot}p < 0.1$

Table S21. Regression of Perception of Headline Removal as Censorship on Partisanship by Headline (Pro-Republican Headlines)

	DV: Perception of Headline Removal as Censorship								
**Headline**	1	2	3	4	5	6	7	8	9
Democrat Respondent	0.42***	0.21**	0.38***	0.26***	0.07*	0.22*	0.39***	0.28***	0.28***
	(0.08)	(0.07)	(0.08)	(0.06)	(0.03)	(0.08)	(0.09)	(0.07)	(0.07)
Republican Respondent	0.73***	0.65***	0.70***	0.55***	0.62***	0.67***	0.61***	0.67***	0.57***
	(0.07)	(0.09)	(0.08)	(0.10)	(0.09)	(0.08)	(0.09)	(0.08)	(0.09)
R^2	0.63	0.50	0.58	0.43	0.57	0.57	0.52	0.57	0.48
Adj. R^2	0.62	0.49	0.58	0.42	0.56	0.56	0.51	0.56	0.47
Num. obs.	114	104	102	93	90	92	90	94	108
RMSE	0.48	0.45	0.46	0.47	0.39	0.45	0.47	0.46	0.48
N Clusters	114	104	102	93	90	92	90	94	108

$^{***}p < 0.001$; $^{**}p < 0.01$; $^{*}p < 0.05$; $^{\cdot}p < 0.1$

S2.1.7 Models With Triple Interaction Between Accuracy Question Order, Participant Partisanship, and Headline Alignment

Previous research has found that prompting respondents with a question about accuracy may increase their attention to accuracy (*28, 29*). Given that we find that Democrats who rated the headlines as inaccurate were less likely to exhibit party promotion, we investigated whether an accuracy nudge might reduce party promotion. In Tables S22, S23, and S24, we interact the party promotion effect with an indicator of whether accuracy was asked first (third column). This analysis includes only the first headline that participants rated, because when participants rated the second headline, they had already seen the accuracy question regardless of whether they were randomized to see the accuracy question before or after outcomes, implying that accuracy had already been primed for all groups. This analysis was not pre-registered. While the estimates on these triple interactions are almost all positive, which would suggest that an accuracy nudge could be used to reduce party promotion, none of them are significant on any outcome. In addition, we show models that include only the subset of participants who either saw the accuracy question first (first column) or second (second column). We believe additional research in this area is may be warranted to see whether the accuracy nudge or a similar treatment could be used to alleviate the party promotion effect.

Intent to Remove Headline

Table S22. Regression of Intent to Remove Headline on Partisanship and Alignment

	DV: Intent to Remove Headline		
	Accuracy Question First	Accuracy Question Second	Accuracy Question Order Interaction
Democrat	0.76^{***}	0.80^{***}	0.80^{***}
	(0.04)	(0.04)	(0.04)
Republican	0.36^{***}	0.40^{***}	0.40^{***}
	(0.05)	(0.05)	(0.04)
Democrat x Pro-Democrat Headline	-0.07	-0.16^{**}	-0.16^{**}
	(0.06)	(0.06)	(0.06)
Republican x Pro-Republican Headline	0.03	-0.08	-0.08
	(0.07)	(0.08)	(0.07)
Accuracy Question First			-0.04
			(0.05)
Accuracy Question First x Democrat x Pro-Democrat Headline			0.09
			(0.08)
Accuracy Question First x Republican x Pro-Republican Headline			0.11
			(0.09)
R^2	0.60	0.63	0.61
Adj. R^2	0.60	0.62	0.61
Num. obs.	565	531	1096
RMSE	0.46	0.46	0.46
N Clusters	565	531	1096

$^{***}p < 0.001$; $^{**}p < 0.01$; $^{*}p < 0.05$; $^{\cdot}p < 0.1$

Intent to Report Headline as Harmful

Table S23. Regression of Intent to Report Headline as Harmful on Partisanship and Alignment

	DV: Intent to Report Headline as Harmful		
	Accuracy Question First	Accuracy Question Second	Accuracy Question Order Interaction
Democrat	0.58^{***}	0.68^{***}	0.64^{***}
	(0.05)	(0.04)	(0.04)
Republican	0.30^{***}	0.23^{***}	0.28^{***}
	(0.05)	(0.04)	(0.04)
Democrat x Pro-Democrat Headline	-0.09	-0.27^{***}	-0.23^{***}
	(0.07)	(0.06)	(0.06)
Republican x Pro-Republican Headline	0.07	0.02	-0.02
	(0.07)	(0.07)	(0.07)
Accuracy Question First			-0.02
			(0.05)
Accuracy Question First x Democrat x Pro-Democrat Headline			0.09
			(0.08)
Accuracy Question First x Republican x Pro-Republican Headline			0.14
			(0.08)
R^2	0.46	0.49	0.47
Adj. R^2	0.46	0.49	0.47
Num. obs.	566	528	1094
RMSE	0.48	0.46	0.47
N Clusters	566	528	1094

$^{***}p < 0.001$; $^{**}p < 0.01$; $^{*}p < 0.05$; $^{\cdot}p < 0.1$

Perception of Headline Removal as Censorship

Table S24. Regression of Perception of Headline Removal as Censorship on Partisanship and Alignment

	DV: Perception of Headline Removal as Censorship		
	Accuracy Question First	Accuracy Question Second	Accuracy Question Order Interaction
Democrat	0.28^{***}	0.32^{***}	0.32^{***}
	(0.05)	(0.05)	(0.05)
Republican	0.61^{***}	0.62^{***}	0.63^{***}
	(0.06)	(0.06)	(0.05)
Democrat x Pro-Democrat Headline	0.04	-0.07	-0.06
	(0.07)	(0.07)	(0.07)
Republican x Pro-Republican Headline	0.06	0.10	0.09
	(0.08)	(0.08)	(0.07)
Accuracy Question First			-0.03
			(0.05)
Accuracy Question First x Democrat x Pro-Democrat Headline			0.09
			(0.09)
Accuracy Question First x Republican x Pro-Republican Headline			-0.03
			(0.09)
R^2	0.54	0.55	0.54
Adj. R^2	0.53	0.55	0.54
Num. obs.	455	432	887
RMSE	0.46	0.46	0.46
N Clusters	455	432	887

$^{***}p < 0.001$; $^{**}p < 0.01$; $^{*}p < 0.05$; $^{\cdot}p < 0.1$

S2.2 Mediation Analysis

As pre-registered, we conducted a mediation analysis, using the `mediation` package in R (*72*), to test whether the effect of one variable (partisan alignment) on another (intent to remove headline, intent to report headline as harmful) is driven by an intermediary variable (perceived accuracy of headline). We conduct this test for Democratic respondents only because we only find a party promotion effect for Democrats. This tests whether our estimate of party promotion is mediated by perceived accuracy.

Going beyond the pre-registration, we also conduct a mediation analysis of the effect of partisanship on all outcomes (intent to remove headline, intent to report headline, perception of headline removal as censorship), mediated by perceived accuracy of the headline. We conduct this test for all respondents. This tests whether our estimate of the preference gap is mediated by perceived accuracy.

In the case of partisan alignment, and analogously for partisanship, the Average Causal Mediation Effect (ACME) is the Total Effect that alignment has on the outcome variable of interest minus the Average Direct Effect (ADE), which is the effect of alignment on the outcome without taking the indirect path through accuracy into account. The mediation analyses are based on unweighted models with standard errors clustered on participants (we used the default clustering option in the `mediation` function in the `mediation` R package to cluster standard errors on participants).

For the mediation analyses, we used the following models:

$$\text{Mediator model: accuracy}_{ia} = \beta_0 + \beta_1 \cdot \text{aligned}_{ia} + \varepsilon_{ia} \tag{S5}$$

$$\text{Outcome model: } Y_{ia} = \beta_0 + \beta_1 \cdot \text{accuracy}_{ia} + \beta_2 \cdot \text{aligned}_{ia} + \varepsilon_{ia} \tag{S6}$$

accuracy_{ia} is the accuracy rating for individual i and headline a, Y_{ia} is the binary outcome measure, and aligned_{ia} indicates whether participant and headline partisanship were aligned. In the models estimating the effect of partisanship instead of alignment, aligned_{ia} is replaced with partisanship_i, which indicates the partisanship for respondent i with Democrat coded as 1 and Republican coded as 0.

Mediation analysis, as we have conducted it, relies on a particular version of sequential ignorability where 1) the treatment is assumed to be ignorable given pre-treatment covariates, and 2) the mediator variable is assumed to be ignorable given observed values of treatment and pre-treatment covariates. This second part of sequential ignorability may not hold in our case, because accuracy perception (and indeed any attitude or opinion, which cannot be randomly assigned) may not be ignorable given treatment and pre-treatment covariates. The identification assumption is not directly testable, but sensitivity analysis allows us to examine the robustness of the findings to the possible existence of an unmeasured pre-treatment confounder (*53*). In Figures S13 and S15, which show two different operationalizations of accuracy perception, we plot the parameter ρ (x-axis), which is calculated as the correlation between the error term of the mediator and outcome models and can be interpreted as the strength of confounding between the mediator and outcome, and the estimated ACME and ADE (with 95% confidence intervals based on the Delta method) for the accuracy perception mediator for differing values of ρ and compare it to the point estimate of the average mediation effect under the sequential ignorability assumption.

The mediation analysis results for party promotion are shown in Tables S25 and S26 for two different operationalizations of accuracy (4-point scale vs. binarized). The effect of political align-

ment on intent to remove and intent to report a false headline as harmful is fully mediated by accuracy in the models including control variables and using the 4-point accuracy operationalization, and partially mediated in the models without control variables. This indicates that perceptions of accuracy explains at least part, but perhaps not all of the party promotion effect for Democrats we find.

The sensitivity analysis for party promotion indicates that our conclusion that accuracy mediates party promotion among Democrats is plausible given even fairly large departures from the ignorability of the mediator due to a pre-treatment confounder. We observe this from Figure S13 for intent to remove and intent to report a headline as harmful because the direction of the ACME under sequential ignorability (represented by the dashed horizontal line) would be maintained unless ρ is less than -0.3 and -0.2, respectively. The results are substantively similar for binarized accuracy ratings (see Figure S15), and when control variables are included (see Figures S14 and S16). This sensitivity analysis suggests that the fact gap partially—but not completely—explains party promotion for Democrats.

The mediation analysis results for the preference gap are shown in Tables S27 and S28 for two different operationalizations of accuracy (4-point scale vs. binarized). In the main analysis, we saw evidence of a preference gap given significant and divergent main effects for Democrat and Republican partisanship. The mediation analysis suggests that perceptions of accuracy do not explain most of the preference gap. While the ACME is significant for all outcomes, it is very small, implying that perceptions of accuracy mediate the relationship between partisanship (Republican partisanship is considered the control, Democrat partisanship the treatment for this analysis) and intent to remove a headline, report a headline as harmful, or consider headline removal as censorship only to a small extent.

The sensitivity analysis for the preference gap indicates that the finding that accuracy mediates the preference gap is not robust to large departures from the ignorability of the mediator. We observe this from Figure S17 for all outcomes because ρ for the ACME is very small in absolute value, while ρ is large in absolute value for the ADE. The results are substantively similar for binarized accuracy ratings (see Figure S19), and when control variables are included (see Figures S18 and S20).

However, these sensitivity analyses do not address the possible existence of post-treatment confounders, so this test remains imperfect.

S2.2.1 Mediation of the Effect of Partisan Alignment

4-Point Accuracy Variable as Mediator

Table S25. Effect of Alignment Mediated by Accuracy for Democrats (4-Point Accuracy Variable)

Measure	Estimate	95% CI Lower	95% CI Upper	p-value
Intent to Remove Headline — Without Controls				
ACME	−0.065	−0.082	−0.049	< 0.001
ADE	−0.039	−0.073	−0.001	0.034
Total Effect	−0.103	−0.140	−0.068	< 0.001
Proportion Mediated	0.624	0.439	0.982	< 0.001
N Observations	1302			
N Simulations	1000			
Intent to Remove Headline — With Controls				
ACME	−0.074	−0.096	−0.053	< 0.001
ADE	−0.032	−0.074	0.007	0.132
Total Effect	−0.105	−0.150	−0.064	< 0.001
Proportion Mediated	0.702	0.470	1.097	< 0.001
N Observations	995			
N Simulations	1000			
Intent to Report Headline as Harmful — Without Controls				
ACME	−0.035	−0.051	−0.022	< 0.001
ADE	−0.074	−0.114	−0.031	< 0.001
Total Effect	−0.109	−0.149	−0.069	< 0.001
Proportion Mediated	0.321	0.184	0.557	< 0.001
N Observations	1301			
N Simulations	1000			
Intent to Report Headline as Harmful — With Controls				
ACME	−0.052	−0.071	−0.034	< 0.001
ADE	−0.040	−0.091	0.007	0.114
Total Effect	−0.092	−0.141	−0.044	< 0.001
Proportion Mediated	0.563	0.319	1.159	< 0.001
N Observations	993			
N Simulations	1000			

Note: Mediation models were run with standard errors clustered on participants and without weigthing observations using a dataset in which missing values were addressed using listwise deletion.

Fig. S13. Mediation Sensitivity Analysis for the Effect of Alignment Mediated by Accuracy for Democrats (4-Point Accuracy Variable, No Controls)

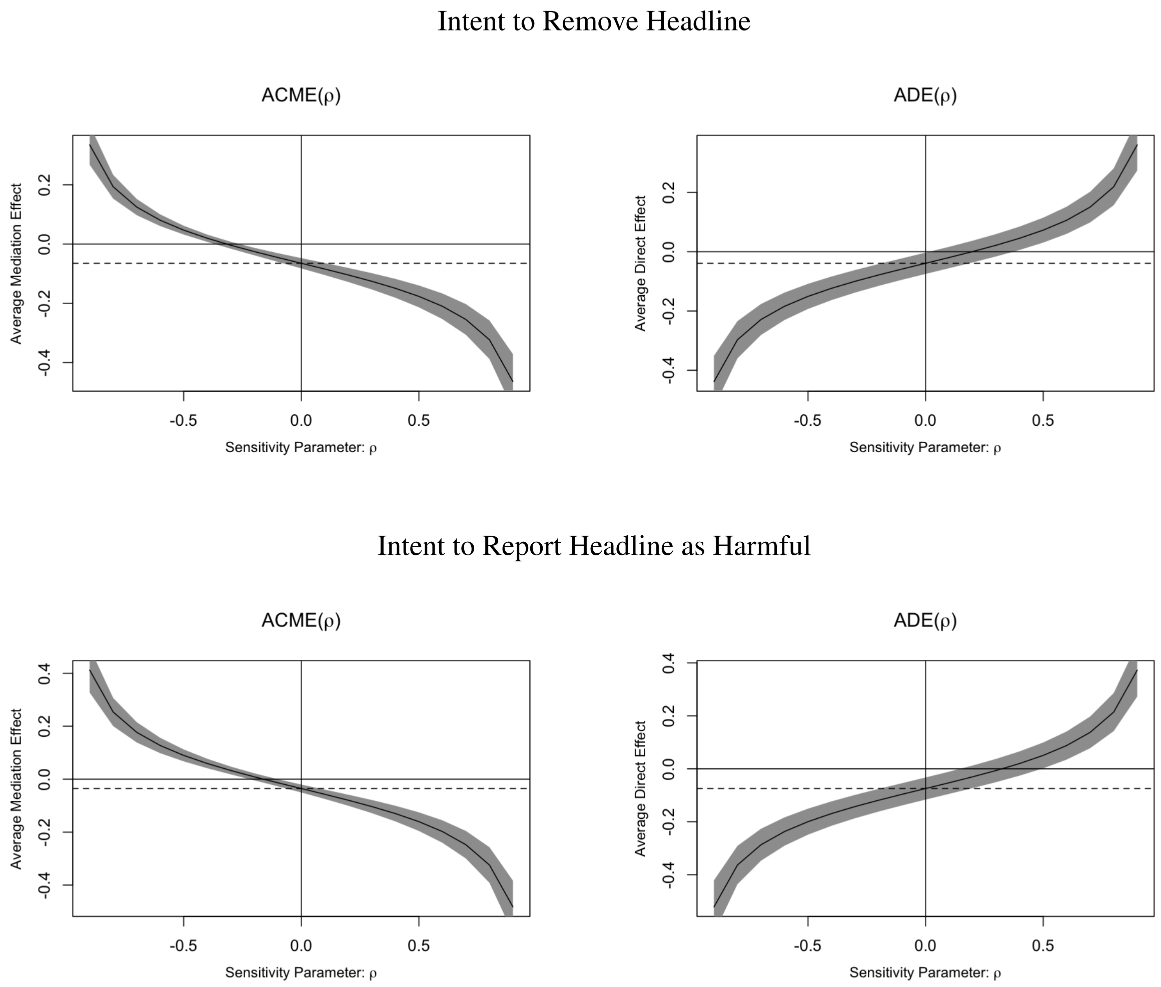

Fig. S14. Mediation Sensitivity Analysis for the Effect of Alignment Mediated by Accuracy for Democrats (4-Point Accuracy Variable, With Controls)

Intent to Remove Headline

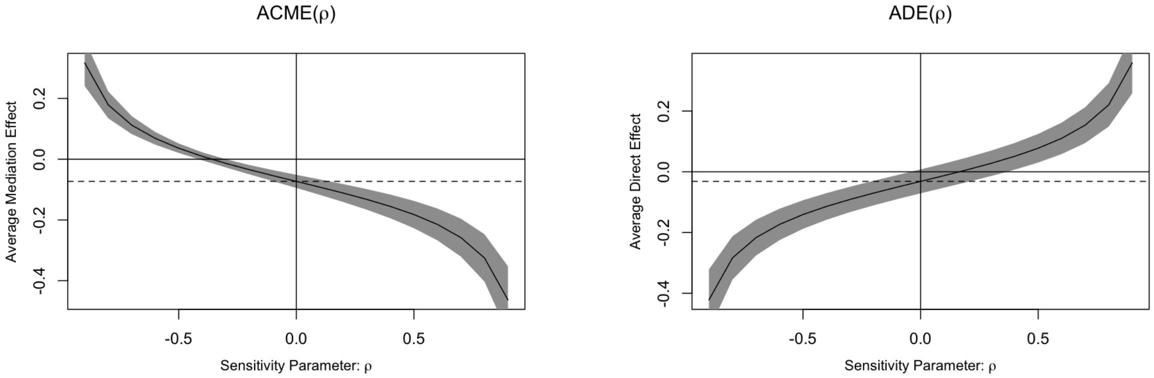

Intent to Report Headline as Harmful

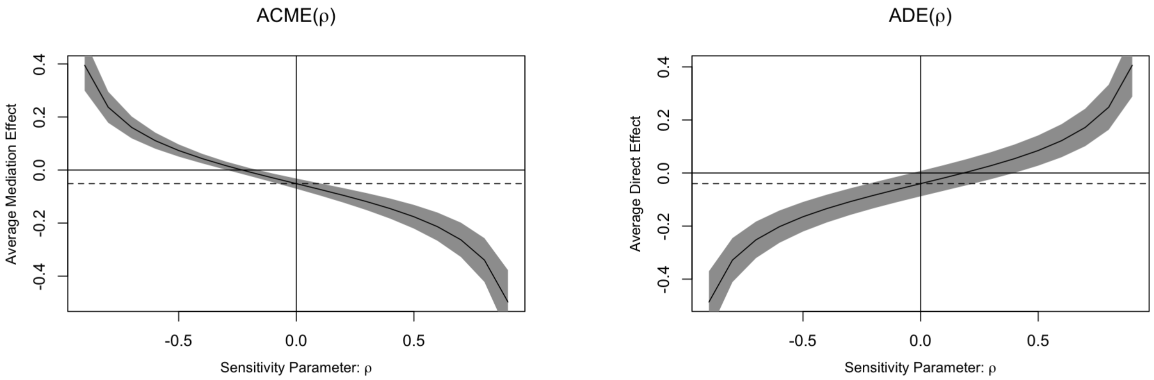

Binary Accuracy Variable as Mediator

Table S26. Effect of Alignment Mediated by Accuracy for Democrats (Binary Accuracy Variable)

**Measure**	**Estimate**	**95% CI Lower**	**95% CI Upper**	**p-value**
Intent to Remove Headline — Without Controls				
ACME	−0.049	−0.064	−0.035	< 0.001
ADE	−0.055	−0.089	−0.019	< 0.001
Total Effect	−0.103	−0.139	−0.067	< 0.001
Proportion Mediated	0.466	0.321	0.737	< 0.001
N Observations	1302			
N Simulations	1000			
Intent to Remove Headline — With Controls				
ACME	−0.056	−0.077	−0.037	< 0.001
ADE	−0.049	−0.091	−0.012	0.012
Total Effect	−0.105	−0.150	−0.064	< 0.001
Proportion Mediated	0.532	0.345	0.820	< 0.001
N Observations	995			
N Simulations	1000			
Intent to Report Headline as Harmful — Without Controls				
ACME	−0.019	−0.030	−0.009	< 0.001
ADE	−0.091	−0.131	−0.049	< 0.001
Total Effect	−0.109	−0.149	−0.069	< 0.001
Proportion Mediated	0.167	0.074	0.325	< 0.001
N Observations	1301			
N Simulations	1000			
Intent to Report Headline as Harmful — With Controls				
ACME	−0.029	−0.044	−0.016	< 0.001
ADE	−0.063	−0.113	−0.017	0.008
Total Effect	−0.092	−0.141	−0.045	< 0.001
Proportion Mediated	0.318	0.160	0.645	< 0.001
N Observations	993			
N Simulations	1000			

Note: Mediation models were run with standard errors clustered on participants and without weigthing observations using a dataset in which missing values were addressed using listwise deletion.

Fig. S15. Mediation Sensitivity Analysis for the Effect of Alignment Mediated by Accuracy for Democrats (Binary Accuracy Variable, No Controls)

Intent to Remove Headline

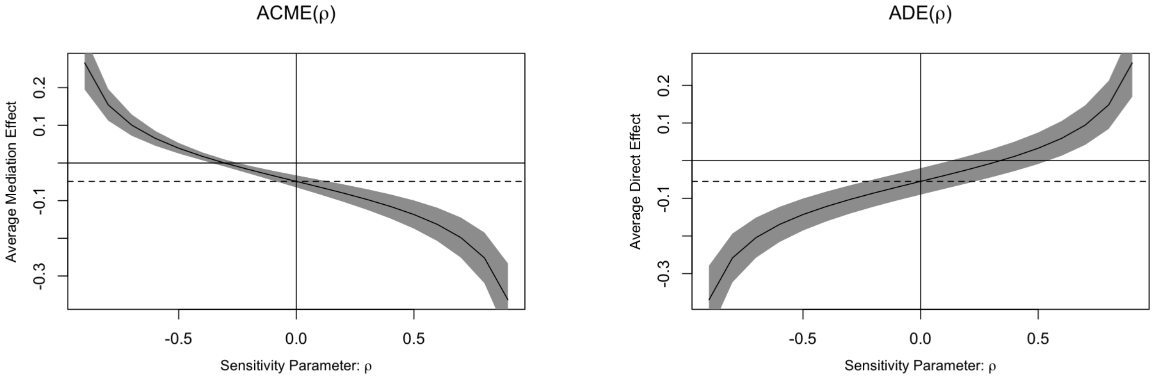

Intent to Report Headline as Harmful

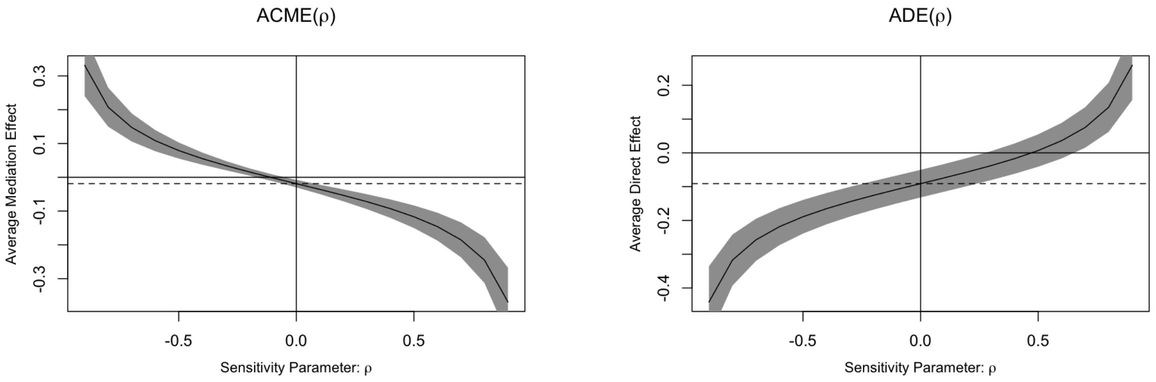

Fig. S16. Mediation Sensitivity Analysis for the Effect of Alignment Mediated by Accuracy for Democrats (Binary Accuracy Variable, With Controls)

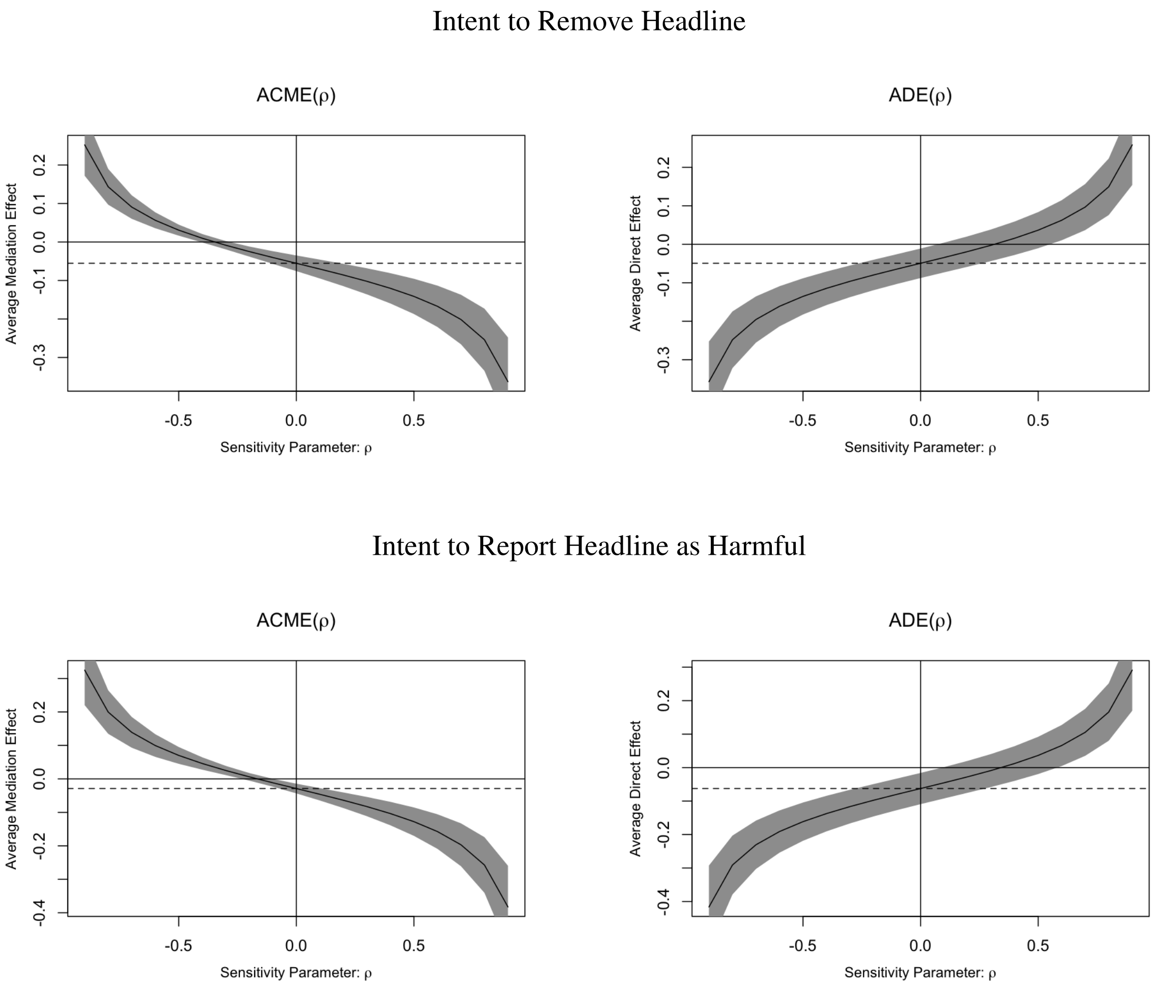

S2.2.2 Mediation of the Effect of Partisanship (Democrat as Treatment)

4-Point Accuracy Variable as Mediator

Table S27. Effect of Partisanship (Democrat as Treatment) Mediated by Accuracy (4-Point Accuracy Variable)

Measure	Estimate	95% CI Lower	95% CI Upper	p-value
Intent to Remove Headline — Without Controls				
ACME	0.040	0.026	0.055	< 0.001
ADE	0.317	0.268	0.367	< 0.001
Total Effect	0.358	0.309	0.407	< 0.001
Proportion Mediated	0.113	0.074	0.158	< 0.001
N Observations	2158			
N Simulations	1000			
Intent to Remove Headline — With Controls				
ACME	0.052	0.032	0.073	< 0.001
ADE	0.303	0.233	0.370	< 0.001
Total Effect	0.355	0.285	0.421	< 0.001
Proportion Mediated	0.146	0.090	0.210	< 0.001
N Observations	1668			
N Simulations	1000			
Intent to Report Headline as Harmful — Without Controls				
ACME	0.023	0.013	0.032	< 0.001
ADE	0.196	0.147	0.246	< 0.001
Total Effect	0.219	0.171	0.267	< 0.001
Proportion Mediated	0.103	0.060	0.161	< 0.001
N Observations	2159			
N Simulations	1000			
Intent to Report Headline as Harmful — With Controls				
ACME	0.032	0.018	0.046	< 0.001
ADE	0.183	0.116	0.250	< 0.001
Total Effect	0.215	0.147	0.283	< 0.001
Proportion Mediated	0.147	0.081	0.246	< 0.001
N Observations	1668			
N Simulations	1000			
Perception of Headline Removal as Censorship — Without Controls				
ACME	−0.028	−0.041	−0.017	< 0.001
ADE	−0.345	−0.401	−0.287	< 0.001
Total Effect	−0.373	−0.427	−0.314	< 0.001
Proportion Mediated	0.076	0.045	0.111	< 0.001
N Observations	1751			
N Simulations	1000			
Perception of Headline Removal as Censorship — With Controls				
ACME	−0.027	−0.042	−0.015	< 0.001
ADE	−0.352	−0.426	−0.276	< 0.001
Total Effect	−0.380	−0.456	−0.307	< 0.001
Proportion Mediated	0.070	0.037	0.116	< 0.001
N Observations	1389			
N Simulations	1000			

Note: Mediation models were run with standard errors clustered on participants and without weigthing observations using a dataset in which missing values were addressed using listwise deletion.

Fig. S17. Mediation Sensitivity Analysis for the Effect of Partisanship (Democrat as Treatment) Mediated by Accuracy (4-Point Accuracy Variable, No Controls)

Intent to Remove Headline

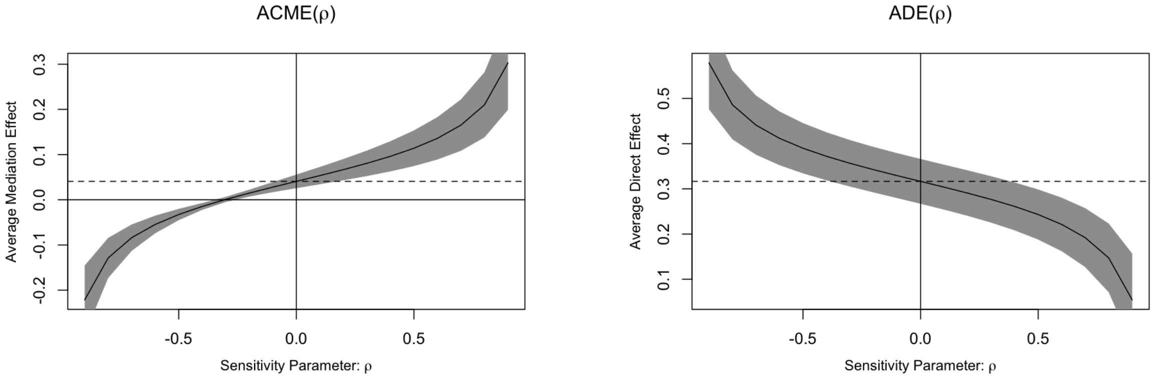

Intent to Report Headline as Harmful

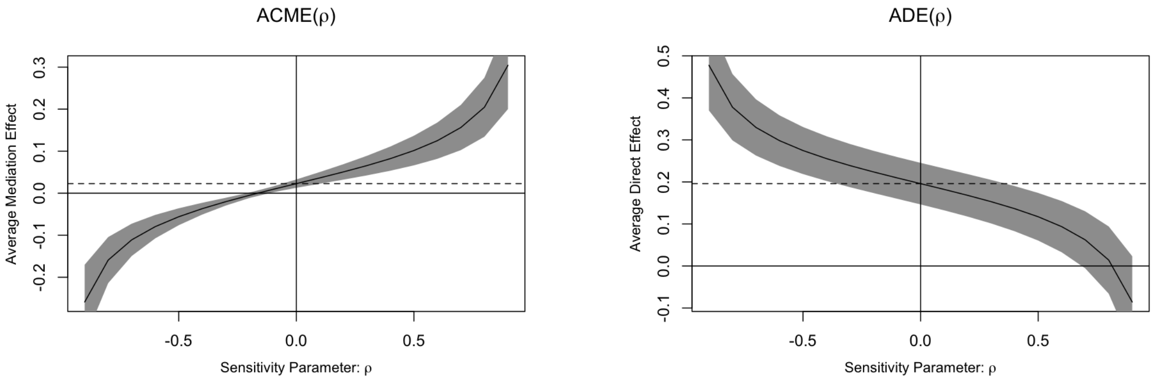

Perception of Headline Removal as Censorship

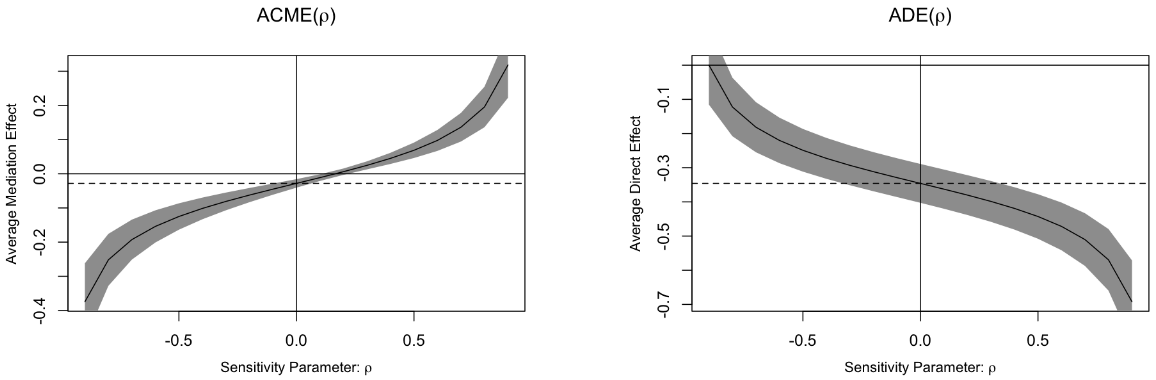

Fig. S18. Mediation Sensitivity Analysis for the Effect of Partisanship (Democrat as Treatment) Mediated by Accuracy (4-Point Accuracy Variable, With Controls)

Intent to Remove Headline

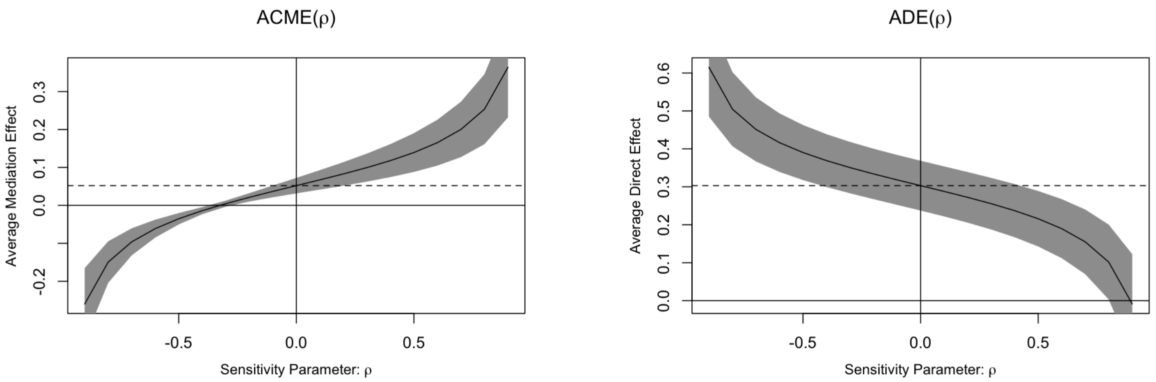

Intent to Report Headline as Harmful

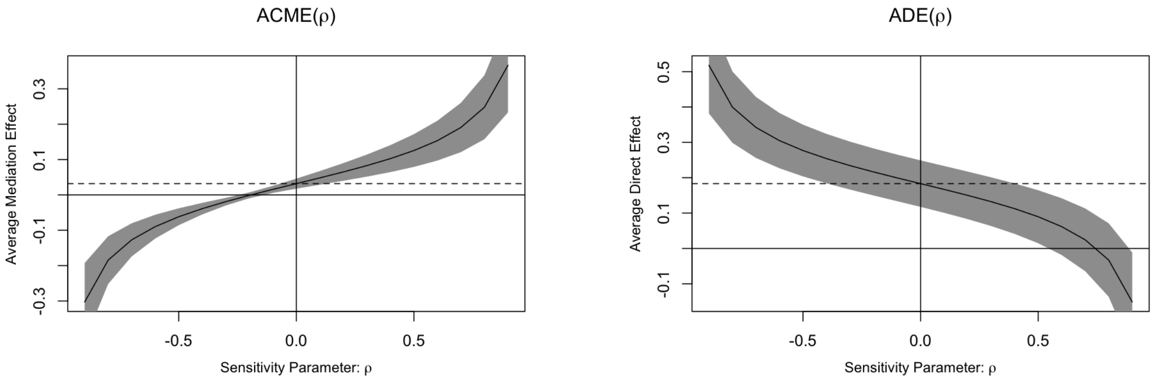

Perception of Headline Removal as Censorship

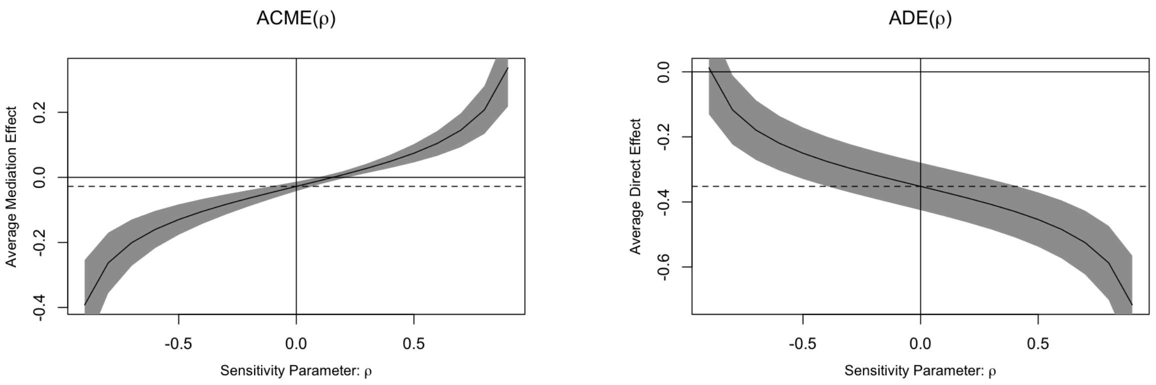

Binary Accuracy Variable as Mediator

Table S28. Effect of Partisanship (Democrat as Treatment) Mediated by Accuracy (Binary Accuracy Variable)

Measure	Estimate	95% CI Lower	95% CI Upper	p-value
Intent to Remove Headline — Without Controls				
ACME	0.027	0.014	0.040	0.002
ADE	0.331	0.283	0.382	< 0.001
Total Effect	0.358	0.309	0.407	< 0.001
Proportion Mediated	0.074	0.040	0.113	0.002
N Observations	2158			
N Simulations	1000			
Intent to Remove Headline — With Controls				
ACME	0.031	0.015	0.050	< 0.001
ADE	0.323	0.256	0.391	< 0.001
Total Effect	0.355	0.284	0.424	< 0.001
Proportion Mediated	0.089	0.043	0.140	< 0.001
N Observations	1668			
N Simulations	1000			
Intent to Report Headline as Harmful — Without Controls				
ACME	0.012	0.006	0.021	0.002
ADE	0.207	0.159	0.257	< 0.001
Total Effect	0.219	0.172	0.268	< 0.001
Proportion Mediated	0.056	0.027	0.098	0.002
N Observations	2159			
N Simulations	1000			
Intent to Report Headline as Harmful — With Controls				
ACME	0.016	0.007	0.027	< 0.001
ADE	0.199	0.132	0.265	< 0.001
Total Effect	0.215	0.147	0.283	< 0.001
Proportion Mediated	0.076	0.032	0.138	< 0.001
N Observations	1668			
N Simulations	1000			
Perception of Headline Removal as Censorship — Without Controls				
ACME	-0.023	-0.034	-0.014	< 0.001
ADE	-0.350	-0.405	-0.292	< 0.001
Total Effect	-0.373	-0.427	-0.315	< 0.001
Proportion Mediated	0.062	0.036	0.093	< 0.001
N Observations	1751			
N Simulations	1000			
Perception of Headline Removal as Censorship — With Controls				
ACME	-0.022	-0.035	-0.011	< 0.001
ADE	-0.358	-0.432	-0.282	< 0.001
Total Effect	-0.380	-0.455	-0.307	< 0.001
Proportion Mediated	0.055	0.028	0.095	< 0.001
N Observations	1389			
N Simulations	1000			

Note: Mediation models were run with standard errors clustered on participants and without weigthing observations using a dataset in which missing values were addressed using listwise deletion.

Fig. S19. Mediation Sensitivity Analysis for the Effect of Partisanship (Democrat as Treatment) Mediated by Accuracy (Binary Accuracy Variable, No Controls)

Intent to Remove Headline

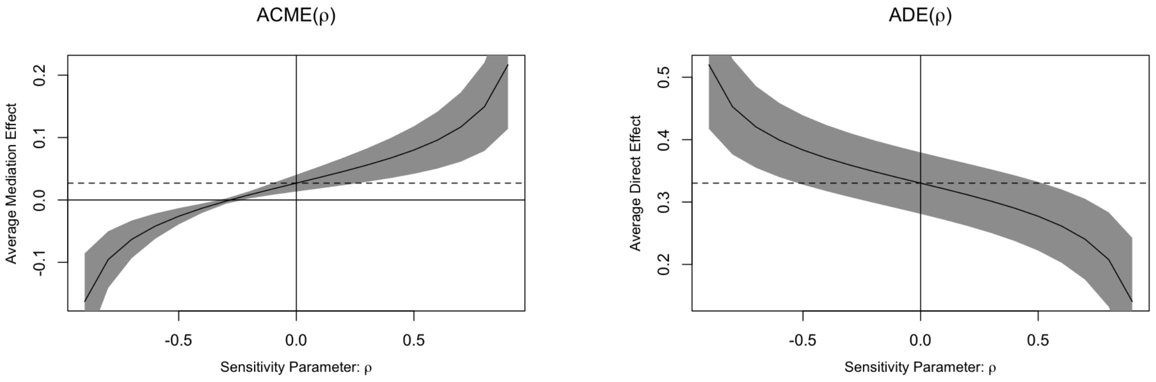

Intent to Report Headline as Harmful

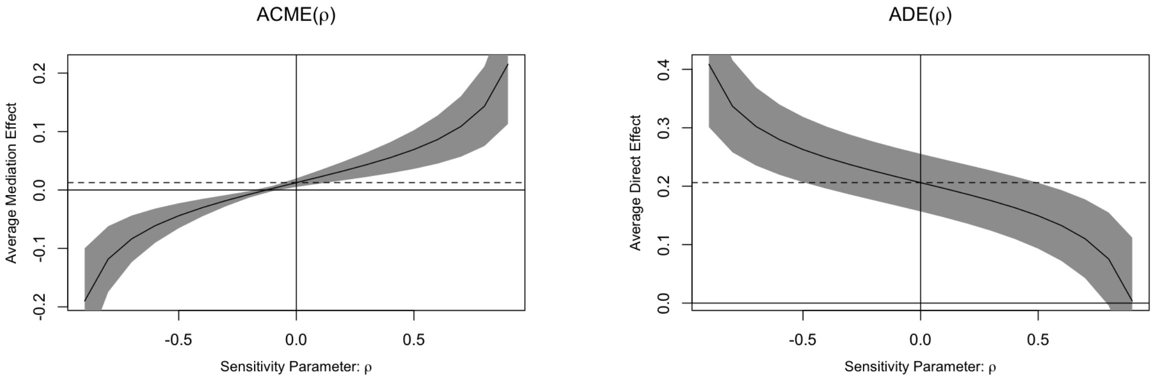

Perception of Headline Removal as Censorship

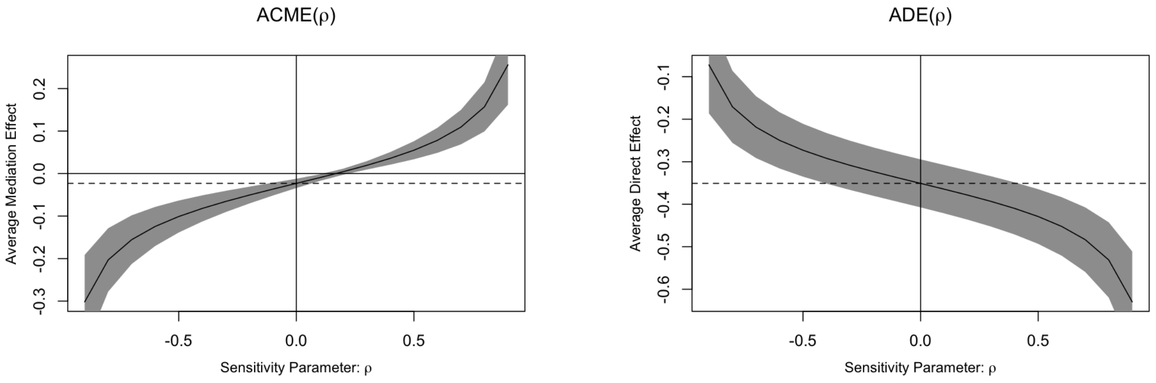

Fig. S20. Mediation Sensitivity Analysis for the Effect of Partisanship (Democrat as Treatment) Mediated by Accuracy (Binary Accuracy Variable, With Controls)

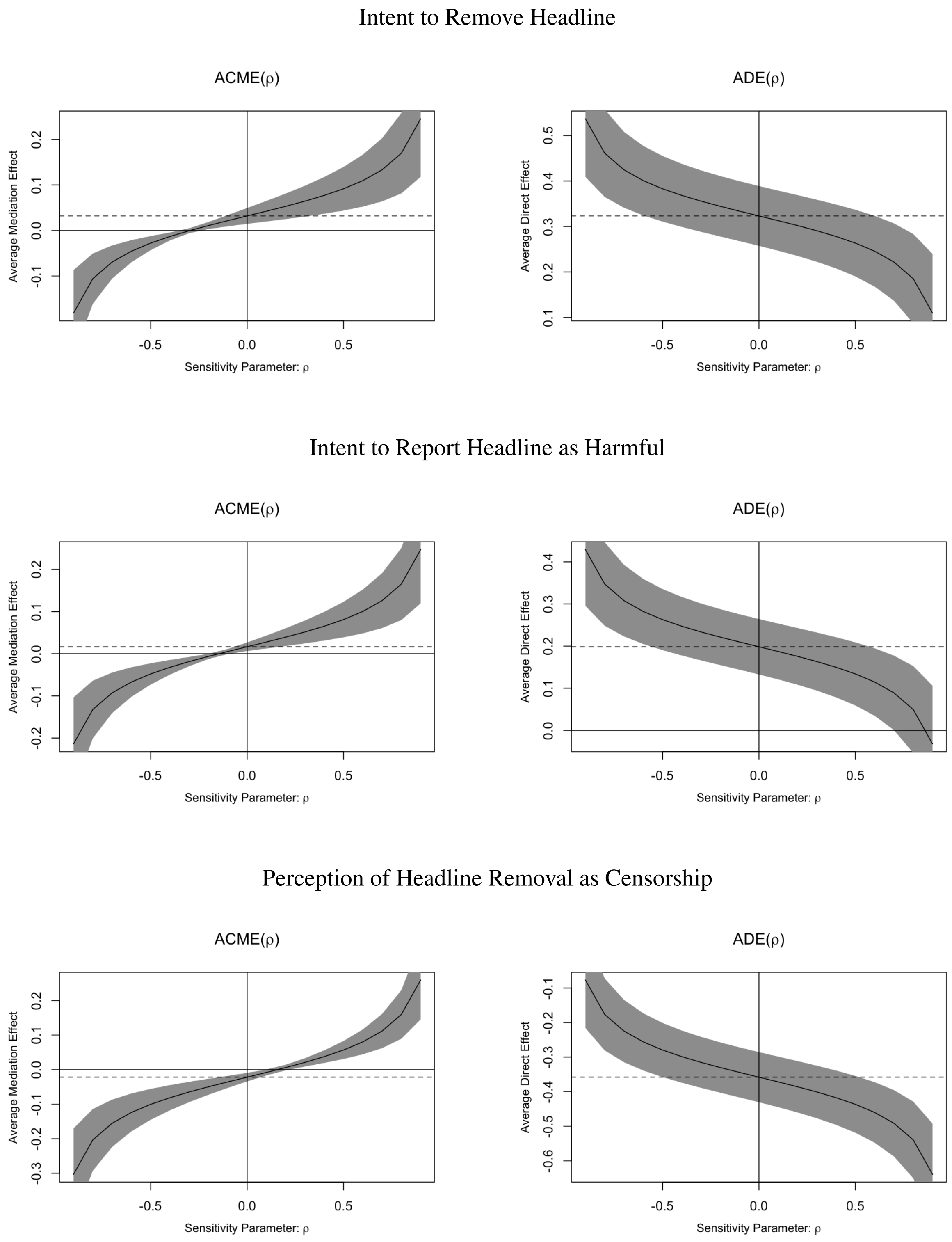

S2.3 Frequency of Censorship-Related Keywords in Congressional Speeches

Democrats and Republicans have used censorship-related keywords at different frequencies over time. To illustrate this, we analyzed Congressional speeches by Democrats and Republicans from the 46th to the 116th United States Congresses. Using tokenized speeches based on (*73*), and annotating which party had the majority in the Senate and the House for a given Congressional term, Figure S21 shows the count of tokens containing "censor" over time. Congressional speech data were downloaded on August 3, 2022 from (*74*), and data on which party had the majority in the Senate or the House were downloaded on August 6, 2022 from (*75*). Unrelated tokens containing "censor", such as "licensor", were excluded. For the 107th Congress, different parties had the majority at different times, we chose the party that initially had the majority.

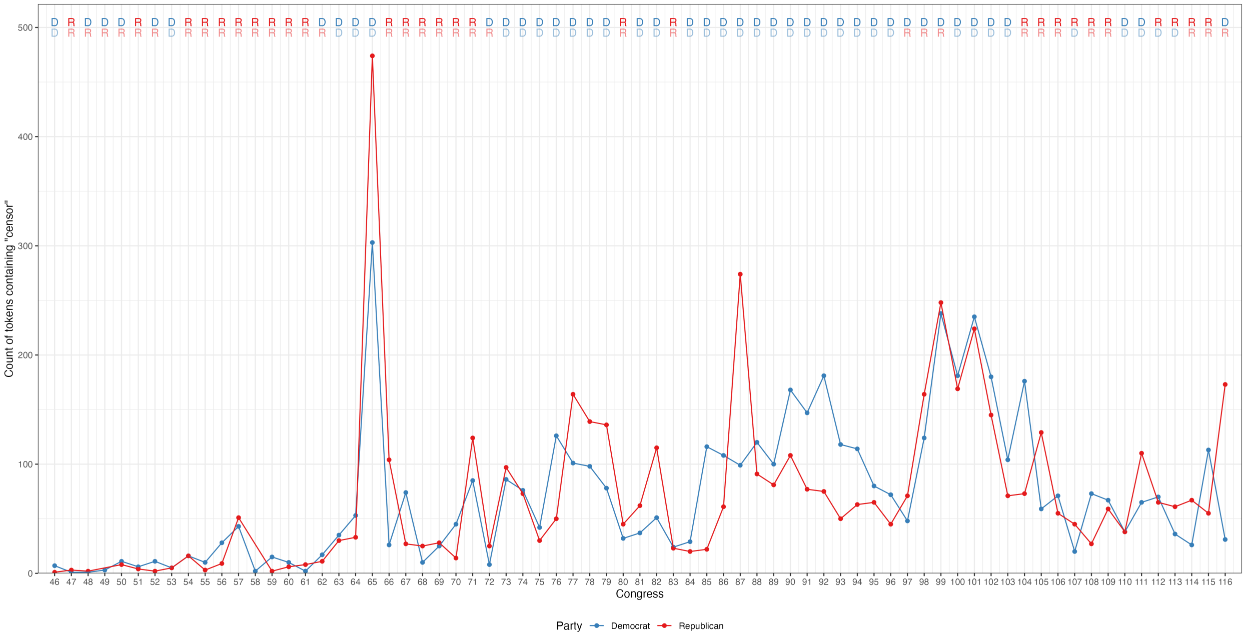

Fig. S21. Absolute frequency of tokens containing "censor" in Congressional Speeches by Congress and party. Letters indicate the majority party in a given Congress (darker font for House, lighter font for Senate).

Figure S21 shows a large burst in mentions of censorship during the 65th Congress during World War I, especially among Republicans. Another burst, led initially by Democrats but then by Republicans, comes during World War II (76th to 78th Congresses). The 87th Congress (1961–1963) sees a burst of discussion among Republicans about censorship, which is followed by Democrats talking more about censorship in relative terms from the 90th to 93rd Congresses (1967–1975). Finally, in the 116th Congress, we see a burst of discussion of censorship among Republicans (2019–2021).

REFERENCES AND NOTES

1. World Economic Forum, "The global risks report 2023" (Tech. Rep. 18, World Economic Forum, 2023).

2. Pew Research Center, "Climate change remains top global threat across 19-country survey" (Tech. Rep., Pew Research Center, 2022).

3. L. Silver, "Americans see different global threats facing the country now than in March 2020" (Tech. Rep., Pew Research Center, 2022).

4. D. E. Bambauer, *What Does the Day After Section 230 Reform Look Like?* (Brookings Institution, 2021).

5. T. Lorenz, *How the Biden Administration Let Right-Wing Attacks Derail Its Disinformation Efforts* (The Washington Post, 2022).

6. K. Ruggeri, B. Većkalov, L. Bojanić, T. L. Andersen, S. Ashcroft-Jones, N. Ayacaxli, P. Barea-Arroyo, M. L. Berge, L. D. Bjørndal, A. Bursalıoğlu, V. Bühler, M. Čadek, M. Çetinçelik, G. Clay, A. Cortijos-Bernabeu, K. Damnjanović, T. M. Dugue, M. Esberg, C. Esteban-Serna, E. N. Felder, M. Friedemann, D. I. Frontera-Villanueva, P. Gale, E. Garcia-Garzon, S. J. Geiger, L. George, A. Girardello, A. Gracheva, A. Gracheva, M. Guillory, M. Hecht, K. Herte, B. Hubená, W. Ingalls, L. Jakob, M. Janssens, H. Jarke, O. Kácha, K. N. Kalinova, R. Karakasheva, P. R. Khorrami, Žan Lep, S. Lins, I. S. Lofthus, S. Mamede, S. Mareva, M. F. Mascarenhas, L. M. Gill, S. Morales-Izquierdo, B. Moltrecht, T. S. Mueller, M. Musetti, J. Nelsson, T. Otto, A. F. Paul, I. Pavlović, M. B. Petrović, D. Popović, G. M. Prinz, J. Razum, I. Sakelariev, V. Samuels, I. Sanguino, N. Say, J. Schuck, I. Soysal, A. L. Todsen, M. R. Tünte, M. Vdovic, J. Vintr, M. Vovko, M. A. Vranka, L. Wagner, L. Wilkins, M. Willems, E. Wisdom, A. Yosifova, S. Zeng, M. A. Ahmed, T. Dwarkanath, M. Cikara, J. Lees, T. Folke, The general fault in our fault lines. *Nat. Hum. Behav.* **5**, 1369–1380 (2021).

7. A. Bradford, The brussels effect. *Northwest. Univ. Law Rev.* **107**, 1–68 (2012).

8. R. Jiménez Durán, "The economics of content moderation: Theory and experimental evidence

from hate speech on Twitter.” George J. Stigler Center for the Study of the Economy & the State Working Paper No. 324, University of Chicago, Chicago, 7 November 2023.

9. E. Henry, E. Zhuravskaya, S. Guriev, Checking and Sharing Alt-Facts. *Am. Econ. J. Econ. Policy*. **14**, 55–86 (2022).

10. A. Kozyreva, S. M. Herzog, S. Lewandowsky, R. Hertwig, P. Lorenz-Spreen, M. Leiser, J. Reifler, Resolving content moderation dilemmas between free speech and harmful misinformation. *Proc. Natl. Acad. Sci. U.S.A.* **120**, e2210666120 (2023).

11. N. M. Lindner, B. A. Nosek, Alienable speech: Ideological variations in the application of free-speech principles. *Polit. Psychol.* **30**, 67–92 (2009).

12. J. G. Bullock, G. Lenz, Partisan bias in surveys. *Annu. Rev. Polit. Sci.* **22**, 325–342 (2019).

13. M. Prior, G. Sood, K. Khanna, You cannot be serious: The impact of accuracy incentives on partisan bias in reports of economic perceptions. *Quart. J. Polit. Sci.* **10**, 489–518 (2015).

14. H. Allcott, M. Gentzkow, Social media and fake news in the 2016 election. *J. Econ. Perspect.* **31**, 211–236 (2017).

15. C. Batailler, S. M. Brannon, P. E. Teas, B. Gawronski, A signal detection approach to understanding the identification of fake news. *Perspect. Psychol. Sci.* **17**, 78–98 (2022).

16. D. Flynn, B. Nyhan, J. Reifler, The nature and origins of misperceptions: Understanding false and unsupported beliefs about politics. *Polit. Psychol.* **38**, 127–150 (2017).

17. M. Jakesch, M. Koren, A. Evtushenko, M. Naaman, The role of source, headline and expressive responding in political news evaluation. https://ssrn.com/abstract=3306403 (2018).

18. C. S. Traberg, S. van der Linden, Birds of a feather are persuaded together: Perceived source credibility mediates the effect of political bias on misinformation susceptibility. *Personal. Individ. Differ.* **185**, 111269 (2022).

19. S. C. Rhodes, Filter bubbles, echo chambers, and fake news: How social media conditions

individuals to be less critical of political misinformation. *Polit. Commun.* **39**, 1–22 (2022).

20. J. Roozenbeek, R. Maertens, S. M. Herzog, M. Geers, R. Kurvers, S. Mubashir, S. Van Der Linden, Susceptibility to misinformation is consistent across question framings and response modes and better explained by myside bias and partisanship than analytical thinking. *Judgm. Decis. Mak.* **17**, 547–573 (2022).

21. B. Gawronski, Partisan bias in the identification of fake news. *Trends Cogn. Sci.* **25**, 723–724 (2021).

22. A. Ashokkumar, S. Talaifar, W. T. Fraser, R. Landabur, M. Buhrmester, Á. Gómez, B. Paredes, W. B. Swann Jr., Censoring political opposition online: Who does it and why. *J. Exp. Soc. Psychol.* **91**, 104031 (2020).

23. J. J. Van Bavel, A. Pereira, The partisan brain: An identity-based model of political belief. *Trends Cogn. Sci.* **22**, 213–224 (2018).

24. Z. Kunda, The case for motivated reasoning. *Psychol. Bull.* **108**, 480–498 (1990).

25. C. G. Lord, L. Ross, M. R. Lepper, Biased assimilation and attitude polarization: The effects of prior theories on subsequently considered evidence. *J. Pers. Soc. Psychol.* **37**, 2098–2109 (1979).

26. C. S. Taber, M. Lodge, Motivated skepticism in the evaluation of political beliefs. *Am. J. Polit. Sci.* **50**, 755–769 (2006).

27. U. K. H. Ecker, S. Lewandowsky, J. Cook, P. Schmid, L. K. Fazio, N. Brashier, P. Kendeou, E. K. Vraga, M. A. Amazeen, The psychological drivers of misinformation belief and its resistance to correction. *Nat. Rev. Psychol.* **1**, 13–29 (2022).

28. G. Pennycook, D. G. Rand, Lazy, not biased: Susceptibility to partisan fake news is better explained by lack of reasoning than by motivated reasoning. *Cognition* **188**, 39–50 (2019).

29. G. Pennycook, Z. Epstein, M. Mosleh, A. A. Arechar, D. Eckles, D. G. Rand, Shifting

attention to accuracy can reduce misinformation online. *Nature* **592**, 590–595 (2021).

30. L. Huddy, *The Oxford Handbook of Political Psychology*, L. Huddy, D. Sears, J. Levy, Eds. (Oxford Univ. Press, ed. 2, 2013), pp. 737–773.

31. S. Iyengar, S. J. Westwood, Fear and loathing across party lines: New evidence on group polarization. *Am. J. Polit. Sci.* **59**, 690–707 (2015).

32. S. Iyengar, Y. Lelkes, M. Levendusky, N. Malhotra, S. J. Westwood, The origins and consequences of affective polarization in the united states. *Annu. Rev. Polit. Sci.* **22**, 129–146 (2019).

33. F. Chopra, I. Haaland, C. Roth, "The demand for news: Accuracy concerns versus belief confirmation motives," Working Paper 01/2023, NHH Department of Economics, 12 January 2023.

34. K. Crawford, T. Gillespie, What is a flag for? Social media reporting tools and the vocabulary of complaint. *New Media Soc.* **18**, 410–428 (2016).

35. M. Feinberg, R. Willer, From gulf to bridge *Pers. Soc. Psychol. Bull.* **41**, 1665–1681 (2015).

36. L. Silver, P. van Kessel, "Both Republicans and Democrats prioritize family, but they differ over other sources of meaning in life" (Tech. Rep., Pew Research Center, 2021).

37. A. Campbell, P. E. Converse, W. E. Miller, D. E. Stokes, *The American Voter* (Univ. of Chicago Press, 1980).

38. J. Duckitt, C. G. Sibley, Personality, ideology, prejudice, and politics: A dual-process motivational model. *J. Pers.* **78**, 1861–1894 (2010).

39. P. Goren, Party identification and core political values. *Am. J. Polit. Sci.* **49**, 881–896 (2005).

40. J. Graham, J. Haidt, B. A. Nosek, Liberals and conservatives rely on different sets of moral foundations. *J. Pers. Soc. Psychol.* **96**, 1029–1046 (2009).

41. J. T. Jost, C. M. Federico, J. L. Napier, Political ideology: Its structure, functions, and elective affinities. *Annu. Rev. Psychol.* **60**, 307–337 (2009).

42. C. Ellis, J. A. Stimson, *Ideology in America* (Cambridge Univ. Press, 2012).

43. M. Fiorina, S. Abrams, J. Pope, *Culture War? The Myth of a Polarized America* (Pearson Longman, 2005).

44. N. McCarty, K. T. Poole, H. Rosenthal, *Polarized America: The Dance of Ideology and Unequal Riches* (MIT Press, 2016).

45. R. D. Fisher, S. Lilie, C. Evans, G. Hollon, M. Sands, D. DePaul, C. Brady, D. Lindbom, D. Judd, M. Miller, T. Hultgren, Political ideologies and support for censorship: Is it a question of whose ox is being gored? *J. Appl. Soc. Psychol.* **29**, 1705–1731 (1999).

46. Knight Foundation, Ipsos, "Free expression in America post-2020" (Tech. Rep., Knight Foundation, 2022).

47. Congressional Record, The growing concentration of media ownership. *Cong. Rec.* **149**, H4179–H4184 (2003).

48. Congressional Record, Censorship of conservative voices from big tech corporations. *Cog. Rec.* **169**, H614–H615 (2023).

49. Rubio Introduces Sec 230 Legislation to Crack Down on Big Tech Algorithms and Protect Free Speech (2021).

50. Governor Ron DeSantis Signs Bill to Stop the Censorship of Floridians by Big Tech (2021).

51. Congressional Record, Parents bill of rights act. *Cong. Rec.* **169**, H1348–H1383 (2023).

52. K. Imai, L. Keele, D. Tingley, A general approach to causal mediation analysis. *Psychol. Methods* **15**, 309–334 (2010).

53. K. Imai, L. Keele, T. Yamamoto, Identification, inference and sensitivity analysis for causal

mediation effects. *Statist. Sci.* **25**, 51–71 (2010).

54. R. Hense, C. Wright, The development of the attitudes toward censorship questionnaire[1]. *J. Appl. Soc. Psychol.* **22**, 1666–1675 (1992).

55. J. T. Crawford, J. M. Pilanski, Political Intolerance, Right and Left. *Polit. Psychol.* **35**, 841–851 (2014).

56. J. Esberg, Censorship as reward: Evidence from pop culture censorship in chile. *Am. Polit. Sci. Rev.* **114**, 821–836 (2020).

57. A. Rao, F. Morstatter, K. Lerman, Partisan asymmetries in exposure to misinformation. *Sci. Rep.* **12**, 15671 (2022).

58. G. Eady, T. Paskhalis, J. Zilinsky, R. Bonneau, J. Nagler, J. A. Tucker, Exposure to the Russian Internet Research Agency foreign influence campaign on Twitter in the 2016 US election and its relationship to attitudes and voting behavior. *Nat. Commun.* **14**, 62 (2023).

59. N. Grinberg, K. Joseph, L. Friedland, B. Swire-Thompson, D. Lazer, Fake news on Twitter during the 2016 U.S. presidential election. *Science* **363**, 374–378 (2019).

60. A. M. Guess, B. Nyhan, J. Reifler, Exposure to untrustworthy websites in the 2016 US election. *Nat. Hum. Behav.* **4**, 472–480 (2020).

61. M. Mosleh, D. G. Rand, Measuring exposure to misinformation from political elites on Twitter. *Nat. Commun.* **13**, 7144 (2022).

62. D. Nikolov, A. Flammini, F. Menczer, *Right and Left, Partisanship Predicts (Asymmetric) Vulnerability to Misinformation*. (Harvard Kennedy School Misinformation Review, 2021); doi.org/10.37016/mr-2020-55.

63. O. Yair, G. A. Huber, How robust is evidence of partisan perceptual bias in survey responses?. *Public Opin. Q.* **84**, 469–492 (2021).

64. J. G. Bullock, A. S. Gerber, S. J. Hill, G. A. Huber, Partisan bias in factual beliefs about

politics. *Quart. J. Polit. Sci.* **10**, 519–578 (2015).

65. A. J. Berinsky, Telling the truth about believing the lies? Evidence for the limited prevalence of expressive survey responding. *J. Polit.* **80**, 211–224 (2018).

66. G. Pennycook, D. G. Rand, Accuracy prompts are a replicable and generalizable approach for reducing the spread of misinformation. *Nat. Commun.* **13**, 2333 (2022).

67. F. Pradel, J. Zilinsky, S. Kosmidis, Y. *Theocharis, Do Users Ever Draw a Line? Offensiveness and Content Moderation Preferences on Social Media* (OSF, 2022).

68. E. Douek, Content moderation as systems thinking. *Harv. Law Rev.* **136**, 526–607 (2022).

69. M. Feinberg, R. Willer, Moral reframing: A technique for effective and persuasive communication across political divides. *Soc. Personal. Psychol. Compass.* **13**, e12501 (2019).

70. J. Honaker, G. King, M. Blackwell, Amelia II: A program for missing data. *J. Stat. Softw.* **45**, 1–47 (2011).

71. M. Mosleh, C. Martel, D. Eckles, D. G. Rand, Perverse consequences of debunking in a Twitter field experiment: Being corrected for posting false news increases subsequent sharing of low quality, partisan, and toxic content, in *Proceedings of the 2021 CHI Conference on Human Factors in Computing Systems*, Yokohama, Japan, 8 to 13 May 2021 (ACM, 2021), pp. 1–13.

72. D. Tingley, T. Yamamoto, K. Hirose, L. Keele, K. Imai, mediation: R Package for causal mediation analysis. *J. Stat. Softw.* **59**, 1–38 (2014).

73. D. Card, S. Chang, C. Becker, J. Mendelsohn, R. Voigt, L. Boustan, R. Abramitzky, D. Jurafsky, Computational analysis of 140 years of US political speeches reveals more positive but increasingly polarized framing of immigration. *Proc. Natl. Acad. Sci. U.S.A.* **119**, e2120510119 (2022).

74. D. Card, S. Chang, C. Becker, J. Mendelsohn, R. Voigt, L. Boustan, R. Abramitzky, D.

Jurafsky, Replication code and data for “Computational analysis of 140 years of US political speeches reveals more positive but increasingly polarized framing of immigration” [Dataset]; https://github.com/dallascard/us-immigration-speeches/ (2022).

75. History, Art & Archives, U.S. House of Representatives, *Party Government Since 1857* [Dataset] (History, Art & Archives, U.S. House of Representatives, 2022); https://history.house.gov/Institution/Presidents-Coinciding/Party-Government/.
